# Identification of quorum sensing-controlled genes in *Burkholderia ambifaria*

**DOI:** 10.1002/mbo3.67

**Published:** 2013-02-05

**Authors:** Annelise Chapalain, Ludovic Vial, Natacha Laprade, Valérie Dekimpe, Jonathan Perreault, Eric Déziel

**Affiliations:** INRS-Institut Armand-Frappier531 bd des Prairies, Laval, Quebec, H7V 1B7, Canada

**Keywords:** Antifungal molecules, *Burkholderia*, quorum sensing, random mutagenesis, secondary metabolites

## Abstract

The *Burkholderia cepacia* complex (Bcc) comprises strains with a virulence potential toward immunocompromised patients as well as plant growth–promoting rhizobacteria (PGPR). Owing to the link between quorum sensing (QS) and virulence, most studies among Bcc species have been directed toward QS of pathogenic bacteria. We have investigated the QS of *B. ambifaria*, a PGPR only infrequently recovered from patients. The *cepI* gene, responsible for the synthesis of the main signaling molecule *N*-octanoylhomoserine lactone (C_8_-HSL), was inactivated. Phenotypes of the *B*. *ambifaria* *cepI* mutant we observed, such as increased production of siderophores and decreased proteolytic and antifungal activities, are in agreement with those of other Bcc *cepI* mutants. The *cepI* mutant was then used as background strain for a whole-genome transposon-insertion mutagenesis strategy, allowing the identification of 20 QS-controlled genes, corresponding to 17 loci. The main functions identified are linked to antifungal and antimicrobial properties, as we have identified QS-controlled genes implicated in the production of pyrrolnitrin, burkholdines (occidiofungin-like molecules), and enacyloxins. This study provides insights in the QS-regulated functions of a PGPR, which could lead to beneficial potential biotechnological applications.

## Introduction

The *Burkholderia cepacia* complex (Bcc) encompasses genetically closely related bacteria, currently distributed into 17 validly named species (Vandamme and Dawyndt 2011). Bcc species carry large multireplicon genomes, giving them a remarkable potential to adapt to diverse ecological niches. Indeed, since the first ecological description of *B. cepacia* as an onion pathogen, Bcc strains have been isolated from soils, waters, rhizospheres, immunocompromised patients, and industrial products (Burkholder 1950; Mahenthiralingam et al. 2005; Vial et al. 2011). Two major reasons of concern for humans are that Bcc species can be efficient plant growth–promoting rhizobacteria (PGPR), but also represent a significant threat to the life of immunocompromised individuals, such as those suffering from cystic fibrosis (CF) (Govan et al. 2000). Tremendous advances in identification techniques and taxonomy have revealed that Bcc members are widely but heterogeneously distributed according to niches, some species displaying particularly high virulence potential toward CF patients, while others acting as efficient PGPR (Mahenthiralingam et al. 2008). Still, almost all Bcc species have been isolated from both environmental and clinical sources, suggesting that adaptation to a specific niche is not strictly linked to the affiliation of a particular species (Mahenthiralingam et al. 2008; Vial et al. 2011). Accordingly, as the environment is apparently a reservoir for life-threatening bacteria, the commercial use of Bcc strains is not recommended, and actually placed under a moratorium imposed by the U.S. Environmental Protection Agency (EPA) (Chiarini et al. 2006).

Quorum sensing (QS) is a bacterial cell-to-cell communication system, based on the release of signaling molecules in the microenvironment; when the bacterial population grows, the concentration of molecules increases, reaching a threshold that triggers regulation of target genes (Williams 2007). The first QS system was discovered in *Vibrio fischeri* and implicated the LuxI synthase, responsible for the production of an *N*-acylhomoserine lactone (AHL) as signal molecule, which binds its cognate transcriptional regulator LuxR (Engebrecht and Silverman 1984). Similar LuxRI-type QS systems, relying on AHL molecules, have since been identified in most Gram-negative bacteria. In Bcc species, a LuxRI-type system named CepRI exists (Lewenza et al. 1999). CepRI relies on *N*-octanoylhomoserine lactone (C_8_-HSL) and *N*-hexanoylhomoserine lactone (C_6_-HSL) as signal molecules, the former being generally the most abundant one (Lutter et al. 2001). Some phenotypes, such as production of siderophores, proteolytic activities, and biofilm formation, have been described to be placed under QS control in Bcc bacteria (Eberl 2006). As QS is typically implicated in the regulation of virulence factors, most studies have been primarily focused on pathogenic bacteria (Sokol et al. 2007). However, QS could be considered more generally as a mean for bacteria to sense and interact with their microenvironments (Williams 2007; Mellbye and Schuster 2011). Recent studies implicating nonpathogenic *Burkholderia* strains have demonstrated that QS is important for bacterial relationships within the rhizosphere, for interaction with plants as well as in polymicrobial communities (Chan et al. 2011; Suarez-Moreno et al. 2012).

The Bcc *Burkholderia ambifaria* species is mostly isolated from soils and is especially predominant in the rhizosphere of several crops (Coenye et al. 2001; Coenye and Vandamme 2003). The type strain *B. ambifaria* LMG19182^T^ (or AMMD) was isolated from the healthy pea rhizosphere and was thereafter used as a biocontrol agent (Coenye et al. 2001; Chiarini et al. 2006). This species is seldom isolated from CF patient, where it causes transient and nontransmissible infections (Chiarini et al. 2006; Mahenthiralingam et al. 2008). Nevertheless, the clinical strain AU0212 was shown to be clonal to AMMD, suggesting that the environment represents a reservoir for clinical strains (Payne et al. 2005). The QS system of *B. ambifaria* has not been studied in details. It was nevertheless included in a few studies comparing Bcc members, revealing that it also possesses a CepRI system, relying on C_6_-HSL and C_8_-HSL signal molecules (Lutter et al. 2001; Zhou et al. 2003). Proteolytic activity, biofilm formation, and virulence toward nematodes are QS-controlled phenotypes, but seem strain-dependent (Wopperer et al. 2006). Interestingly, production of antifungal molecules is also QS-controlled in this Bcc species (Zhou et al. 2003; Schmidt et al. 2009). We have carried out a phenotypic study and a whole-genome transposon mutagenesis screening to identify QS-controlled genes and functions of a clinical *B. ambifaria* strain we have previously reported (Vial et al. 2008, 2010). This study will contribute to the understanding on the functions regulated by QS in a poorly virulent Bcc species with great biotechnological properties.

## Experimental Procedures

### Bacterial strains and culture conditions

*Burkholderia ambifaria* strain HSJ1 was isolated from sputum of a CF patient (Vial et al. 2008). *Escherichia coli* SM10 λ *pir* (thi-1 thr leu tonA lacY supE recA::RP4-2-Tc::Mu Km^r^ λpir) was used as a pKNOCK-Cm vector donor for conjugation experiments (Simon et al. 1983; Alexeyev 1999). *Escherichia coli* SM10*pir*/pIT2 containing the Tn*5-*derivative IS*lacZ*/hah was used as donor for random whole-genome transposon-insertion mutagenesis (Jacobs et al. 2003). Unless otherwise specified, the strains were routinely cultured at 37°C in tryptic soy broth (TSB) (BD) with shaking (240 rpm) in a TC-7 roller drum (New Brunswick, Canada), or on TSB agar plates.

### Construction of the *cepI* and *cepR* mutants

The HSJ1 *cepI* and *cepR* mutants were constructed using a suicide pKNOCK-Cm vector, according to the gene knockout strategy described previously (Alexeyev 1999). Bamb_4118 (*cepI*) and Bamb_4116 (*cepR*) from the sequenced *B. ambifaria* AMMD strain (Winsor et al. 2008) were used as a template to design the primers (Table S1), which carry KpnI and XbaI restriction sites, respectively. The fragment was then cloned as previously described (Vial et al. 2008). Mutants (single cross-over) were selected on TSB agar with 40 μg/mL chloramphenicol, and 25 μg/mL gentamicin to select against the donor (Sigma-Aldrich, Oakville, ON, Canada).

### LC/MS-MS analyses for AHL production

Culture samples of HSJ1 wild-type (WT) and *cepI* strains were retrieved at different time points of growth curve; OD_600_ was measured and 5 mg/L of methanolic internal liquid chromatography/mass spectroscopy (LC/MS) standard 5,6,7,8-tetradeutero-4-hydroxy-2-heptylquinoline (HHQ-d_4_) were added to samples (Lépine and Déziel 2011). Culture samples were vortexed, and extracted twice with ethyl acetate (1:1, vol:vol), pooled and evaporated at 35°C under a gentle stream of nitrogen. The residue was then resuspended in acidified acetonitrile (solvent B; details presented in Supplemental Experimental Procedures) at 10× the initial concentration (Lépine and Déziel 2011). Samples were analyzed by high-performance liquid chromatography (HPLC; Waters 2795, Mississauga, ON, Canada) equipped with a C8 reverse-phase column (Eclipse XDB-C8, Agilent Technologies, Mississauga, ON, Canada), and the detector was a mass spectrometer (Quattro Premier XE, Waters). Analyses were carried out in the positive electrospray ionization (ESI+) mode, supplemented by the multiple reactions monitoring (MRM) mode (details presented in Data S1). Samples were prepared in triplicate from three different colonies for each strain, and experiments were carried out at least twice independently.

### Phenotypic assays

Siderophore production was determined with Chrome Azurol S (CAS) agar plates (Schwyn and Neilands 1987). Bacteria siderophores are visualized by an orange halo around the colonies. Proteolytic activity was determined with 1% skim milk agar plate (Vial et al. 2008). Cholesterol oxidase activity was assessed as described (Doukyu and Aono 2001), with slight modifications as the TSB agar plates contained 0.26% Triton X-100 and 0.68 mmol/L cholesterol (Sigma-Aldrich). Cholesterol oxidase activity is visualized by a turbid precipitate around colonies, corresponding to oxidation of cholesterol into 6β-hydroperoxycholest-4-en-3-one, which is poorly soluble. Hemolytic activity was estimated on 5% sheep blood agar plates (Quelab, Montreal, QC, Canada) and 5% human blood agar plates. Antifungal activity was investigated against *Pythium ultimum* (Dr Richard Bélanger, Université Laval, Québec, QC, Canada), *Rhizoctonia solani* Kühn (MUCL number 51654, BCC/MUCL, Louvain-La-Neuve, Belgium), and *Candida albicans*. Potato dextrose agar (PDA, BD) and malt agar (BD Difco, Mississauga, ON, Canada) plates were inoculated with agar plugs of *P. ultimum* and *R. solani*, respectively. An overnight preculture of *C. albicans* in TSB was homogeneously spread with a sterile swab on a TSB agar plate, and then incubated at 37°C for 24 h. TSB agar plates supplemented with 0.1% Congo Red (Sigma-Aldrich) are used to study the colonial morphology and the ability to bind the red pigment (Vial et al. 2010). For most of phenotypic assays described above, HSJ1 WT and *cepI* strains were grown overnight in TSB, the cultures were normalized to OD_600_ = 5 and 3 μL were spotted on different agar plates, incubated at 37°C for 24 h and 48 h. For the tests with *P. ultimum* and *R. solani*, plates were incubated at 25°C for 3 and 21 days, respectively. Congo Red agar plates were inoculated with 100 μL of overnight preculture normalized to OD_600_ = 5 and diluted until 10^−7^, incubated for 1 day at 37°C, and then allowed to grow at room temperature for 20 days. Each experiment was repeated at least twice independently. For the inhibition test against *B. multivorans*, an overnight culture of *B. multivorans* ATCC 17616 was diluted to OD_600_ = 1 in TSB, and then incorporated in TSB medium containing 7.5 g/L agar (100 μL bacterial suspension/100 mL soft agar medium). The bacterial strains were allowed to grow 24 h at 30°C in liquid Basal Salts Medium (Mahenthiralingam et al. 2011). The supernatants were collected and 100 μL were laid in wells made in plates overlaid with *B. multivorans*. *Burkholderia ambifaria* AMMD WT strain was used as a control for the production of enacyloxins (Mahenthiralingam et al. 2011) (data not shown). To estimate if the phenotype could be restored by exogenous addition of signal molecules, control plates were supplemented with 2 mg/L C_8_-HSL.

### Infection of *Drosophila*

Fruit flies were infected by needle pricking according to the protocol previously described (Castonguay-Vanier et al. 2010). Control solution was composed of 10 mmol/L MgSO_4_ supplemented with 500 μg/mL ampicillin (Sigma-Aldrich) to avoid infection with nonspecific bacteria. The same solution was used to dilute *B. ambifaria* HSJ1 and its *cepI* mutant, precultured in TSB, at a final OD_600_ of 2. Thirty-six flies distributed in three bottles were pricked with bacterial suspensions containing HSJ1 or its *cepI* mutant; twelve flies were also pricked with the control solution to assess that the mortality was not due to the injury. Fly survival was scored daily and survival curves were processed with GraphPad Prism 5 (GraphPad Software, Inc., San Diego, CA) to perform a statistical log-rank (Mantel–Cox) test.

### Transposition mutagenesis

The HSJ1 *cepI* mutant was used as background for random whole-genome transposon-insertion mutagenesis, by mating with *E. coli* SM10*pir*/pIT2 containing Tn*5* IS*lacZ*/hah (Jacobs et al. 2003). Transconjugant cells were selected by plating on TSB agar containing 125 μg/mL tetracycline (Fisher Scientific, Ottawa, ON, Canada), 25 μg/mL gentamicin, 40 μg/mL of 5-bromo-4-chloro-3-indolyl-d-galactoside (X-gal, GoldBio, St. Louis, MO), and 2 mg/mL C_8_-HSL. Candidate colonies (2496) producing β-galactosidase activity, hence having a transposon inserted with the *lacZ* gene under the control of an expressed promoter, were then transferred to identical TSB agar plates but without C_8_-HSL. We found that 275 (11%) colonies produced a blue pigment, from pale to intense coloration, and that displayed a modification of the pigment production according to the absence of C_8_-HSL. These 275 candidates were then further verified by liquid β-galactosidase activity assay with *o*-nitrophenyl-β-d-galactopyranoside (ONPG, Thermo Fisher Scientific, Nepean, ON, Canada) (Miller 1972). Results obtained at four time points of the growth curve with C_8_-HSL were compared to those of the control (without C_8_-HSL). Finally, 43 transposants that differentially expressed β-galactosidase activity according to the presence of C_8_-HSL, both in solid and liquid media, were kept for further analyses.

### Identification of the transposon insertion sites

Insertion sites of the transposon were successfully determined for 40 mutants, mostly by two-stage semi-degenerate polymerase chain reaction (PCR) (Jacobs et al. 2003). As this approach failed for a few candidates, insertion sites were determined using another protocol (Lewenza et al. 2005), with modifications. Briefly, genomic DNA from mutants was extracted by bacterial DNA extraction kit (EZNA, Omega Bio-tek Norcross, GA). About 500 ng of genomic DNA were then digested for 2 h at 37°C by 10 U of AatII (New England Biolabs Ltd., Whitby, ON, Canada) or 7.5 U of PstI (Amersham Pharmacia Biotech Inc., Piscataway, NJ), in order to generate fragments susceptible to include the left or the right side of the transposon, respectively. Digestions were stopped by heat inactivation, and the DNA then circularized using T4 DNA Ligase (Rapid DNA Ligation Kit, Fermentas, Thermo Fisher Scientific), 30 min at room temperature. After 20 min of inactivation at 65°C, ligation products were used as template for an inverse PCR reaction, using the primers ISLacOut1F and ISLacOut1R, or ISLacOut2F and ISLacOut2R, depending if the template had been initially digested by AatII or PstI, respectively (Table S1). Amplification products were all sequenced at The McGill University and Génome Québec Innovation Centre (Montreal, Canada). Sequencing results were compared to the genomes of *B. ambifaria* AMMD and MC40-6 strains using BLAST (Winsor et al. 2008).

### Beta-galactosidase activity

Selected identified transposants were submitted to an additional β-galactosidase assay (Miller 1972). Conditions of the test were standardized, as differential activity in absence (control) or presence of C_8_-HSL was monitored for all mutants at two time points of the growth curve, around OD_600_ = 2 and 5. Each measure was done in triplicate, and all results were standardized as percentage of the control. For β-galactosidase assays performed on solid medium, 100 μL of an overnight culture diluted to OD_600_ = 0.1 were spread onto a 0.2 μm polycarbonate filter laid on TSB agar plates, supplemented or not with C_8_-HSL. After incubation for 26 h at 37°C, the filters were collected and the protocol for liquid cultures was followed, except that a total protein extraction followed by a Bradford assay was used instead of the OD_600_ to estimate bacterial growth.

### Quantitative reverse-transcription PCR experiments

Cultures were allowed to grow until around DO_600_ = 4, corresponding to the end of exponential growth phase. Total RNA was extracted with the RiboPure Bacteria kit (Ambion Life Technologies Inc., Burlington, ON, Canada). Extractions were done at least in triplicate, and twice independently. Concentration and purity of samples were assayed on a ND-1000 Nanodrop, and absence of degradation was confirmed on 1% agarose gel. Quantitative reverse-transcription PCR experiments were performed on a Rotor-Gene 6000 thermocycler (Corbett, Qiagen Inc., Toronto, ON, Canada), using the qScript One-Step qRT-PCR kit (Quanta BioSciences, Inc., Gaithersburg, MD), according to the manufacturer's protocol, with slight modifications (Data S1). The reference gene was *ndh* (Subsin et al. 2007). Primers used for mRNA amplification are presented in Table S1, and the amplification procedure in Table S2. Gene expression differences between HSJ1 and its *cepI* mutant were calculated using the 2(−ΔΔ(CT)) formula (Livak and Schmittgen 2001); a threshold of 0.5 was arbitrary chosen as significant.

### Bioinformatics search for putative *cep* boxes

Comparative genomics was used to predict *cep* boxes. Three different *cep* box consensus from previous reports (Chambers et al. 2006; Wei et al. 2011) were used in order to write three RNAMotif descriptors (Macke et al. 2001). A search in all the *Burkholderia* genomes (Winsor et al. 2008) with relaxed parameters provided thousands of hits. These were then compared between the corresponding hits of intergenic regions next to orthologous genes, with 18–24 extra nucleotides on each side. A combination of “*cep* box” score and conservation across the *Burkholderia* genus was used to assess significance of the predicted *cep* box (details in Data S1).

## Results

### A cepI mutant of *B. ambifaria* strain HSJ1 is defective in C_6_-HSL and C_8_-HSL production

**Figure 1:**
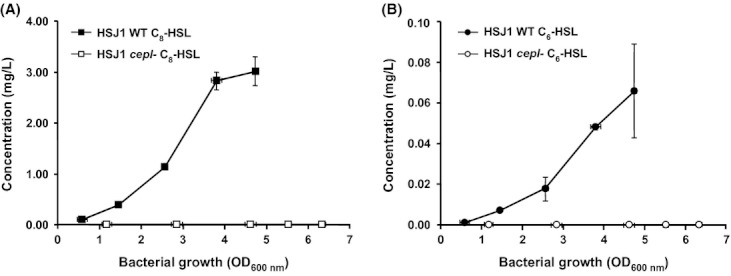
*N*-acylhomoserine lactone (AHL) production in *Burkholderia ambifaria* wild-type strain HSJ1 and its *cepI* mutant. The kinetics of production of (A) C_8_-HSL and (B) C_6_-HSL were measured using LC-MS/MS. Results are expressed as means ± standard deviations (SD) for triplicate assays.

Previous studies have shown that Bcc members possess a LuxI-type synthase CepI, which produces C_6_- and C_8_-HSL signal molecules, and these AHL have also been identified in *B. ambifaria* strains (Lutter et al. 2001; Zhou et al. 2003). We have used LC/MS-MS to measure AHL production in the clinical WT strain HSJ1; this strain produces mainly C_8_-HSL, reaching 3 mg/L under our experimental conditions (Fig. 1A) and also traces of C_6_-HSL, in a range close to 0.06 mg/L (Fig. 1B). As expected, the mutant of the *cepI* ortholog in the HSJ1 strain produces neither C_8_-HSL nor C_6_-HSL (Fig. 1). For following experiments, we focused on C_8_-HSL, as it is the most abundant and efficient ligand binding the transcriptional regulator CepR (Aguilar et al. 2003).

### *Burkholderia ambifaria* HSJ1 cepI mutant displays phenotypic modifications

**Figure 2:**
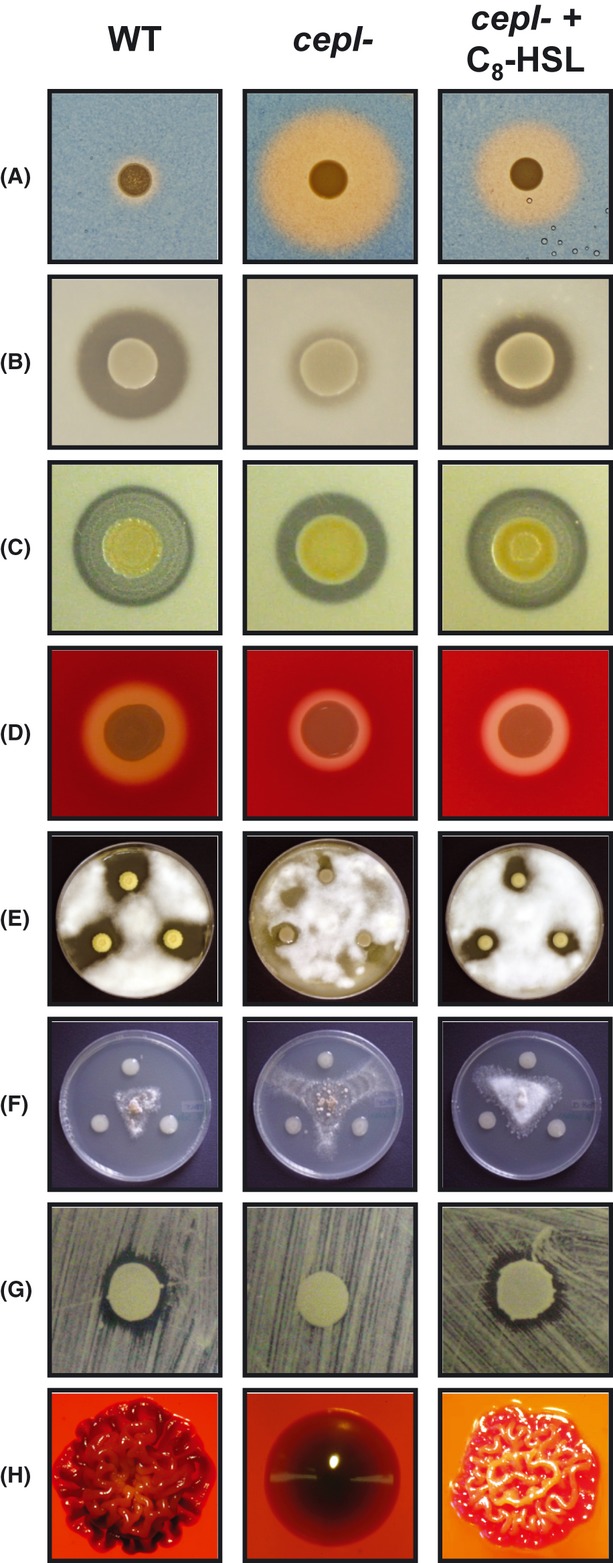
Phenotypic differences between *Burkholderia ambifaria* wild-type strain HSJ1 and its *cepI* mutant. (A) Siderophore production after 24 h on Chrome Azurol S (CAS) agar plates, (B) proteolytic activity after 24 h on milk agar plates, (C) cholesterol oxidase activity after 48 h on cholesterol agar plates, (D) hemolytic activity after 24 h on 5% human blood agar plates, (E) antifungal activity against *Pythium ultimum* after 3 days on potato dextrose agar (PDA) agar plates, (F) antifungal activity against *Rhizoctonia solani* after 21 days on malt agar plates, (G) antifungal activity against *Candida albicans* after 24 h on tryptic soy broth (TSB) agar plates, (H) colonial morphology and ability to bind pigment after 21 days on Congo Red agar plates**.**

We then looked at phenotypes of *B. ambifaria* HSJ1 WT strain and its *cepI* mutant, to compare with other Bcc *cepI* mutants described in the literature, or to reveal phenotypes associated with *B. ambifaria*. The inactivation of *cepI* is associated with the overproduction of siderophores in the Bcc *B. cenocepacia* K56-2 (Lewenza et al. 1999). This phenotype was thus assessed on CAS agar plates and, after 24 h, the orange-colored halo around HSJ1 *cepI* mutant colonies was about 27-fold larger (339.05 ± 8.54 mm^2^) than the one of the WT strain (12.42 ± 4.87 mm^2^) (Fig. 2A). The WT phenotype was partially restored when C_8_-HSL was added, as the halo decreased at 191.20 ± 14.21 mm^2^. Another phenotype generally described in Bcc *cepI* mutants is the decreased secretion of extracellular proteases (Aguilar et al. 2003; Kooi et al. 2006), which is assessed on milk agar plates. After 24 h, clearing halos around *cepI* mutant colonies (21.94 ± 6.04 mm^2^) were roughly 20% of those of HSJ1 WT strain colonies (119.18 ± 8.48 mm^2^) (Fig. 2B). The difference was reduced by half (49.04 ± 2.45 mm^2^) when C_8_-HSL was added to the medium. The cholesterol oxidase activity has been previously reported in HSJ1 WT strain (Vial et al. 2010). We sought if this activity could be modified in the HSJ1 *cepI* mutant and indeed observed that the characteristic zone of precipitate indicative of cholesterol oxidase activity is present around WT strain colonies, but not around *cepI* mutant colonies, unless C_8_-HSL is added to the medium (Fig. 2C). The hemolytic activity was emphasized when *B. ambifaria* was first described as a Bcc species (Coenye et al. 2001); after 48 h, the beta-hemolytic halo on human blood agar plate around WT strain colonies was 41.33 ± 6.32 mm^2^, whereas the one of the *cepI* mutant was about half (18.21 ± 2.44 mm^2^), except when C_8_-HSL was added (31.71 ± 3.74 mm^2^) (Fig. 2D). The hemolytic activity on sheep blood agar plates gives the same kind of results (data not shown).

Another important phenotype associated with *B. ambifaria* strains is the inhibitory activity against a broad spectrum of fungi, and some studies reported the influence of QS on such PGPR properties (Zhou et al. 2003; Schmidt et al. 2009). We have thus evaluated the antifungal activity of HSJ1 WT strain and its *cepI* mutant against *P. ultimum* (Fig. 2E), *R. solani* (Fig. 2F), and *C. albicans* (Fig. 2G), which were previously described to be sensitive to *B. ambifaria* antifungal properties (Cain et al. 2000; Zhou et al. 2003). In each situation, HSJ1 WT strain displayed an antifungal activity, which was reduced or even absent in the *cepI* mutant and restored (at least partially) if the medium contained C_8_-HSL.

We also looked at the colony morphology on Congo Red agar plates, which was previously used in the phenotypic characterization of HSJ1 WT strain (Vial et al. 2010). Although HSJ1 WT strain colonies are wrinkled and able to bind the red pigment, *cepI* mutant colonies are smooth, but still able to bind the pigment (Fig. 2H).

It is noteworthy that for all these phenotypic tests, the HSJ1 *cepR* mutant was also investigated; as expected, its phenotypes were similar to those of *cepI* mutant; therefore, the remaining experiments were only performed with the latter.

**Figure 3:**
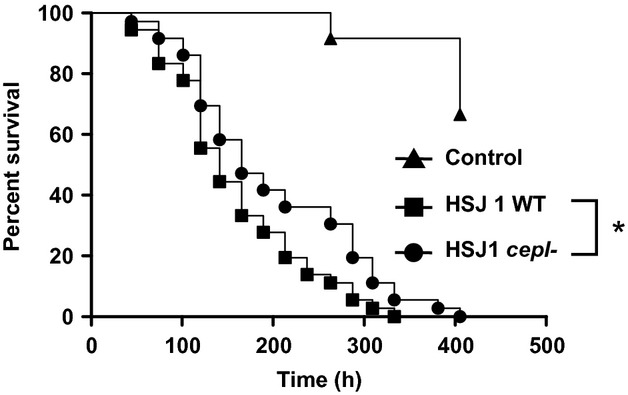
Virulence of *Burkholderia ambifaria* wild-type strain HSJ1 and its *cepI* mutant toward *Drosophila melanogaster*. Mortality was scored daily. *P < 0.05.

Finally, as several of these factors could be associated with virulence, we used the *Drosophila melanogaster* host model (Castonguay-Vanier et al. 2010) to assess the virulence of HSJ1 WT and its *cepI* mutant. As we have previously reported with another *B. ambifaria* strain, this species is among the less virulent Bcc species toward fruit flies (Castonguay-Vanier et al. 2010). The median survival was 141 h for HSJ1 WT strain (Fig. 3), but 165 h for the *cepI* mutant, which thus is significantly less virulent (*P* < 0.05).

For phenotypes for which a comparison with other Bcc *cepI* mutant is possible, the HSJ1 *cepI* mutant displays phenotypic modifications in compliance with those already reported.

### Twenty QS-controlled genes identified by whole-genome transposon-insertion mutagenesis

To identify genes whose expression is influenced by C_8_-HSL, we employed the *cepI* mutant as background for a random whole-genome transposon-insertion mutagenesis. Forty mutants successfully sequenced following the screening revealed 20 QS-regulated genes, corresponding to 17 loci. The identification of these genes was realized by BLAST searches against the genomes of *B. ambifaria* AMMD and MC40-6 strains (http://www.burkholderia.com) (Winsor et al. 2008). The results were generally identical for the two strains, but e-values were often better with the AMMD strain; furthermore, for several sequences we obtained results only with the AMMD strain. Since this was suggesting that HSJ1 is more closely related to AMMD than to MC40-6, we have only considered the AMMD strain for the remaining of this study.

The identified genes are thus presented in Table 1 according to the AMMD locus codes (Winsor et al. 2008). These genes are homogeneously distributed along the three chromosomes, and some of them were selected several times. Notably, Bamb_4726 (*prnA*), the first gene of the operon responsible for synthesis of pyrrolnitrin, a potent antifungal compound previously reported to be QS-controlled (el-Banna and Winkelmann 1998; Schmidt et al. 2009), was picked up six times, including four times at the same insertion site. For two mutants, the insertion of the transposon is located in the intergenic region upstream of the corresponding gene. Predicted operons are indicated, including the position of genes identified in the screening (Winsor et al. 2008); for example, Bamb_6469 and Bamb_6472 are predicted to belong to the same operon, as well as Bamb_6476 and Bamb_6477, all four belonging to the gene cluster implicated in the synthesis of occidiofungins, an antifungal compound initially described in *B. contaminans* MS14 (Lu et al. 2009).

**Table 1:** Genes identified in the screening for quorum sensing regulation

Chromosome	Genes	Strand^1^	Transposon position (redundancy)^2^	Position of gene in operon	Orthologs^3^	C_8_-HSL-induced regulation^4^	Function or predicted function of the genes [compound]^5^
1	Bamb_1141	−	27	1/2	10	M	Heat shock protein Hsp20
1	Bamb_2172^9^	−	750 (3)	–	30	M	Dihydrolipoamide dehydrogenase
1	Bamb_2297^9^	+	1096	2/2	29	M	Sulfate transporter
			1158				
1	Bamb_2378	−	91	–	29	R	Spermidine synthase-like protein (SpeE)
1	Bamb_2404	+	131	–	4	M	Hypothetical protein
1	Bamb_2520	−	235	5/6	30	R	Sulfate adenylyltransferase, large subunit (cysN)
1	Bamb_3128	+	−170	–	22	I	Hypothetical protein
2	Bamb_3350	+	572 (2)	6/6	30	R	Tryptophan synthase subunit alpha (trpA)
2	Bamb_3366	+	−30	–	18	I	Hypothetical protein
2	Bamb_4578^9^	+	385	2/3	24	M	Hypothetical protein
2	Bamb_4726^9^	+	103	1/4	10	I	Tryptophan halogenase (prnA) [pyrrolnitrin]
			273				
			432 (4)				
2	Bamb_5109^9^	+	113	–	3	I	Hypothetical protein
2	Bamb_5535^9^	−	−108	1/2	29	M	ElaB (protein of unknown function DUF883)
3	Bamb_5622	+	70	–	22	I	PRC-barrel domain-containing protein
3	Bamb_5911	+	229	2/2	0	I	LuxR family transcriptional regulator [enacyloxins]
3	Bamb_5925	−	1463	2/10	2	I	Beta-ketoacyl synthase [enacyloxins]
3	Bamb_6465	−	343	–	10	I	FAD linked oxidase domain-containing protein
3	Bamb_6469^6^	−	367 (2)	4/4	1	I	Cyclic peptide transporter [occidiofungins]
			402				
3^6^	Bamb_6472^6^,^8^	−	4578	1/4	70	I	Amino acid adenylation domain-containing protein [occidiofungins]
	Bamb_6476^7^,^8^	−	10962	3/6	38		Amino acid adenylation domain-containing protein [occidiofungins]
3	Bamb_6477^7^	−	204	2/6	1	I	Short-chain dehydrogenase/reductase SDR [occidiofungins]

1 The sign + or − refers to the DNA strand encoding the gene identified by the BLAST searches (http://www.burkholderia.com).

2 The position of transposon is indicated in base pairs (bp) since the predicted translational start site; the number between brackets indicates how often the transposon was identified at the same insertion site.

3 Orthologs indexed in the *Burkholderia* website (http://www.burkholderia.com).

4 Effect of C_8_-HSL on gene expression deduced from LacZ reporter assay; R, repression; I, induction; M, moderate effect.

5 Function or predicted function listed in the *Burkholderia* website; if genes are included in clusters, the name of the resulting compound is indicated between square brackets.

6 Bamb_6469 and Bamb_6472 belong to the same predicted operon.

7 Bamb_6476 and Bamb_6477 belong to the same predicted operon.

8 The BLAST search did not allow to discriminate Bamb_6472 from Bamb_6476 for this mutant; thus, both genes are indicated.

9 Genes for which a putative *cep* box has been predicted.

We have also verified whether genes identified in the screening have predicted orthologs among the 27 complete and five incomplete genomes present on the *Burkholderia* website (Winsor et al. 2008). Some genes have about 30 putative orthologs, and appear thus widespread among *Burkholderia* species, while other seem to be found only in Bcc members. Interestingly, Bamb_5911, which codes for a LuxR-type family transcriptional regulator located upstream of the cluster implicated in the biosynthesis of enacyloxins, an antimicrobial compound recently characterized in *B. ambifaria* AMMD (Mahenthiralingam et al. 2011), has only one ortholog, in *B. ubonensis*, which is not included in the *Burkholderia* website (Winsor et al. 2008).

**Figure 4:**
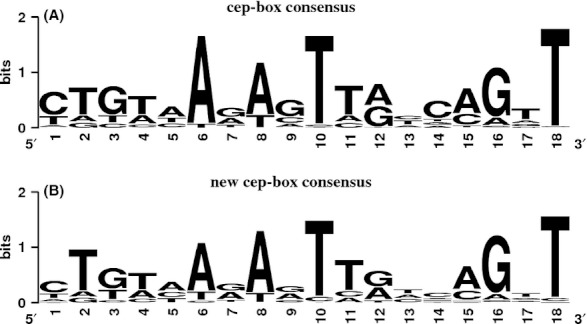
Consensus *cep* box. (A) The *cep* box consensus established by Chambers et al. (2006) and by Wei et al. (2011) were combined to obtain this sequence logo with weblogo (Crooks et al. 2004). (B) The putative *cep* boxes found in this screen (see Supporting Information) were combined with the consensus in (A) to obtain this sequence logo.

In order to gain insights in the type of regulation (direct or indirect) exerted by QS on genes identified in our screening, we performed a bioinformatics search for putative *cep* boxes in promoter regions. *cep* boxes are sequences recognized by the transcriptional regulator CepR to bind target promoters and modulate the transcription of genes; the presence of these *cep* boxes upstream of genes pleads in favor of a direct regulation by QS. Concerning the genes identified in the screening, our search predicts the presence of a putative *cep* box upstream from the transcriptional units of Bamb_2172, Bamb_2297, Bamb_4578, Bamb_4726, Bamb_5109, and Bamb_5535 (Table 1 and Fig. S1). The predictive *cep*-box sequence, derived from Chambers et al. (2006) and Wei et al. (2011), as well as a *cep* box derived from these data and the genes identified in our screening, is presented in Figure 4. Putative *cep* boxes upstream of genes not identified in the screening are presented in Figure S1.

### C_8_-HSL addition induces diverse modifications in reporter gene activities

**Figure 5:**
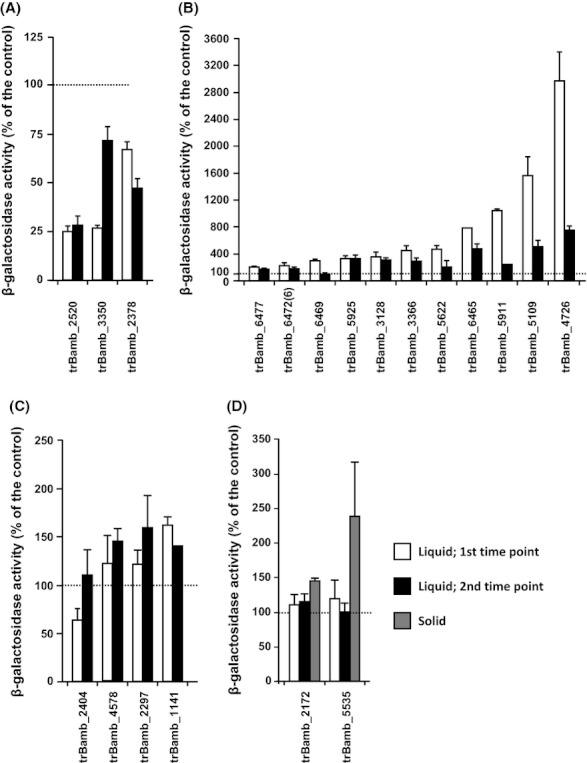
Expression of quorum sensing (QS)-regulated genes in response to C_8_-HSL. Transposon mutants in putative QS-regulated genes isolated in the *cepI* mutant background during the screening were tested for their β-galactosidase activity with or without adding of 2 mg/L C_8_-HSL. Cultures were sampled at two time points of the growth curve, and the activity calculated in Miller units; for (D) cultures were realized in solid medium. Results for transposants supplemented with C_8_-HSL are expressed in percent of the controls (transposants without C_8_-HSL), the latter being normalized at 100% and symbolized by the dotted line. Results are expressed as means ± SD for triplicate assays. (A) Highly reduced activity (twofold change of the control or less), (B) highly induced activity (twofold change of the control or more), (C) moderate activity after C_8_-HSL addition in liquid culture (±1.5-fold change of the control), (D) β-galactosidase activity after C_8_-HSL addition in solid medium.

To estimate the impact of C_8_-HSL addition on the regulation of the identified genes, their expression was measured at two different points of the growth of each mutant, around OD_600_ = 2 and OD_600_ = 5. Figure 5 shows the effect of adding 2 mg/L C_8_-HSL on the activity of the inserted reporters. We considered that mutants were strongly affected by C_8_-HSL addition if the reporter activity displayed at least a twofold change compared to their respective control (Fig. 5A and B); if the activity displayed only a 1.5-fold change, the mutants were considered moderately affected (Fig. 5C and D). Accordingly, panel A shows mutants which presented a marked decrease in reporter activity after C_8_-HSL addition (Fig. 5A). For instance, the mutant in which the transposon has interrupted the gene Bamb_2520 (renamed “trBamb_2520” to avoid confusion with the gene itself) displayed an activity nearly four times lower that the one of the control following C_8_-HSL addition. On the other hand, panel B shows mutants which displayed a strong increase of the reporter expression after C_8_-HSL addition, displaying activities ranging from two to almost 30 times higher than those of the control (Fig. 5B). The highest score of LacZ reporter activity was obtained with the mutant trBamb_4726, in which the operon coding for pyrrolnitrin is interrupted. The remaining mutants displayed a moderate modification of reporter expression after C_8_-HSL addition (Fig. 5C and D). Because the mutants were initially screened on agar plates, we reasoned the growth conditions might affect the expression of these genes. We thus submitted them to a β-galactosidase challenge on solid medium. After 26 h, the results obtained for the majority of the mutants were roughly the same as described in panel C (data not shown), except for two mutants presented in panel D, for which the LacZ reporter activity was significantly increased when mutants were grown on solid medium (Fig. 5D). The effects of C_8_-HSL addition on LacZ reporter activity are summarized in Table 1.

It is interesting to note that some mutants displayed a similar reporter gene activity at the two time points (such as trBamb_2520), whereas others were markedly different (such as trBamb_4726) (Fig. 5A and B), reflecting different patterns of regulation.

### qRT-PCR experiments confirm LacZ reporter data

To validate β-galactosidase activity results, we then performed real-time quantitative reverse-transcription PCR (qRT-PCR) experiments on a group of genes identified in the screening. We chose genes corresponding to mutants distributed in panels A and B, displaying thus a strong modification of activities. We have also included *zmpA* and *zmpB* (Bamb_3836 and Bamb_4475, respectively), previously described to be positively QS-controlled in *B. cenocepacia* (Kooi et al. 2006; Subsin et al. 2007), as well as Bamb_1196, which was identified in our screening but not confirmed in the β-galactosidase assays. The results presented in Figure S2 show the relative expression obtained in the HSJ1 *cepI* mutant compared to the HSJ1 WT strain. The level of twofold change was arbitrarily chosen as threshold of significant difference. For Bamb_6469, there is an apparent discrepancy between qRT-PCR experiments and *LacZ* reporter results. Actually, mRNA were extracted around OD_600_ = 4, which is closer to the second time point of β-galactosidase assays, for which trBamb_6469 displayed an activity similar to the one of the control (Fig. 5B). For the remaining tested genes, they all confirm data obtained with the LacZ fusions. Under the tested conditions, *zmpB* was so poorly expressed in the *cepI* mutant that it did not reach the 0.5 threshold used to calculate the Ct.

### Phenotypic confirmation of transposon mutants

**Figure 6:**
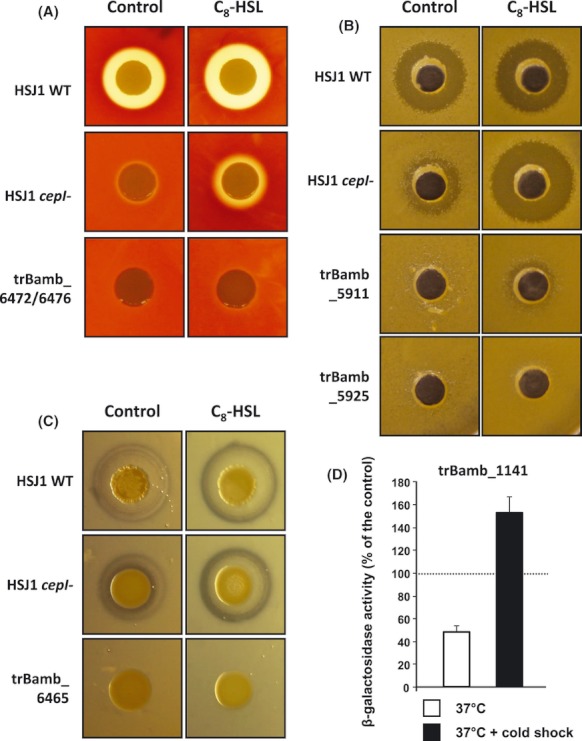
Phenotypic confirmation of transposon mutants. As the transposon mutants are all *cepI*/transposon mutants, C_8_-HSL was added in media to compensate the *cepI* impairment and observe only the effect of transposon mutation on predicted phenotypes. The following phenotypes are presented: (A) hemolytic activity on sheep blood agar, (B) anti-*Burkholderia multivorans* activity, (C) cholesterol oxidase activity on cholesterol agar plates, (D) effect of temperature on β-galactosidase activity for bacteria grown in tryptic soy broth (TSB) agar plates.

As the *cepI* mutant was used as background strain for the whole-genome transposon-insertion mutagenesis, all the resulting mutants are in fact double mutants, impaired in C_8_-HSL production and in the function coded by the gene interrupted by the transposon. To explore some phenotypes associated with genes presented in Table 1, the *cepI* deletion of the transposon mutants was compensated by C_8_-HSL addition in the media used for the phenotypic challenges. Among the genes identified in the screening, several are implicated in the production of pyrrolnitrin, enacyloxins, and occidiofungins (Table 1), which have antifungal/antibacterial activities. We first tested the corresponding mutants against *C. albicans*, *P. ultimum,* and *R. solani*. Except for Bamb_5925, for which a slight difference against *P. ultimum* was noted, all the transposon mutants behaved as the *cepI* mutant (Fig. S3). We therefore sought phenotypic tests providing a better discrimination of these mutants. Hemolytic properties were recently described for genes implicated in occidiofungins biosynthesis (Thomson and Dennis 2012). On sheep blood agar, trBamb_6469 and trBamb_6477 behave as the *cepI* mutant (data not shown), whereas trBamb_6472/6476 displays no hemolytic activity even with C_8_-HSL supplementation (Fig. 6A). A specific activity of enacyloxins was reported against *B. multivorans* (Mahenthiralingam et al. 2011); we thus tested the activity of trBamb_5911 and trBamb_5925 against this bacterium. It is interesting to note that the addition of C_8_-HSL in the medium allowed the *cepI* mutant to produce a greater inhibiting zone than the one of the WT; for transposon mutants, trBamb_5911 displayed a slight inhibiting zone when C_8_-HSL was added, whereas the inhibiting activity of trBamb_5925 was completely abolished (Fig. 6B).

Concerning the cholesterol oxidase activity, the *cepI* mutant is able to produce a clearing halo around the colony, but the characteristic precipitate linked to cholesterol oxidase activity is obtained only if C_8_-HSL is present (Figs. 2C and 6C). In contrast, the trBamb_6465 mutant’ colony produced neither clearing halo nor precipitate (Table 1 and Fig. 6C). Since Bamb_1141 encodes a heat shock protein HSP20 (Table 1), we ask whether it could exhibit a different LacZ reporter activity according to temperature. Indeed, if the growth of trBamb_1141 occurs in solid medium supplemented by C_8_-HSL and incubated only at 37°C, the β-galactosidase activity is half the one of the control, whereas it reaches around 150% of the control if the assay is preceded by a 6 h incubation at 4°C (Fig. 6D).

These experiments confirmed the phenotypes of mutants tested and brought some additional clues on the role of C_8_-HSL in the regulation.

## Discussion

*Burkholderia ambifaria* displays a remarkable potential as a PGPR and biocontrol agent, but like the other Bcc members, its commercial use is placed under a moratorium (Chiarini et al. 2006). The production of commercially interesting molecules in vitro through biotechnological processes requires the understanding of mechanisms that direct and regulate their biosynthesis, such as QS.

### Phenotypes of the HSJ1 cepI mutant

Several phenotypes classically associated with QS, such as siderophores production and proteolytic activity, are in fact disparately present and QS-controlled among Bcc members (Gotschlich et al. 2001; Huber et al. 2001; Aguilar et al. 2003). We report here that the *cepI* mutant of *B. ambifaria* strain HSJ1 overproduces siderophores, similarly to the *cepI* mutant of *B. cenocepacia* K56-2 (Lewenza et al. 1999). The HSJ1 *cepI* mutant has a decreased protease activity, as most of Bcc strains in absence of C_8_-HSL (Wopperer et al. 2006). This phenotype has been linked in *B. cenocepacia* to two QS-regulated metalloproteases, ZmpA and ZmpB (Gingues et al. 2005; Kooi et al. 2006). These proteases were also identified in *B. ambifaria* strain HSJ1 (Vial et al. 2010); we found here that *zmpA* and *zmpB* are strongly downregulated in the HSJ1 *cepI* mutant (Fig. S2 and data not shown), which probably explains the decreased proteolytic activity.

We have also looked at some phenotypes previously described in *B. ambifaria* HSJ1 WT strain (Vial et al. 2010), such as the colonial morphology on Congo Red agar plates. The colonial wrinkling is a QS-regulated character in HSJ1 strains; a such colony wrinkling has been linked to the QS-regulation of the exopolysaccharide Pel in *Pseudomonas aeruginosa* (Gupta and Schuster 2012). The secretion of a FAD-dependent cholesterol oxidase, identified as Bamb_6465 and correlated with a cholesterol-degrading activity, was also previously reported in *B. ambifaria* HSJ1 (Vial et al. 2010). We found here that this phenotype is positively controlled by QS (Fig. 2C), and confirmed that it is due to Bamb_6465, which is strongly QS-activated in *B. ambifaria* HSJ1 (Figs. 5B, 6C, and S2).

Another noteworthy phenotype is the beta-hemolytic activity, which was highlighted when *B. ambifaria* was initially described as a new Bcc species (Coenye et al. 2001). Factors implicated in such effects in Bcc members are poorly identified; however, a hemolytic compound named cepalycins, displaying also antifungal properties, has been previously isolated from the supernatant of *B. cepacia* JN106 (Abe and Nakazawa 1994). Similar hemolytic properties were recently reported for occidiofungins in *B. vietnamiensis* DBO1, compounds initially described for their antifungal activities in *B. contaminans* MS14 (Lu et al. 2009; Thomson and Dennis 2012). These dual activities likely result from the interaction of these extracellular molecules with cholesterol in the membrane; indeed, cepalycins were more inhibited by ergosterol than by cholesterol (Abe and Nakazawa 1994). Moreover, environmental strains of Bcc apparently display more hemolytic activity than clinical strains (Bevivino et al. 2002), which is coherent if hemolytic molecules are in fact antifungal molecules; this hypothesis is also compliant with the natural ecology of *B. ambifaria*. In our screening, we have identified three genes implicated in occidiofungins biosynthesis (Table 1). The mutant trBamb_6472/trBamb_6476 is impaired in hemolytic function (Fig. 6A), while trBamb_6469 behave as the *cepI* mutant (data not shown), which is in agreement with recent published data (Thomson and Dennis 2012). On the other hand, trBamb_6477 behaved also like the *cepI* mutant (data not shown); this discrepancy with the study of Thomson and Dennis (2012) could well be explained by the use of different Bcc species or by the compensation of the disrupted gene by another one with similar function.

Additionally, the antifungal activities of *B. ambifaria* strains against several fungi have been already described (Cain et al. 2000; Zhou et al. 2003), and implication of the QS-regulation for such antifungal activities has also been reported (Zhou et al. 2003; Schmidt et al. 2009). Accordingly, our phenotypic assay against *P. ultimum*, *R. solani,* and *C. albicans* demonstrated that *B. ambifaria* HSJ1, although being from clinical origin, exhibits antifungal activity, while its *cepI* mutant displays reduced (or even abolished) antifungal activities.

### High-throughput screening to identify new QS-regulated genes

Global approaches using high-throughput screenings have been developed to identify more rapidly and efficiently a wide range of QS-regulated genes, often with the aim to identify those coding for potential virulence factors. We have identified 20 QS-controlled genes employing a procedure derived from Chambers et al. (2006) that had permitted the identification of seven QS-controlled genes in *B. cenocepacia* K56-2. This approach does not allow to discriminate genes directly or indirectly controlled by QS (Wei et al. 2011). To partially circumvent this shortcoming, we have performed a bioinformatics search for the presence of putative *cep* boxes upstream of transcriptional units identified in the screening. *cep* boxes are short sequences upstream the promoter of target genes that allow CepR to recognize its chromosomal binding site. Although the *cep* box upstream of *cepI* is well conserved among Bcc members (Fig. S1), the conservation is less obvious upstream of other QS-controlled genes. We have used the consensus sequence described in *B. cenocepacia* by Chambers et al. (2006) to predict *cep* boxes in *B. ambifaria* HSJ1. We have notably identified a putative *cep* box upstream of *prnA* (Bamb_4726), which has been identified six times in our screening and for which the direct regulation by CepR has been experimentally demonstrated in *Burkholderia lata* 383 (Schmidt et al. 2009). A recent study has reported a different consensus sequence in *B. cenocepacia* K56-2, as well as the experimental demonstration of the direct regulation for two genes, BCAL0510 and BCAM1869 (Wei et al. 2011). BCAM1869 is an ortholog of Bamb_4117, which is located between *cepR* and *cepI*, and for which we have predicted a putative *cep* box (Fig. S1). On the other hand, BCAL0510 is an ortholog of Bamb_3128, which has been identified in the screening but not in the *cep* box prediction. We have also used this consensus sequence in our bioinformatics study (Fig. S1). Our predictive method, based on the Chambers’ study, allowed thus to cross data with two others predictive and experimental reports, reinforcing confidence in our results.

### Genes identified in the screening

Our screening allowed us to identify 20 genes corresponding to 17 loci (Table 1). According to the LacZ reporter activity challenge, three genes were strongly downregulated after C_8_-HSL addition (Fig. 5A). These genes are implicated in metabolic functions, such as Bamb_2520, which is the ortholog of *B. cenocepacia cysN*, part of an operon implicated in sulfur metabolism (Iwanicka-Nowicka et al. 2007). This operon is regulated by two LysR-type regulators, CysB and SsuR, but additional QS-regulation has not been reported (Iwanicka-Nowicka et al. 2007). Bamb_3350 (*trpA*) is the last of a six-genes operon implicated in the biosynthesis of tryptophan, which can be then catabolized via the tricarboxylic acids (TCA) cycle, or used as the precursor of many metabolites, such as pyrrolnitrin, 4-hydroxy-2-alkylquinolines (HAQ) produced by *P. aeruginosa*, or their methylated counterparts (HMAQ) discovered in *Burkholderia* (Déziel et al. 2004; Vial et al. 2008; Schmidt et al. 2009). Some of these metabolites are implicated in, or regulated by QS, but the QS-regulation of tryptophan biosynthesis is not established. However, in a quorum-quenching study realized in *Azospirillum lipoferum*, TrpA was identified among the QS-repressed proteins (Boyer et al. 2008), which agrees with our observations. The third mutant included in panel A is trBamb_2378, which is interrupted in the gene coding a spermidine synthase (*speE*)-like protein. Spermidine is a polyamine, implicated in several biological processes, both in eukaryotic and prokaryotic cells (Igarashi and Kashiwagi 2010). As for the other QS-repressed genes, the link with QS is not clear; in *B. pseudomallei*, inhibition of intracellular spermidine synthesis lead to reduced export of AHL via efflux pumps, which suggested that spermidine had an effect on AHL, but the reciprocal was not suggested (Chan and Chua 2010).

It is interesting to note that genes identified in the screening that are QS-repressed or moderately affected are mainly found in the chromosome 1, genes located on chromosome 2 are diversely QS-regulated and genes located on the chromosome 3 are exclusively QS-induced (Table 1). In *B. cenocepacia*, chromosome 1 carries most of essential (“housekeeping”) genes, while the remaining two chromosomes contain much accessory genes implicated in adaptation to niches; the third chromosome has even been described as a virulence plasmid (Holden et al. 2009; Agnoli et al. 2012; Juhas et al. 2012). As discussed above, the link between QS and metabolism is difficult to decipher, as the genes are also regulated by other factors such as the availability of nutriments, whereas QS-regulation of secreted virulence factors is more obvious. The genes moderately affected by C_8_-HSL addition, for which a function is predicted, are implicated in metabolic functions and stress adaptation (Fig. 5C and D; Table 1). Four of these six genes are predicted to be preceded by a putative *cep* box (Table 1; Fig. S1). These genes were at least partially affected by experimental procedures, as two mutants displayed increased β-galactosidase activity if grown in solid rather in liquid medium (Fig. 5D). Another interesting example is trBamb_1141, interrupted in a gene coding a heat shock protein HSP20, which displayed opposite β-galactosidase activities in response to C_8_-HSL addition, according to the temperature of growth (Fig. 6D). In *B. cenocepacia* K56-2, the ortholog of this gene is positively regulated by CepR2, an orphan LuxR transcriptional regulator (Malott et al. 2009). We can thus suggest that the genes that appear moderately affected by C_8_-HSL addition are, additionally to the regulation exerted by C_8_-HSL, controlled by supplementary factors, such as environmental stresses or other regulation circuitry.

Finally, panel B of Figure 5 contains 11 genes strongly induced by C_8_-HSL, such as Bamb_6465, responsible for the cholesterol oxidase activity as mentioned above, or Bamb_5109, located upstream of a large nine-genes operon implicated in the biosynthesis and transport of polysaccharide. The most reactive mutant is trBamb_4726, which is interrupted in *prnA*, the first gene of the operon directing pyrrolnitrin biosynthesis from tryptophan. Pyrrolnitrin is active against a broad spectrum of bacteria and fungi (el-Banna and Winkelmann 1998), and the regulation of its biosynthesis by QS has been demonstrated (Schmidt et al. 2009). The genes Bamb_5911 and Bamb_5925 are implicated in the biosynthesis of enacyloxins, which are antimicrobial compounds produced by *B. ambifaria* and especially active against *B. multivorans* (Mahenthiralingam et al. 2011). Bamb_5925 is important in the biosynthesis, whereas Bamb_5911 is involved in the regulation of the cluster, as it is included in a two-gene operon coding LuxR-type transcriptional regulators (Mahenthiralingam et al. 2011). Indeed, the mutant trBamb_5925 is totally impaired in *B. multivorans* inhibition, while trBamb_5911 is slightly restored when C_8_-HSL is added, revealing that the second LuxR-type transcriptional regulator could partially activate the biosynthesis of enacyloxins (Fig. 6B). Although several elements indicate that the biosynthesis of enacyloxins is QS-regulated (Mahenthiralingam et al. 2011), our data support this assertion. The remaining genes of panel B are almost all orthologs of those implicated in occidiofungins biosynthesis. As discussed above, these antifungal compounds initially identified in *B. contaminans* MS14 have recently been described as hemolytic molecules in *B. vietnamiensis* DBO1 (Lu et al. 2009; Gu et al. 2011; Thomson and Dennis 2012). Another team has identified antifungal molecules named burkholdines in *B. ambifaria* 2.2N, which have structures similar to occidiofungins, demonstrating that the occidiofungin cluster of *B. ambifaria* is expressed (Tawfik et al. 2010). In *B. contaminans*, while the cluster contains two LuxR-type regulators that have C-terminal domains able to bind DNA, neither the autoinducer-binding domain nor the response regulatory domain in *N*-terminal have been identified, suggesting that the signal molecule is from another nature (Gu et al. 2009). Yet, the results of our screening lead us to conclude that the production of occidiofungins is QS-controlled, at least in *B. ambifaria*.

In conclusion, we have confirmed in the clinical *B. ambifaria* HSJ1 strain some genes and phenotypes already known to be QS-regulated in Bcc species, and we have furthermore identified new QS-regulated genes. Predominantly, the production of antifungal/antimicrobial compounds is a very important trait controlled by QS in the HSJ1 WT strain, as our study revealed genes implicated in the biosynthesis of pyrrolnitrin, enacyloxins, and occidiofungins. This arsenal could appear redundant, but each molecule is effective against a different spectrum of microorganisms. Interestingly, a recent study reported that *B. cepacia* strains produced HMAQs displaying antifungal properties (Kilani-Feki et al. 2011). We have previously reported that at least three species of *Burkholderia*, including the *B. ambifaria* strain used in this study, are able to produce HMAQs, and that mutant deficient in the biosynthesis of HMAQs produces increased concentrations of C_8_-HSL (Vial et al. 2008). *Burkholderia ambifaria* HSJ1 expresses thus at least four molecules with antifungal/antimicrobial properties, three of them being QS-regulated; studies are currently underway to determine if biosynthesis of the fourth family of molecules, namely HMAQ, is also regulated by QS.

## Supporting Information

**Data S1.** Supplemental experimental procedures.

**Table S1.** Primers used in this study.

**Table S2.** Conditions used in qRT-PCR experiments

**Figure S1.** Predicted *cep*-box sequences in *Burkholderia* species. The detailed method used to determine the putative *cep*-boxes is described in the explanatory text. All the potential *cep*-boxes found in the AMMD genome (not only those identified in the screening) are also presented.

**Figure S2.** Relative expression of candidate quorum sensing-regulated genes by mRNA quantification. The relative expression of the genes was estimated by quantitative reverse transcription PCR (qRT-PCR) experiments on HSJ1 WT and its *cepI* mutant. The *ndh* gene was used as reference. The results are expressed as relative quantification of gene expression (log_10_ scale) in the *cepI* mutant compared to the WT, normalized to 1. A fold change of two (bottom scale) was chosen as significant threshold. The results are expressed in means ± SD for triplicate assays.

**Figure S3.** Phenotypic confirmation of transposon mutants for antifungal activities. The antifungal activities of HSJ1 WT, its *cepI* mutant and three transposon mutants implicated in biosynthesis of pyrrolnitrin, enacyloxins and occidiofungins, were tested against *Candida albicans*, *Pythium ultimum*, and *Rhizoctonia solani*.
