# Supplementary material for: Identification of quorum sensing-controlled genes in Burkholderia ambifaria
Source: Microbiologyopen. 2013 Feb 5;2(2):226–42. doi: 10.1002/mbo3.67 (PMC3633348; doi:10.1002/mbo3.67)
Supplement: Supplementary file 1 [file mbo30002-0226-SD1.docx]

**Supplemental information**

**Supplemental experimental procedures**

*LC/MS analyses for AHL production detection*

Analyses were performed according to the protocol described in Lépine & Déziel (2011), with the following modifications. Analyses were performed on HPLC (Waters 2795) coupled to a Micromass Quattro Premier XE tandem quadrupole (Micromass Canada, Pointe-Claire, CN) equipped with a Z-spray interface. Samples were injected onto a 4.6 x 150 mm Agilent HP Eclipse XDB-C8 column. Solvent A (water added with 1% acetic acid) and solvent B (acetonitrile added with 1% acetic acid) were used to separate samples using the following gradient: from 0 to 1 min 100% solvent A; from 1 to 5 min linear to 50% solvent A; from 5 to 10 min 50% solvent A; from 10 to 19 min, 100% solvent B; from 19 to 24 min, 100% solvent B; from 24 to 25 min, 100% solvent A; from 25 to 29 min 100% solvent A. Flow rate was 400µL/min to 40 µl/min by the T splitter. The MS parameters were: positive mode; needle voltage 3.0 kV; cone 21V; extractor 5 V; block temperature 120°C and drying gas (nitrogen) 150°C. Analysis mode used multiple reactions monitoring (MRM), using argon as collision gas (0.35mL/min) with a collision energy of 15 V for all the transitions. The following transitions were monitored: HHQ-d4: 148→163; C6-HSL: 200→102; C8-HSL: 228→102 (Lépine and Déziel, 2011). AHL concentrations were calculated from integration of areas, corrected with the response factor (3.166) between C_8_-HSL and HHQ-d4.

**Supplementary tables**

**Table S1: primers used in this study**

| **Purpose** | **Targeted genes** | **Primers' name** | **Primers' sequence** | **Reference** |
| --- | --- | --- | --- | --- |
| **Mutagenesis** | ***cepI*** | **cepIF** | **CGGGGTACCCCGCATGCTCGCGAACGTCAC** | **This study** |
|  |  | **cepIR** | **CTAGTCTAGACTAGCGACGATACCGTCTACGTGTT** | **This study** |
|  | ***cepR*** | **cepRF** | **ctagtctagactaGCTACCAGGCGCAGAACTAC** | **This study** |
|  |  | **cepRR** | **cggggtaccccgTGTTCACGTGGAAGTTGACC** | **This study** |
| **Transposon surroundings** |  | **ISLacOut1F** | **GTTTTCCCAGTCACGACGTT** | **This study** |
|  |  | **ISLacOut1R** | **CGTTTCATCTGTGGTGCAAC** | **This study** |
|  |  | **ISLacOut2F** | **CGTTTACCGCGTTTATCCAC** | **This study** |
|  |  | **ISLacOut2R** | **TAGACTGGGCGGTTTTATGG** | **This study** |
| **qRT-PCR** | **Bamb_1196** | **1196F** | **CTGCGTTACACCGTCTTCG** | **This study** |
|  |  | **1196R** | **AAGTGGTCGCAATAGGCATC** | **This study** |
|  | **Bamb_2378** | **2378 F** | **CGTCACGTTCTCGGAAGAG** | **This study** |
|  |  | **2378 R** | **ATCATCTGCTGCGCGTATTC** | **This study** |
|  | **Bamb_3350** | **3350 F** | **ACCCGTATCCAGCAGACCTT** | **This study** |
|  |  | **3350 R** | **GTGCATGAACTCGACCGTCT** | **This study** |
|  | **Bamb_3836 (*zmpA*)** | **zmpAF** | **ACGGACGGAGATCAGTTCCA** | **This study** |
|  |  | **zmpAR** | **ACGACGTCACCGCCTATCAC** | **This study** |
|  | **Bamb_ 4475 (*zmpB*)** | **zmpB_02F** | **CTACGTGAACCAGACGCTTG** | **This study** |
|  |  | **zmpB_02R** | **TCGACGAGTACGACGAGTTG** | **This study** |
|  | **Bamb_4726** | **4726F** | **TCTTCGACTTCCTCGGGATA** | **This study** |
|  |  | **4726R** | **TTGCCGAACAGATGGTAGAA** | **This study** |
|  | **Bamb_5109** | **5109 F** | **CAGCTGCAGAACGAAGTCAA** | **This study** |
|  |  | **5109 R** | **AGCGGATCAGTTCCTTTCCT** | **This study** |
|  | **Bamb_5911** | **5911F** | **GGACCGCAAGAGAGAACTGA** | **This study** |
|  |  | **5911R** | **CGGTGGTCTTGTTGACGAC** | **This study** |
|  | **Bamb_6465** | **6465_03F** | **GACGCCGCTTTACCAGATT** | **This study** |
|  |  | **6465_03R** | **GATCTCGCCACTCCAGTTCT** | **This study** |
|  | **Bamb_6469** | **6469F** | **GAATGTCGTCGCCGTTCTAT** | **This study** |
|  |  | **6469R** | **CAGGATCCTTTTGCACAGGT** | **This study** |
|  | ***ndh* (reference gene)** | **ndhF** | **GCGATCGGGCTGTACAAGTT** | **Subsin et al. 2007** |
|  |  | **ndhR** | **AGTGGCTCAGCGACTGGAA** | **Subsin et al. 2007** |

**Table S2: conditions used in qRT-PCR experiments**

| **Mix composition** | **Volume (µL)** |
| --- | --- |
| **Master Mix qScript™ One-Step (Quanta)** | **12.5** |
| **Primer F (10 µM)** | **0.75** |
| **Primer R (10 µM)** | **0.75** |
| **DEPC water** | **5.5** |
| **Reverse Transcriptase qScript™ One-Step (Quanta)** | **0.5** |
| **matrix (RNA 20 ng/µL)** | **5** |
| **Total** | **25** |

| **Program** | **T°C** | **Cycles** |
| --- | --- | --- |
| **10 min** | **50°C** |  |
| **5 min** | **95°C** |  |
| **30 sec** | **95°C** | **x 40** |
| **30 sec** | **55 °C** |  |
| **1 min** | **72°C** |  |
| **melt** | **from 55 to 95°C** |  |

**Legends of supplementary figures**

**Figure S1**: **Predicted *cep*-box sequences in *Burkholderia* species.** The detailed method used to determine the putative *cep*-boxes is described in the explanatory text. All the potential *cep*-boxes found in the AMMD genome (not only those identified in the screening) are also presented.

**Figure S2**: **Relative expression of candidate *quorum sensing*-regulated genes by mRNA quantification.** The relative expression of the genes was estimated by quantitative reverse transcription PCR (qRT-PCR) experiments on HSJ1 WT and its *cepI* mutant. The *ndh* gene was used as reference. The results are expressed as relative quantification of gene expression (log_10_ scale) in the *cepI* mutant compared to the WT, normalized to 1. A fold change of two (bottom scale) was chosen as significant threshold. The results are expressed in means ± SD for triplicate assays.

**Figure S3**: **Phenotypic confirmation of transposon mutants for antifungal activities**. The antifungal activities of HSJ1 WT, its *cepI* mutant and three transposon mutants implicated in biosynthesis of pyrrolnitrin, enacyloxins and occidiofungins, were tested against *Candida albicans*, *Pythium ultimum* and *Rhizoctonia solani*.
