# Supplementary material for: Identification of quorum sensing-controlled genes in Burkholderia ambifaria
Source: Microbiologyopen. 2013 Feb 5;2(2):226–42. doi: 10.1002/mbo3.67 (PMC3633348; doi:10.1002/mbo3.67)
Supplement: Supplementary file 2 [file mbo30002-0226-SD2.pdf]

Figure S1

Predicted cepBOX sequences in Burkholderia species

1. Explanatory text
  1.1. Bioinformatics approach (p 1)
  1.2. Results (p 1)

2. Alignments
  2.1. CepBOX for cepI (p 2)
  2.2. Best predictions of cepBOXes (p 4)
  2.3. Potential cepBOXes predicted for genes found in the cepI mutant screen (p 20)

3. Supplementary methods
  3.1. CepBOX consensus used for each search (p 24)
  3.2. RNAmotif descriptors (p 24)

1. Explanatory text

1.1. Bioinformatics approach

To predict instances of cepBOX in Burkholderia species, we relied on previously published reports of cepBOX sequence requirements and on comparative genomics. In other words, we searched for potential cepBOXes (sequences similar to the known consensus) in all available sequences of Burkholderia species (from Burkholderia.com) and compared all of them with their orthologues. The best predictions are those that have a conserved cepBOX among all known orthologues for a given intergenic region (adjacent to the same gene between the (different Burkholderia species) and that have at least one of these predicted cepBOXes that very closely match an ideal cepBOX consensus. In addition, a higher conservation is expected for the cepBOX than for adjacent sequences (although the proximity of the (promoter could interfere with this aspect).

First, we prepared files of all potential cepBOXes (even sequences matching the consensus only loosely) for all intergenic regions of all the sequences available at Burkholderia.com. Then, using Burkholderia ambifaria AMMD as a reference sequence and Perl scripts to automate the process, cepBOXes of intergenic regions were all matched with their corresponding orthologous cepBOXes to look for conservation. Because the polarity of the cepBOX is not known, genes on both sides of the candidate cepBOX were used in order to determine corresponding orthologues, potentially leading to occasional duplicates. The results were then sorted to eliminate cepBOXes with insufficient conservation (based on clustalW) and cepBOXes for which only a small proportion of the known orthologues also had a predicted cepBOX.

1.2 Results:

Cautionary comment:
All the cepBOXes shown here (except for the original cepBOX of cepI) are predictions and should only be regarded as putative cepBOXes. While we think many are likely real cepBOXes, there are probably many false predictions as well. Furthermore, sequences within an alignmentare not necessarily all legitimate instances of the corresponding putative cepBOX, some of them could be spurrious hits.

Alignment description:

The name of each sequence in the alignments corresponds to the two locus (and positions) that surround the intergenic region where the candidate cepBOX is found. (Note that many cepBOXes can be found for a species in a single intergenic region, which is the case for cepI). The candidate cepBOX is shown with adjacent sequences of equivalent size (size vary according to the consensus used to find that cepBOX, Wei's consensus is 24 bases and Chambers' two consensi are 18 bases). The number (5 to 22) adjacent to the sequence illustrates the "cepBOX score". Briefly, 3 to 5 is a close match to Chamber's consensi and 9 to 12 is a close match to Wei's consensus, the smaller, the better (for more details, please see section 3.2 RNAmotif descriptors). Turquoise illustrates well conserved positions, blue illustrates somewhat conserved positions, grey illustrates positions that match two different bases and white illustrates a disagrement with the alignment or no conserved base at that position. Note that the alignment color coding is sometimes "driven" by a group of closely related Burkholderia species or strains (different (B.pseudomallei for instances), without necessarily representing very well the most conserved positions of that particular cepBOX.

The line of text above each alignment corresponds to the gene annotation according to Burkholderia.com. "Either" and "or" are used to indicate the possiblity that the genes regulated by the candidate cepBOX could be in either polarity (which happens frequently). Only the genes for which the polarity is compatible with the cepBOX being close to its promoter are shown, except in the few examples where the candidate cepBOX lies in the regions lying downstream of both genes. These cases are clearly indicated, they are either false predictions or cepBOXes that regulate expression of unannotated transcripts perhaps non-coding RNAs complementary to an adjacent gene. For cepBOXes of genes differently regulated in the cepI mutant screen, the B.ambifaria AMMD locus of the gene found in the screen is specified in parenthesis next to the gene name.

```
2. Alignments
2.1. CepBOX for cepI
confirmed and putative cepBOXes close to cepI

confirmed cepBOX of cepI: the archetypal cepBOX
Bamb_4117,1040062-1040234,Bamb_4118                  gcgccggcgcacgtctac.ctgtaagagttaccagtt.accgcctcctcgggacgc
BamMC406_4582,1689737-1689942,BamMC406_4583          gcgtcggcgcgcgtctac.ctgtaagagttaccagtt.accgcctcctcgggacgc
BCAM1869,2087974-2088183,BCAM1870                    cgtctttacgccgtcacc.ctgtaagagttaccagtt.acaggctcctcgtgccgc
Bcen_3641,767661-767870,Bcen_3642                    cgtctttacgtcgccacc.ctgtaagagttaccagtt.acaggctcctcgtgccgc
Bcen2424_4725,1711797-1712006,Bcen2424_4726          cgtctttacgtcgccacc.ctgtaagagttaccagtt.acaggctcctcgtgccgc
Bcenmc03_5574,2718276-2718485,Bcenmc03_5575          cgtctttacgtcgccacc.ctgtaagagttaccagtt.acaggctcctcgtgccgc
Bcep1808_5261,2192834-2193042,Bcep1808_5262          taccggtaagccgccacc.ctgtaagagttaccagtt.acagcctcctcgggccgc
Bcep18194_B1050,1176998-1177211,Bcep18194_B1051      cgtctttccggcgtcacc.ctgtaagagttaccagtt.acagcccctcgtgctgc
BMA10229_0608,644790-645013,BMA10229_0609            agccccgctttcccgacc.ctgtaagggttaacagtt.acctgtcctcggagcgcg
BMA10247_A0960,910153-910376,BMA10247_A0961          agccccgctttcccgacc.ctgtaagggttaacagtt.acctgtcctcggagcgcg
BMAA1346,1449594-1449817,BMAA1347                    agccccgctttcccgacc.ctgtaagggttaacagtt.acctgtcctcggagcgcg
BMASAVP1_0330,348863-349086,BMASAVP1_0331            agccccgctttcccgacc.ctgtaagggttaacagtt.acctgtcctcggagcgcg
Bmul_3969,944039-944209,Bmul_3970                    cgtttccgcgccgcaata.ctgtaagagttaccagtt.accgccgcctcgggacgc
BPSS0885,1178132-1178355,BPSS0886                    agccccgctttcccgacc.ctgtaagggttaacagtt.acctgtcctcggagcgcg

other potential cepBOXes in the intergenic region upstream of cepI
Bamb_4117,1040062-1040234,Bamb_4118                  aagactggcgctctttat.aagcaaaagccgcccgta.tttcaatcccgccgatca
Bcen_3641,767661-767870,Bcen_3642                    cagactggcgctctttat.aaataaaagccgcccggt.tttcaatcccgtcgatca
Bcen2424_4725,1711797-1712006,Bcen2424_4726          cagactggcgctctttat.aaataaaagccgcccggt.tttcaatcccgtcgatca
Bcenmc03_5574,2718276-2718485,Bcenmc03_5575          cagactggcgctctttat.aaataaaagccgcccggt.tttcaatcccgtcgatca
BMA10229_0608,644790-645013,BMA10229_0609            agccccgctttcccgacc.ctgtaagggttaacagtt.acctgtcctcggagcgcg
BMA10247_A0960,910153-910376,BMA10247_A0961          agccccgctttcccgacc.ctgtaagggttaacagtt.acctgtcctcggagcgcg
BMAA1346,1449594-1449817,BMAA1347                    agccccgctttcccgacc.ctgtaagggttaacagtt.acctgtcctcggagcgcg
BMASAVP1_0330,348863-349086,BMASAVP1_0331            agccccgctttcccgacc.ctgtaagggttaacagtt.acctgtcctcggagcgcg
BURPS668_A1293,1222515-1222738,BURPS668_A1294        agccccgctttcccgacc.ctgtaagggttaacagtt.acctgtcctcggagcgcg

Bamb_4117,1040062-1040234,Bamb_4118                  atcccgccgatcaagaaaccgtta.ccaccggtcaaatcgcgccggcgc.acgtctacctgtaagagttaccag
BamMC406_4582,1689737-1689942,BamMC406_4583          tacagcgcgcgtcccgaggaggcg.gtaactggtaactcttacaggtag.acgcgcgccgacgcgatttgaccg
BCAM1869,2087974-2088183,BCAM1870                    gcggttttcaatcccgttgatcaa.gaaaccgttaccacgtcccgaatg.gcgtctttacgccgtcaccctgta
Bcenmc03_5574,2718276-2718485,Bcenmc03_5575          tcccgaatggcgtctttacgtcgc.caccctgtaagagttaccagttac.aggctcctcgtgccgcgcgctgta
Bcep1808_5261,2192834-2193042,Bcep1808_5262          atcccgccgatcaagaaaccgtta.ccaccggtcgaatgataccggtaa.gccgccaccctgtaagagttacca
Bcep18194_B1050,1176998-1177211,Bcep18194_B1051      acgggattgaaaaccgtggcgctt.ttgcttataaagagcgccagcatc.gcgtaaattgtcagaggaataggt
BMA10229_0608,644790-645013,BMA10229_0609            ccggcggaacccgccccgcccccg.ccaccagccccgctttcccgaccc.tgtaagggttaacagttacctgtc
BMA10247_A0960,910153-910376,BMA10247_A0961          ccggcggaacccgccccgcccccg.ccaccagccccgctttcccgaccc.tgtaagggttaacagttacctgtc
BMAA1346,1449594-1449817,BMAA1347                    ccggcggaacccgccccgcccccg.ccaccagccccgctttcccgaccc.tgtaagggttaacagttacctgtc
BMASAVP1_0330,348863-349086,BMASAVP1_0331            ccggcggaacccgccccgcccccg.ccaccagccccgctttcccgaccc.tgtaagggttaacagttacctgtc
BTH_II1511,1780307-1780523,BTH_II1512                ttacatcgcgcgctccgaggacag.gtaactgttaacccttacagggtc.ggaaagcgaggcggccagggggag
BURPS1106A_A1221,1159833-1160056,BURPS1106A_A1222    ccggcggaacccgccccgcccccg.ccaccagccccgctttcccgaccc.tgtaagggttaacagttacctgtc
BURPS1710b_A2483,2999372-2999595,BURPS1710b_A2484    ccggcggaacccgccccgcccccg.ccaccagccccgctttcccgaccc.tgtaagggttaacagttacctgtc
BURPS668_A1293,1222515-1222738,BURPS668_A1294        ccggcggaacccgccccgcccccg.ccaccagccccgctttcccgaccc.tgtaagggttaacagttacctgtc

Bamb_4117,1040062-1040234,Bamb_4118                  ttaccaccggtcaaatcgcgccgg.cgcacgtctacctgtaagagttac.cagttaccgcctcctcgggacgcg
BamMC406_4582,1689737-1689942,BamMC406_4583          ttaccaccggtcaaatcgcgtcgg.cgcgcgtctacctgtaagagttac.cagttaccgcctcctcgggacgcg
BCAM1869,2087974-2088183,BCAM1870                    tcccgaatggcgtctttacgccgt.caccctgtaagagttaccagttac.aggctcctcgtgccgcgcgctgta
Bcen_3641,767661-767870,Bcen_3642                    tcccgaatggcgtctttacgtcgc.caccctgtaagagttaccagttac.aggctcctcgtgccgcgcgctgta
Bcen2424_4725,1711797-1712006,Bcen2424_4726          tcccgaatggcgtctttacgtcgc.caccctgtaagagttaccagttac.aggctcctcgtgccgcgcgctgta
Bcenmc03_5574,2718276-2718485,Bcenmc03_5575          tcccgaatggcgtctttacgtcgc.caccctgtaagagttaccagttac.aggctcctcgtgccgcgcgctgta
Bcep1808_5261,2192834-2193042,Bcep1808_5262          ggtcgaatgataccggtaagccgc.caccctgtaagagttaccagttac.agcctcctcgggccgcgcgatgta
Bcep18194_B1050,1176998-1177211,Bcep18194_B1051      tcctaaacgacgtctttccggcgt.caccctgtaagagttaccagttac.agcccctcgtgctgcgcgatgta
BMA10229_0608,644790-645013,BMA10229_0609            tggaatgggcgcattacatcgcgc.gctccgaggacaggtaactgttaa.cccttacagggtcgggaaagcggg
BMA10247_A0960,910153-910376,BMA10247_A0961          tggaatgggcgcattacatcgcgc.gctccgaggacaggtaactgttaa.cccttacagggtcgggaaagcggg
BMAA1346,1449594-1449817,BMAA1347                    tggaatgggcgcattacatcgcgc.gctccgaggacaggtaactgttaa.cccttacagggtcgggaaagcggg
BMASAVP1_0330,348863-349086,BMASAVP1_0331            tggaatgggcgcattacatcgcgc.gctccgaggacaggtaactgttaa.cccttacagggtcgggaaagcggg
Bmul_3969,944039-944209,Bmul_3970                    aagcgcgcgcgttttcaatcccgc.cccacgggaaaccgttaccgcgtc.gaaattggcgtttccgcgccgcaa
BPSS0885,1178132-1178355,BPSS0886                    tggaatgggcgcattacatcgcgc.gctccgaggacaggtaactgttaa.cccttacagggtcgggaaagcggg
BTH_II1511,1780307-1780523,BTH_II1512                tggaatgggcgcattacatcgcgc.gctccgaggacaggtaactgttaa.cccttacagggtcggaaagcgagg
BURPS1106A_A1221,1159833-1160056,BURPS1106A_A1222    tggaatgggcgcattacatcgcgc.gctccgaggacaggtaactgttaa.cccttacagggtcgggaaagcggg
BURPS1710b_A2483,2999372-2999595,BURPS1710b_A2484    tggaatgggcgcattacatcgcgc.gctccgaggacaggtaactgttaa.cccttacagggtcgggaaagcggg
BURPS668_A1293,1222515-1222738,BURPS668_A1294        tggaatgggcgcattacatcgcgc.gctccgaggacaggtaactgttaa.cccttacagggtcgggaaagcggg

Bamb_4117,1040062-1040234,Bamb_4118                  cggtcaaatcgcgccggcgcacgt.ctacctgtaagagttaccagttac.cgcctcctcgggacgcgcgctgta
BamMC406_4582,1689737-1689942,BamMC406_4583          cggtcaaatcgcgtcggcgcgcgt.ctacctgtaagagttaccagttac.cgcctcctcgggacgcgcgctgta
BCAM1869,2087974-2088183,BCAM1870                    tcccgaatggcgtctttacgccgt.caccctgtaagagttaccagttac.aggctcctcgtgccgcgcgctgta
Bcen_3641,767661-767870,Bcen_3642                    tcccgaatggcgtctttacgtcgc.caccctgtaagagttaccagttac.aggctcctcgtgccgcgcgctgta
Bcen2424_4725,1711797-1712006,Bcen2424_4726          tcccgaatggcgtctttacgtcgc.caccctgtaagagttaccagttac.aggctcctcgtgccgcgcgctgta
Bcenmc03_5574,2718276-2718485,Bcenmc03_5575          tcccgaatggcgtctttacgtcgc.caccctgtaagagttaccagttac.aggctcctcgtgccgcgcgctgta
Bcep1808_5261,2192834-2193042,Bcep1808_5262          ggtcgaatgataccggtaagccgc.caccctgtaagagttaccagttac.agcctcctcgggccgcgcgatgta
Bcep18194_B1050,1176998-1177211,Bcep18194_B1051      tcctaaacgacgtctttccggcgt.caccctgtaagagttaccagttac.agcccctcgtgctgcgcgatgta
BMA10229_0608,644790-645013,BMA10229_0609            ccccgccaccagccccgctttccc.gaccctgtaagggttaacagttac.ctgtcctcggagcgcgcgatgtaa
BMA10247_A0960,910153-910376,BMA10247_A0961          ccccgccaccagccccgctttccc.gaccctgtaagggttaacagttac.ctgtcctcggagcgcgcgatgtaa
BMAA1346,1449594-1449817,BMAA1347                    ccccgccaccagccccgctttccc.gaccctgtaagggttaacagttac.ctgtcctcggagcgcgcgatgtaa
Bmul_3969,944039-944209,Bmul_3970                    ttgcggcgcggaaacgccaatttc.gacgcggtaacggtttcccgtggg.gcgggattgaaaacgcgcgcgctt
BPSS0885,1178132-1178355,BPSS0886                    ccccgccaccagccccgctttccc.gaccctgtaagggttaacagttac.ctgtcctcggagcgcgcgatgtaa
BTH_II1511,1780307-1780523,BTH_II1512                ctcccctggccgcctcgctttccc.gaccctgtaagggttaacagttac.ctgtcctcggagcgcgcgatgtaa
BURPS1106A_A1221,1159833-1160056,BURPS1106A_A1222    ccccgccaccagccccgctttccc.gaccctgtaagggttaacagttac.ctgtcctcggagcgcgcgatgtaa
BURPS1710b_A2483,2999372-2999595,BURPS1710b_A2484    ccccgccaccagccccgctttccc.gaccctgtaagggttaacagttac.ctgtcctcggagcgcgcgatgtaa

Bamb_4117,1040062-1040234,Bamb_4118                  ttacaggtagacgtgcgc.cggcgcgatttgaccggt.ggtaacggtttcttgatc
Bcen_3641,767661-767870,Bcen_3642                    cagactggcgctctttat.aaataaaagccgcccggt.tttcaatcccgtcgatca
Bcenmc03_5575,2718930-2719002,Bcenmc03_5576          ttctccgacaacgtcacg.ctgtcatacttgtcaggt.tccagcacccgttcgagg
BMA10229_0607,644273-644345,BMA10229_0608            ttctccagcagcttcacg.ctgtcatacttgctaggt.ttcagcacgcgcgcgggc
BTH_II1510,1779790-1779862,BTH_II1511                ttctccagcagcttcacg.ctgtcatacttgctaggt.ttcagcacgcgcgcggac

Bamb_4117,1040062-1040234,Bamb_4118                  gtcccgaggaggcggtaa.ctggtaactcttacaggt.agacgtgcgccggcgcga
BamMC406_4582,1689737-1689942,BamMC406_4583          gtcccgaggaggcggtaa.ctggtaactcttacaggt.agacgcgcgccgacgcga
BCAM1869,2087974-2088183,BCAM1870                    cgtctttacgccgtcacc.ctgtaagagttaccagtt.acaggctcctcgtgccgc
Bcen_3641,767661-767870,Bcen_3642                    cgtctttacgtcgccacc.ctgtaagagttaccagtt.acaggctcctcgtgccgc
Bcen2424_4725,1711797-1712006,Bcen2424_4726          cgtctttacgtcgccacc.ctgtaagagttaccagtt.acaggctcctcgtgccgc
Bcep1808_5261,2192834-2193042,Bcep1808_5262          tacagggtggcggcttac.cggtatcattcgaccggt.ggtaacggtttcttgatc
BMAA1346,1449594-1449817,BMAA1347                    cgctccgaggacaggtaa.ctgttaacccttacaggg.tcgggaaagcggggctgg
Bmul_3969,944039-944209,Bmul_3970                    cgtttccgcgccgcaata.ctgtaagagttaccagtt.accgccgcctcgggacgc
BPSS0885,1178132-1178355,BPSS0886                    cgctccgaggacaggtaa.ctgttaacccttacaggg.tcgggaaagcggggctgg
BTH_II1511,1780307-1780523,BTH_II1512                cgctccgaggacaggtaa.ctgttaacccttacaggg.tcggaaagcgaggcggcc
BURPS1106A_A1221,1159833-1160056,BURPS1106A_A1222    cgctccgaggacaggtaa.ctgttaacccttacaggg.tcgggaaagcggggctgg
BURPS1710b_A2483,2999372-2999595,BURPS1710b_A2484    cgctccgaggacaggtaa.ctgttaacccttacaggg.tcgggaaagcggggctgg
```

confirmed and putative cepBOXes close to cepI

cepBOXes between cepR an a hypothetical protein (adjacent to cepI)

```
Bamb_4116,1039545-1039617,Bamb_4117                     ttctccgacaacgtcacg.ctgtcatacctgtcaggt.gccagcgccggttcgggg
BamMC406_4581,1689220-1689292,BamMC406_4582             ttctccgacaacgtcacg.ctgtcatacctgtcaggt.gccagcgccggttcgggg
BCAM1868,2087457-2087529,BCAM1869                       ttctccgacaacgtcacg.ctgtcatacttgtcaggt.ttcagtacccgttcgagg
Bcen_3642,768315-768387,Bcen_3643                       ttctccgacaacgtcacg.ctgtcatacttgtcaggt.tccagcacccgttcgagg
Bcen2424_4724,1711280-1711352,Bcen2424_4725             ttctccgacaacgtcacg.ctgtcatacttgtcaggt.tccagcacccgttcgagg
Bcenmc03_5575,2718930-2719002,Bcenmc03_5576             ttctccgacaacgtcacg.ctgtcatacttgtcaggt.tccagcacccgttcgagg
Bcep1808_5260,2192317-2192389,Bcep1808_5261             ttctccgacaacgtcacg.ctgtcatacctgtcaggt.gccagcaccggttggggg
Bcep18194_B1051,1177656-1177728,Bcep18194_B1052         ttctccgacaacgtcacg.ctgtcatacttgtcaggt.tccagcacccgttcgagg
BMA10229_0607,644273-644345,BMA10229_0608               ttctccagcagcttcacg.ctgtcatacttgctaggt.ttcagcacgcgcgcgggc
BMA10247_A0961,910821-910893,BMA10247_A0962             ttctccagcagcttcacg.ctgtcatacttgctaggt.ttcagcacgcgcgcgggc
BMAA1345,1449077-1449149,BMAA1346                       ttctccagcagcttcacg.ctgtcatacttgctaggt.ttcagcacgcgcgcgggc
BMASAVP1_0329,348346-348418,BMASAVP1_0330               ttctccagcagcttcacg.ctgtcatacttgctaggt.ttcagcacgcgcgcgggc
Bmul_3970,944654-944727,Bmul_3971                       ttctccgacaacgtcacg.ctgtcatacttgtcaggt.tccagcaccgatcggcgc
BPSS0886,1178800-1178872,BPSS0887                       ttctccagcagcttcacg.ctgtcatacttgctaggt.ttcagcacgcgcgcgggc
BTH_II1510,1779790-1779862,BTH_II1511                   ttctccagcagcttcacg.ctgtcatacttgctaggt.ttcagcacgcgcgcggac
BURPS1106A_A1222,1160501-1160573,BURPS1106A_A1223       ttctccagcagcttcacg.ctgtcatacttgctaggt.ttcagcacgcgcgcgggc
BURPS1710b_A2484,3000040-3000112,BURPS1710b_A2485       ttctccagcagcttcacg.ctgtcatacttgctaggt.ttcagcacgcgcgcgggc
BURPS668_A1294,1223183-1223255,BURPS668_A1295           ttctccagcagcttcacg.ctgtcatacttgctaggt.ttcagcacgcgcgcgggc


Bamb_4116,1039545-1039617,Bamb_4117                     ccgaaccggcgctggcac.ctgacaggtatgacagcg.tgacgttgtcggagaaat
BamMC406_4581,1689220-1689292,BamMC406_4582             ccgaaccggcgctggcac.ctgacaggtatgacagcg.tgacgttgtcggagaaat
BCAM1868,2087457-2087529,BCAM1869                       tcgaacgggtactgaaac.ctgacaagtatgacagcg.tgacgttgtcggagaaag
Bcen_3642,768315-768387,Bcen_3643                       tcgaacgggtgctggaac.ctgacaagtatgacagcg.tgacgttgtcggagaacg
Bcen2424_4724,1711280-1711352,Bcen2424_4725             tcgaacgggtgctggaac.ctgacaagtatgacagcg.tgacgttgtcggagaacg
Bcenmc03_5575,2718930-2719002,Bcenmc03_5576             tcgaacgggtgctggaac.ctgacaagtatgacagcg.tgacgttgtcggagaacg
Bcep1808_5260,2192317-2192389,Bcep1808_5261             cccaaccggtgctggcac.ctgacaggtatgacagcg.tgacgttgtcggagaaag
Bcep18194_B1051,1177656-1177728,Bcep18194_B1052         tcgaacgggtgctggaac.ctgacaagtatgacagcg.tgacgttgtcggagaatg
BMA10247_A0961,910821-910893,BMA10247_A0962             ttctccagcagcttcacg.ctgtcatacttgctaggt.ttcagcacgcgcgcgggc
BMASAVP1_0329,348346-348418,BMASAVP1_0330               ttctccagcagcttcacg.ctgtcatacttgctaggt.ttcagcacgcgcgcgggc
Bmul_3970,944654-944727,Bmul_3971                       gccgatcggtgctggaac.ctgacaagtatgacagcg.tgacgttgtcggagaaag
BTH_II1510,1779790-1779862,BTH_II1511                   ttctccagcagcttcacg.ctgtcatacttgctaggt.ttcagcacgcgcgcggac
BURPS1710b_A2484,3000040-3000112,BURPS1710b_A2485       ttctccagcagcttcacg.ctgtcatacttgctaggt.ttcagcacgcgcgcgggc

BamMC406_4581,1689220-1689292,BamMC406_4582             ttctccgacaacgtcacg.ctgtcatacctgtcaggt.gccagcgccggttcgggg
BCAM1868,2087457-2087529,BCAM1869                       ttctccgacaacgtcacg.ctgtcatacttgtcaggt.ttcagtacccgttcgagg
Bcen_3642,768315-768387,Bcen_3643                       ttctccgacaacgtcacg.ctgtcatacttgtcaggt.tccagcacccgttcgagg
Bcen2424_4724,1711280-1711352,Bcen2424_4725             ttctccgacaacgtcacg.ctgtcatacttgtcaggt.tccagcacccgttcgagg
Bcenmc03_5575,2718930-2719002,Bcenmc03_5576             ttctccgacaacgtcacg.ctgtcatacttgtcaggt.tccagcacccgttcgagg
Bcep1808_5260,2192317-2192389,Bcep1808_5261             ttctccgacaacgtcacg.ctgtcatacctgtcaggt.gccagcaccggttggggg
BMA10247_A0961,910821-910893,BMA10247_A0962             ttctccagcagcttcacg.ctgtcatacttgctaggt.ttcagcacgcgcgcgggc
BMAA1345,1449077-1449149,BMAA1346                       ttctccagcagcttcacg.ctgtcatacttgctaggt.ttcagcacgcgcgcgggc
BMASAVP1_0329,348346-348418,BMASAVP1_0330               ttctccagcagcttcacg.ctgtcatacttgctaggt.ttcagcacgcgcgcgggc
BPSS0886,1178800-1178872,BPSS0887                       ttctccagcagcttcacg.ctgtcatacttgctaggt.ttcagcacgcgcgcgggc
BTH_II1510,1779790-1779862,BTH_II1511                   ttctccagcagcttcacg.ctgtcatacttgctaggt.ttcagcacgcgcgcggac
Bmul_3970,944654-944727,Bmul_3971                       ttctccgacaacgtcacg.ctgtcatacttgtcaggt.tccagcaccgatcggcgc
BURPS1106A_A1222,1160501-1160573,BURPS1106A_A1223       ttctccagcagcttcacg.ctgtcatacttgctaggt.ttcagcacgcgcgcgggc

BamMC406_4581,1689220-1689292,BamMC406_4582             ttctccgacaacgtcacg.ctgtcatacctgtcaggt.gccagcgccggttcgggg
BCAM1868,2087457-2087529,BCAM1869                       ttctccgacaacgtcacg.ctgtcatacttgtcaggt.ttcagtacccgttcgagg
Bcen_3642,768315-768387,Bcen_3643                       ttctccgacaacgtcacg.ctgtcatacttgtcaggt.tccagcacccgttcgagg
Bcen2424_4724,1711280-1711352,Bcen2424_4725             ttctccgacaacgtcacg.ctgtcatacttgtcaggt.tccagcacccgttcgagg
Bcenmc03_5575,2718930-2719002,Bcenmc03_5576             ttctccgacaacgtcacg.ctgtcatacttgtcaggt.tccagcacccgttcgagg
Bcep1808_5260,2192317-2192389,Bcep1808_5261             ttctccgacaacgtcacg.ctgtcatacctgtcaggt.gccagcaccggttggggg
Bcep18194_B1051,1177656-1177728,Bcep18194_B1052         ttctccgacaacgtcacg.ctgtcatacttgtcaggt.tccagcacccgttcgagg
BMA10229_0607,644273-644345,BMA10229_0608               ttctccagcagcttcacg.ctgtcatacttgctaggt.ttcagcacgcgcgcgggc
BMA10247_A0961,910821-910893,BMA10247_A0962             ttctccagcagcttcacg.ctgtcatacttgctaggt.ttcagcacgcgcgcgggc
BMAA1345,1449077-1449149,BMAA1346                       ttctccagcagcttcacg.ctgtcatacttgctaggt.ttcagcacgcgcgcgggc
BMASAVP1_0329,348346-348418,BMASAVP1_0330               ttctccagcagcttcacg.ctgtcatacttgctaggt.ttcagcacgcgcgcgggc
Bmul_3970,944654-944727,Bmul_3971                       ttctccgacaacgtcacg.ctgtcatacttgtcaggt.tccagcaccgatcggcgc
BPSS0886,1178800-1178872,BPSS0887                       ttctccagcagcttcacg.ctgtcatacttgctaggt.ttcagcacgcgcgcgggc
```

end of page 3

2.2. Best predictions of cepBOXes

highest scoring predicted cepBOXes in Burkholderia genomes

putative cepBOX between a "histone family protein DNA-binding protein" and "cobalamin synthesis protein, P47K"
Note that this putative cepBOX would be downstream of either of those genes. Thus, if it is used with a promoter, it would be for an unknown transcript (complementary to either of those genes?).

```
Bamb_0054,65377-65511,Bamb_0055                        10  gccacagtccgcacacct.cttgcaagatttaacggt..tagctttcgcgcgccgtt
BCAL3529,3867113-3867241,BCAL3530                       5  ccacagttcgcgccgct..cttgcaaaagttaacggtt.acttttacaagccgttac
Bcen_0005,5893-6020,Bcen_0006                           5  ccacagttcgcgccgct..cttgcaaaagttaacggtt.attttacaagccgttacc
Bcen2424_0064,66435-66562,Bcen2424_0065                 5  ccacagttcgcgccgct..cttgcaaaagttaacggtt.attttacaagccgttacc
Bcenmc03_0084,97982-98369,Bcenmc03_0085                10  tattgggtttgcgctgcg.ctatcatagcaacgccgt..accgcggggtggccgccg
Bcep1808_0073,82537-82676,Bcep1808_0074                10  accacagtgtgcgccgcg.cttgcaagatttaacgtt..gtgtcggccgcttgccag
Bcep18194_A3247,79803-80190,Bcep18194_A3248            10  tattgggtttgcgctgcg.ctatcatagcaacgccgt..accgcggggtggccaccg
Bmul_0065,70035-70149,Bmul_0066                        10  atcgacgaaccgtaaaat.cttgtaaaggtgtgacgg..actgtggcaaaaatgccg
Bxe_A4505,4849711-4850202,Bxe_A4504                     9  cccaatcaggacaagggt.ttcgaagaattacgcggt..tgcgcggcggatattgca
```

putative cepBOX preceding the gene "aquaporin Z" or "histone family protein nucleoid-structuring protein H-NS"

```
Bamb_0161,194323-194620,Bamb_0162                       5  gttcaagagggtcaacga.ttgttaaatttgaatgct.ggcgaatggcctttatta
BamMC406_0174,210177-210474,BamMC406_0175               5  gttcaagagggtcaacga.ttgttaaatttgaatgct.ggcgaatggcctttatta
Bmul_0157,178805-179093,Bmul_0158                       8  ccgcatcgtggtcaacga.ttgttaaatttgagcagc.tgacaaatcgctttatta
Bphy_2980,3339404-3339703,Bphy_2981                    10  tcagagcggaccgcgcat.tagccaaatcagaaagtg.gcgaattcagctagaaca
Bxe_A0112,4724308-4724596,Bxe_A0111                    10  aatcggtattgttgtcca.ttgcgaattttataatt.acagttagtctgcgagcg
```

putative cepBOX preceding a hypothetical protein

```
Bamb_0213,249145-249372,Bamb_0214                        5  gactcgtgtccgttgaaa.gttgcagagctgtcaggt.gaggagatgccgataagg
BamMC406_0221,256628-256953,BamMC406_0222                6  gactcgtgtccgttgaaa.attgcagagctgtcaggt.gaggagatgccgataagg
Bcenmc03_3462,331473-332063,Bcenmc03_3463               10  gttttaatttttcccatg.atctcatggcaatgagtt.tgctggtgttcttcgagc
Bcep1808_7688,14426-15279,Bcep1808_7689                 10  tgtgccatcccgcttcac.ggggtaaattcgtcagac.ggaaacagcggagcgtcc
```

putative cepBOX preceding a hypothetical protein

```
Bamb_0378,424062-424165,Bamb_0379                       9  tgacgcgcgaatgtcaat.taacaagagttaacaatt.ggcgagttaattcgggca
BamMC406_0399,448680-448783,BamMC406_0400               9  tgacgcgcgaatgtcaat.taacaagagttaacaatt.ggcgagttaattcgggca
Bcen2424_0464,516912-517013,Bcen2424_0465               8  tgacgcacggatgtcaat.taacaagagttaacactt.ggcgatttaattcgggca
Bcenmc03_0442,481194-481295,Bcenmc03_0443               8  tgacgcacggatgtcaat.taacaagagttaacactt.ggcgatttaattcgggca
Bcep1808_0457,493762-493863,Bcep1808_0458              10  tgacgcacgaatgtcaat.taacaagagttaacaaat.tggcgattaattcgggca
Bmul_2932,3208199-3208300,Bmul_2933                    10  tgacgcgcgaatgtcaat.taacaagagttaacaaat.cgcgatttaattcgggca
BPSL3108,3713951-3714324,BPSL3109                      10  cgaaatcgaattccgttg.cggcaataattgcaatgg.gagcggtacacggcgatc
BTH_I2965,3413093-3413201,BTH_I2966                     9  tatccccggcgcgggcgt.tttaaatcgttgagaatt.cgtgatttttgctttgcta
BURPS1106A_3662,3564754-3564875,BURPS1106A_3663         5  gcgtccgccccttgctcc.ctgtcaaaactatcagtt.actttgccgatttggcgg
BURPS1710b_3646,3972049-3972421,BURPS1710b_3647        10  cgaaatcgaattccgttg.cggcaataattgcaatgg.gagcggtacacggcgatc
BURPS668_3637,3563324-3563434,BURPS668_3638             5  gcgtccgccccttgctcc.ctgtcaaaactatcagtt.actttgccgatttggcgg
```

end of page 4

putative cepBOX preceding a hypothetical protein or a "TPR repeat-containing protein"

```
Bamb_0379,424775-425149,Bamb_0380                    15  tatttccggcgtgccgcctttaac.tccccctgtcaaaactaccgggctt.tccgcgctctccgcgcctgtccgg
BamMC406_0400,449393-449768,BamMC406_0401            15  tatttccggcgtgccgcctttaac.tccccctgtcaaaactaccgggcct.tccgcgctctccgcgcctgtccgg
BCAL0339,365846-366231,BCAL0340                      15  cgtttttccggcgtgccgcctttt.atccctgtcaaaactgccgggata.ttgccgcgttcgctgcgtatcccc
Bcenmc03_0443,481908-482279,Bcenmc03_0444            22  actggtcgcgcgacgcgccgcccg.gacggcggaaaaatttccggacg.ctcgtccgacgcgggcgtgcttaa
Bcep18194_A3557,423772-424145,Bcep18194_A3558        14  cggtttttccggcgggctgcctttt.atccctgtcaaatctgccgggtat.ttccgcgttcgctgcgtatccccg
Bmul_2931,3207212-3207586,Bmul_2932                  13  gtatttccggcgtgccgcctttttg.ctccctgtcaaaactgccgggcat.ttcgcgtatcgacgcgatgcgcgg
BPSL3108,3713951-3714324,BPSL3109                    10  ttttcagggcgcgtccgcccccttg.ctccctgtcaaaactatcagttac.tttgccgatttggcggcgaaaaaa
BURPS1106A_3662,3564754-3564875,BURPS1106A_3663      10  ttttcagggcgcgtccgcccccttg.ctccctgtcaaaactatcagttac.tttgccgatttggcggcgaaaaaa
BURPS1710b_3646,3972049-3972421,BURPS1710b_3647      10  ttttcagggcgcgtccgcccccttg.ctccctgtcaaaactatcagttac.tttgccgatttggcggcgaaaaaa
BURPS668_3637,3563324-3563434,BURPS668_3638          10  ttttcagggcgcgtccgcccccttg.ctccctgtcaaaactatcagttac.tttgccgatttggcggcgaaaaaa
```

putative cepBOX preceding a hypothetical protein or a "TPR repeat-containing protein"

```
Bamb_0379,424775-425149,Bamb_0380                    18  actaccgggctttccgcgctctcc.gcgcctgtccggcttatctggtgt.tcaactattaagcaaacgtgcggc
BamMC406_0400,449393-449768,BamMC406_0401            18  actaccgggccttccgcgctctcc.gcgcctgtccggcttatctggtgt.tcaactattaagcaaacgtgcggc
BCAL0339,365846-366231,BCAL0340                      17  ctggttgcacgacgcgccgcccgg.atggcggaaaaatttccggatgc.tcgcctgacgcgggcgtgcttaat
Bmul_2931,3207212-3207586,Bmul_2932                  13  gtatttccggcgtgccgcctttttg.ctccctgtcaaaactgccgggcat.ttcgcgtatcgacgcgatgcgcgg
BPSL3108,3713951-3714324,BPSL3109                    10  ttttcagggcgcgtccgcccccttg.ctccctgtcaaaactatcagttac.tttgccgatttggcggcgaaaaaa
BURPS1106A_3662,3564754-3564875,BURPS1106A_3663      10  ttttcagggcgcgtccgcccccttg.ctccctgtcaaaactatcagttac.tttgccgatttggcggcgaaaaaa
BURPS1710b_3646,3972049-3972421,BURPS1710b_3647      10  ttttcagggcgcgtccgcccccttg.ctccctgtcaaaactatcagttac.tttgccgatttggcggcgaaaaaa
BURPS668_3637,3563324-3563434,BURPS668_3638          11  ttttcagggcgcgtccgcccccttg.ctccctgtcaaaactatcagttac.tttgccgatttggcggcgaaaaaa
```

putative cepBOX preceding a hypothetical protein or a "TPR repeat-containing protein"

```
Bamb_0379,424775-425149,Bamb_0380                    10  tttccgcgctctccgcgc.ctgtccggcttatctggt.gttcaactattaagcaaa
BamMC406_0400,449393-449768,BamMC406_0401            10  cttccgcgctctccgcgc.ctgtccggcttatctggt.gttcaactattaagcaaa
Bcenmc03_0443,481908-482279,Bcenmc03_0444            10  cggcgaacgcggcaatac.ccggcagttttgacaggg.ataaaggcggcacgccgg
Bcep18194_A3557,423772-424145,Bcep18194_A3558         9  ttttctttcggccggaat.gggcaagttttactggtt.tcgcgacgtgccgcccgg
Bmul_2931,3207212-3207586,Bmul_2932                   7  ggccgctctgtcgcgacg.ctggcaggcttaatagtt.gaacgagggaccggccgc
BPSL3108,3713951-3714324,BPSL3109                     5  gcgtccgccccttgctcc.ctgtcaaaactatcagtt.actttgccgatttggcgg
BURPS1106A_3662,3564754-3564875,BURPS1106A_3663       5  gcgtccgccccttgctcc.ctgtcaaaactatcagtt.actttgccgatttggcgg
BURPS1710b_3646,3972049-3972421,BURPS1710b_3647       5  gcgtccgccccttgctcc.ctgtcaaaactatcagtt.actttgccgatttggcgg
BURPS668_3637,3563324-3563434,BURPS668_3638           5  gcgtccgccccttgctcc.ctgtcaaaactatcagtt.actttgccgatttggcgg
```

putative cepBOX preceding a hypothetical protein or a "TPR repeat-containing protein" (either could possibly be associated with type VI secretion systems)

```
Bamb_0379,424775-425149,Bamb_0380                     9  gtgccgcctttaactccc.ctgtcaaaactaccgggc.tttccgcgctctccgcgc
BamMC406_0400,449393-449768,BamMC406_0401             9  gtgccgcctttaactccc.ctgtcaaaactaccgggc.cttccgcgctctccgcgc
BCAL0339,365846-366231,BCAL0340                       8  ggcgtgccgcctttatcc.ctgtcaaaactgccggga.tattgccgcgttcgctgc
Bcen_2640,2907190-2907561,Bcen_2641                   6  ggcgtgccgcctttatcc.ctgtcaaaactgccgggt.attgccgcgttcgctgcg
Bcen2424_0465,517626-517997,Bcen2424_0466             6  ggcgtgccgcctttatcc.ctgtcaaaactgccgggt.attgccgcgttcgctgcg
Bcenmc03_0443,481908-482279,Bcenmc03_0444             6  ggcgtgccgcctttatcc.ctgtcaaaactgccgggt.attgccgcgttcgccgcg
Bcep1808_0458,494476-494849,Bcep1808_0459             8  gtgccgcctttaactccc.ctgtcaaaactgccggta.ttgctgcgcatcgcgcgc
Bcep18194_A3557,423772-424145,Bcep18194_A3558         6  gcgggctgcctttatcc.ctgtcaaatctgccgggt.atttccgcgttcgctgcg
Bmul_2931,3207212-3207586,Bmul_2932                   8  cgtgccgcctttgctcc.ctgtcaaaactgccgggc.atttcgcgtatcgacgcg
BPSL3108,3713951-3714324,BPSL3109                     5  gcgtccgccccttgctcc.ctgtcaaaactatcagtt.actttgccgatttggcgg
BURPS1710b_3646,3972049-3972421,BURPS1710b_3647       5  gcgtccgccccttgctcc.ctgtcaaaactatcagtt.actttgccgatttggcgg
```

end of page 5

putative cepBOX preceding a "catalase/peroxidase HPI" (and possibly "Ferritin, Dps family protein" and "4-hydroxybenzoate octaprenyltransferase" from the same operon)

```
Bamb_0615,690453-690885,Bamb_0616                    7   tagattatttaattaaa.ttacaaaagtcgatagat.atcgatacactcttctac
BamMC406_0640,722584-722769,BamMC406_0641            7   tagattatctttattaaa.ttacaaaagtcgatagat.gtcgatacactcttctac
BCAL3299,3615099-3615720,BCAL3301                    5   tagatgaaatttatcaaa.ttcaaataattgatagat.gaagatagaatcctctac
Bcen_0241,259944-260574,Bcen_0242                    5   tagatgaaatttatcaac.ttcaaataattgatagat.atagatagaatcctctac
Bcen2424_0725,809120-809750,Bcen2424_0726            5   tagatgaaatttatcaac.ttcaaataattgatagat.atagatagaatcctctac
Bcenmc03_0693,761967-762597,Bcenmc03_0694            5   tagatgaaatttatcaaa.ttcaaataattgatagat.atagatagaatcctctac
Bcep1808_0686,756119-756610,Bcep1808_0687            7   tagatacaagttatcaaa.ttcaaaatgtcgatagat.gaaaatagaatggtcgac
Bcep18194_A3813,711869-712511,Bcep18194_A3814        8   tagatgaaattaatcaaa.tttaaataaacgatagct.gaatatagaatcttctac
Bmul_2660,2904127-2904706,Bmul_2661                 11   aatctatcgccgaacaga.attcgattgtttaaatct.atgcgatagggaaaaata
Bphy_2521,2840892-2841271,Bphy_2522                  9   ctgcgcgatttgattata.tttatatattcgataggc.gctggctcatgcatgcgc
BURPS668_3322,3260364-3260847,BURPS668_3324         10   aaatttttatttatacgat.aggcaaaaataaacgggc.cccgaagccccgtccgat
Bxe_A3987,533677-534086,Bxe_A3986                   10   gccgccatccctatcgaa.tagataaaattaatcaaa.cacgttaattcgatagcg
```

putative cepBOX preceding "dTDP-glucose 4,6-dehydratase" (followed by "glucose-1-phosphate thymidylyltransferase", "dTDP-4-dehydrorhamnose 3,5-epimerase" and "dTDP-4-dehydrorhamnose reductase")

```
Bamb_0750,832265-833079,Bamb_0751                    5   agccttgcttgccgcaat.ttcctataattgcctgtt.tctcgcacaaaattggca
BamMC406_0760,846608-846952,BamMC406_0761            8   gatttttgaagccacttgc.ttcctataattgccgctt.cctcgacaaaattagcat
Bcen2424_0870,974580-974922,Bcen2424_0871            9   gctttttgcaggggcggc.tacctataattgtcgctt.tccccaacaaaattagca
Bcenmc03_0841,934702-935044,Bcenmc03_0842           10   gctttttgcaggggcggc.tccctataattgtcgctt.tccccaacaaaattagca
Bcep1808_0804,882799-883138,Bcep1808_0805           10   cctcaacaaaattagcat.ttcatggttttgacggtt.acggtcagcagttagaat
Bcep18194_A3968,890107-890446,Bcep18194_A3969       10   gatttttgacccaacccgc.ttcctataatcgatggcc.cctcaacaaaattagcat
BMA10229_A2752,2783479-2783714,BMA10229_A2753       10   gtctccatagtggaaacg.ttctcgaaaatgccggct.tgctagcaaacttatcga
BMA10247_1852,1834369-1834745,BMA10247_1853         10   gtctccatagtggaaacg.ttctcgaaaatgccggct.tgctagcaaacttatcga
BMA1990,2089110-2089486,BMA1991                     10   gtctccatagtggaaacg.ttctcgaaaatgccggct.tgctagcaaacttatcga
BMASAVP1_A0922,917551-917786,BMASAVP1_A0923         10   gtctccatagtggaaacg.ttctcgaaaatgccggct.tgctagcaaacttatcga
BURPS1710b_3163,3473023-3473258,BURPS1710b_3164     10   gtctccatagtggaaacg.ttctcgaaaatgccggct.tgctagcaaacttatcga
BURPS668_3105,3048557-3048933,BURPS668_3106         10   gtctccatagtggaaacg.ttctcgaaaatgccggct.tgctagcaaacttatcga
Bxe_A3728,819308-819996,Bxe_A3727                    9   cgcgacgagcgtcgcgct.gtgtcatccttgcccgta.tcttcgacattctgccgg
Bxe_A3804,741547-742011,Bxe_A3803                    9   tctagatgttggccgcag.atcggatggttggcagat.ctgccaccgcgcgttgtg
Bxe_B1714,1472424-1472812,Bxe_B1713                 10   gactgttgtaggcgaaag.tggcagaaattgaccaga.taattgcgacagtggaca
```

putative cepBOX preceding the gene "UbiD decarboxylase" (and possibly "lysine exporter LysE/YggA" from operon) or "Lytic transglycosylase" (and possibly "HMG-I&Y, DNA-binding" & "integrase " from operon)

```
Bamb_0811,896719-897314,Bamb_0812                    7   gcgattcaaaattacttt.ttgttatgattttgagat.ttcatagtgttgacgatc
BamMC406_0822,911315-911910,BamMC406_0823            7   gtgatttaaaattacttt.ttgttatgattttgagat.ttcatagtgttgacgatc
BCAL3057,3350924-3351520,BCAL3058                    5   ggggttttaaattacttt.ttgttataatttcgaggt.tttatagtgttgacgatc
Bcen_0473,514618-515114,Bcen_0474                    7   ggcgttttaaattacttt.ttgttatagatttgagtt.tttatagtgttgacgatc
Bcen2424_0952,1059164-1059759,Bcen2424_0953          7   ggcgttttaaattacttt.ttgttatagatttgagtt.tttatagtgttgacgatc
Bcenmc03_0914,1012427-1013022,Bcenmc03_0915          7   ggagttttaaattacttt.ttgttatagatttgagtt.tttatagtgttgacgatc
BMA2150,2239479-2239860,BMA2151                     10   caacactatagaaattca.tttctataacaaaagta.atgatttgttacgagtca
BMASAVP1_A0758,770047-770724,BMASAVP1_A0759         10   caacactatagaaattca.tttctataacaaaagta.atgatttgttacgagtca
Bmul_2444,2660306-2660903,Bmul_2445                  6   gtcacggtaaattacttt.ttgttataatttgaaggt.tttgtagtgttgacgatc
Bphy_0614,690837-691441,Bphy_0615                    9   agtgttcattattacaat.tggttatagttttgcggc.cttatagtgttgacgatc
BURPS1106A_3074,3002508-3003086,BURPS1106A_3075     10   caacactatagaaattcg.tttctataacaaaagta.atgatttgttacgggtcg
```

putative cepBOX preceding the gene "OmpA/MotB domain-containing protein" (and possibly "3-demethylubiquinone-9 3-methyltransferase" & "phosphoglycolate phosphatase" from operon) or "DNA gyrase subunit A"

```
Bamb_0915,1006102-1006638,Bamb_0916                  7   ctggccgccgatgggcgc.atgttagaatctcgcgtt.gcgttgcgcatgcagcgc
BamMC406_0919,1010268-1010805,BamMC406_0920          7   ctggccgccgatgggcgc.atgttagaatctcgcgtt.gcgttgcgcatgcagcgc
BCAL2957,3238399-3238953,BCAL2958                    7   ttggcggctgatgggcgc.atgttagaatctcgcgtt.gcgttgcgcatgcagcgc
Bcen_0560,607286-607810,Bcen_0561                    7   ttggcggctgatgggcgc.atgttagaatctcgcgtt.gcgttgcgcatgcagcgc
Bcen2424_1039,1151929-1152453,Bcen2424_1040          7   ttggcggctgatgggcgc.atgttagaatctcgcgtt.gcgttgcgcatgcagcgc
Bcenmc03_0998,1104641-1105215,Bcenmc03_0999          7   ttggcggctgatgggcgc.atgttagaatctcgcgtt.gcgttgcgcatgcagcgc
Bcep1808_0960,1044872-1045408,Bcep1808_0961          7   ttggctgcggatgggcgc.atgttagaatctcgcgtt.gcgttgcgcatgcagcgc
Bcep18194_A4152,1086299-1086841,Bcep18194_A4153      7   ttggcggctgatgggcgc.atgttagaatctcgcgtt.gcgttgcgcatgcagcgc
BMA0435,461903-462412,BMA0436                        5   tggggcgggttgggcgc.atgttagaatcgcacgtt.gcgtcgcgcgagcggcgc
BMA10229_A0953,975228-975872,BMA10229_A0955          5   tggggcgggttgggcgc.atgttagaatcgcacgtt.gcgtcgcgcgagcggcgc
BMA10247_0194,186826-186937,BMA10247_0195            5   tggggcgggttgggcgc.atgttagaatcgcacgtt.gcgtcgcgcgagcggcgc
Bmul_2264,2480695-2481217,Bmul_2265                  7   ttggcggctgttgggcgc.atgttagaatctcacgtt.gcgttgcgcatgcatcgc
Bphy_0737,831491-832055,Bphy_0738                    5   cggggcaggtgcgccgc.atgatagaatcgcctgat.gcgctgcgctcgcagccg
BPSL2521,3039134-3039643,BPSL2522                    5   tggggcgggttgggcgc.atgttagaatcgcacgtt.gcgtcgcgcgagcggcgc
BTH_I1631,1837465-1837998,BTH_I1632                  5   tggggcgggttgggcgc.atgttagaatcgcacgtt.gcgtcgcgcgagcggcgc
BURPS1106A_2951,2897525-2898032,BURPS1106A_2953      5   tggggcgggttgggcgc.atgttagaatcgcacgtt.gcgtcgcgcgagcggcgc
BURPS1710b_3000,3307852-3308359,BURPS1710b_3002      5   tggggcgggttgggcgc.atgttagaatcgcacgtt.gcgtcgcgcgagcggcgc
BURPS668_2888,2850273-2850384,BURPS668_2889          5   tggggcgggttgggcgc.atgttagaatcgcacgtt.gcgtcgcgcgagcggcgc
Bxe_A0974,3812415-3812899,Bxe_A0973                  5   tcggcgcggattcacggc.atgttagaatcgcgtgat.gcgttgcgcctgcaatgc
Bxe_A0975,3809643-3809765,Bxe_A0974                 10   actggttgaaagctgtgc.cgggaataatggcgtgtc.gggctgatacgtatctcg
```

end of page 6

putative cepBOX preceding a "diguanylate cyclase"

```
Bamb_1021,1113060-1113404,Bamb_1022                  10  gcgaaagcccgctgaagg.cgggcttttttgcgtgct.gcaaatttgctacggcaa
BamMC406_1025,1118645-1118990,BamMC406_1026          10  gcgaaagcccgctgaagg.cgggcttttttgcgtgct.gcaaatttgctacggcaa
Bcep18194_C7367,974362-974684,Bcep18194_C7368        10  cgacgccgggcgaacgca.cggagaggatcacgcgct.ccgtcgattgacgcgtca
BPSL0580,643113-643284,BPSL0581                      10  ccgccgaatgacggcggt.ttgcctttctcgcgtgac.tgcgtgtgctgcacgatg
BTH_I0503,558423-558594,BTH_I0504                    10  ccgccgaatgacggcggt.ttgcctgtctcgcgtggc.tgcgtgcgctgcacgatg
BTH_I0504,558913-559552,BTH_I0505                    10  acggcgggtacggtcaag.gagccgatatcgcgcgat.tgcctgaccagaagcgca
BURPS1106A_0617,586008-586922,BURPS1106A_0618        10  cctcttggttcgcctgac.tttgccatattgccagaa.gcatatttgatctgttt
BURPS1710b_0789,808613-808784,BURPS1710b_0790        10  ccgccgaatgacggcggt.ttgcctttctcgcgtgac.tgcgtgtgctgcacgatg
BURPS1710b_0790,809103-810704,BURPS1710b_0791         5  accccaaaacccaaagaa.ctgccattatcgcaagat.ttagagcaactcttggat
BURPS668_0602,571241-571578,BURPS668_0603            10  gtggggccttgcgcgcgg.ctcgtcaacttgagcggg.gaaagcggtttcgtcagg
```

putative cepBOX preceding a "phosphate ABC transporter, periplasmic phosphate-binding protein"

```
Bamb_1176,1287986-1288450,Bamb_1177                   8  aacgcgcggcggggatga.cggcaagaattgccggtc.aggcagggatggcatggg
BamMC406_1188,1314246-1314702,BamMC406_1189           8  cacgcgcggcggggactg.cggcaagaattgccggtc.cggcaaggacggtgtgga
BCAL1269,1381576-1382014,BCAL1270                     7  gcgccgcagcggcgctgg.cggcaagaattgcctgcc.tcggaggggcggggtgaa
Bcen_0818,886230-886681,Bcen_0819                     7  acgacggagcggcggtgg.cggcaagaattgcctggc.gcggagggaggggacga
Bcen2424_1299,1430866-1431317,Bcen2424_1300           7  acgacggagcggcggtgg.cggcaagaattgcctggc.gcggagggaggggacga
Bcenmc03_1270,1393997-1394448,Bcenmc03_1271           7  acgccggagcggcagtgg.cggcaagaattgcctggc.gcggagggatggggacga
Bcep1808_1252,1356089-1356543,Bcep1808_1253           5  gcggcgcgctgggaaaag.cggcaagaattgcccgtt.gcgcggagtggccgcgcc
Bcep18194_A4441,1401817-1402279,Bcep18194_A4442       7  gcgccggaacggcggtgg.cggcaagaattgcctgcc.tggaaaggatggggagac
BPSL1358,1587470-1587904,BPSL1359                    10  ggccggcgccgaaggccg.aggccgaacctatccggt.cctccttcgaggggccgc
BTH_I2773,3185742-3186167,BTH_I2774                   9  gccgcgtccgaaatttgt.cggactaaatagaaagac.acaaaatcaggcgggcgc
```

putative cepBOX preceding a "hypothetical protein"

```
Bamb_1211,1326551-1326749,Bamb_1212                  11  gcgccgcatcgaatcggctctgcc.cgttctgtcagttctgtcagttgc.tccggaacaccacacggcaccgct
BamMC406_1238,1372410-1372605,BamMC406_1239          11  gcgccgcatcgaatcggctctgcc.cgttctgtcagttctgtcagttgc.tccggaacaccacacggcaccgct
BMA10229_A2104,2125766-2126406,BMA10229_A2105        21  cgtcgggcggcgcagccgtcggct.cgaaccgacattcgaaacagcatg.gtcgcgagatgaacgcacggcccg
BMA10247_3359,3318123-3318360,BMA10247_3360          22  atcgccaccgcgggttcggccggc.cggccatcgagctttgccgagttc.gcgccgtatttccgtcgatcgcg
BMA10247_3360,3318583-3319223,BMA10247_3361          21  cgtcgggcggcgcagccgtcggct.cgaaccgacattcgaaacagcatg.gtcgcgagatgaacgcacggcccg
BMASAVP1_A2987,2970121-2970761,BMASAVP1_A2988        21  cgtcgggcggcgcagccgtcggct.cgaaccgacattcgaaacagcatg.gtcgcgagatgaacgcacggcccg
BMASAVP1_A2988,2970984-2971221,BMASAVP1_A2989        22  atcgccaccgcgggttcggccggc.cggccatcgagctttgccgagttc.gcgccgtatttccgtcgatcgcg
BPSL0265,278443-279103,BPSL0266                      21  cgtcgggcggcgcagccgtcggct.cgaaccgacattcgaaacagcatg.gtcgcgagatgaacgcacggcccg
BURPS1106A_0274,257955-258448,BURPS1106A_0275        21  tgaaacgtttcgcccggtcgactg.cgtcctgccgattcctgccgagcc.gcatcggaaatttcaccgcaaatc
BURPS1710b_0457,468551-469198,BURPS1710b_0458        21  cgtcgggcggcgcagccgtcggct.cgaaccgacattcgaaacagcatg.gtcgcgagatgaacgcacggcccg
BURPS1710b_0458,469421-469898,BURPS1710b_0459        22  atcgccaccgcgggttcggccggc.cggccatcgagctttgccgagttc.gcgccgtatttccgtcgatcgcg
BURPS668_0261,252015-252508,BURPS668_0263            22  gcgccgtattttccgtcgatcgcg.ccaattgggagcaccgcccgcttt.cccaagatgttttcacggctcgac
```

putative cepBOX preceding either a "hypothetical protein" or a "amidohydrolase"

```
Bamb_1549,1716268-1716621,Bamb_1550                   5  ccggaataattccgtcgc.atcaaagagttgagcgtt.cttgttcatttttgacg
BamMC406_1568,1759434-1759788,BamMC406_1569           7  ccagaataattccgtcac.atcaaagggttgagcgtt.cttgttcatttttgacg
Bcen_1168,1286946-1287297,Bcen_1169                   8  ccaaaataattcctttc.atcaaagggttgagagca.tttgttcatttttgacg
Bcen2424_1648,1831631-1831982,Bcen2424_1649           8  ccaaaataattcctttc.atcaaagggttgagagca.tttgttcatttttgacg
Bcenmc03_1623,1810088-1810439,Bcenmc03_1624           8  ccggaataattcctttc.atcaaagggttgagagca.tttgttcatttttgacg
Bcep18194_A4796,1811165-1811504,Bcep18194_A4797       8  cacaaaaatgtacaagc.gtcaaaaagtgaacaagt.attcgcaagatattgatg
Bmul_1589,1743952-1744154,Bmul_1590                   9  catcctgacactatgtcg.ttgacagaaaagtacttt.actcaatcataccggcgt
BPSL0089,102250-102608,BPSL0090                      10  tcggtctgtttgtacgtt.attcttaatgtgtacgtt.tttggcatacactgagag
```

putative cepBOX preceding either a "sigma-54 dependent trancsriptional regulator" or a "beta-lactamase domain-containing protein"

```
Bamb_1668,1844432-1844695,Bamb_1669                   5  gccatgtctggcagtgtc.gtggcagaattggcagtc.cgcctggcagttcgtccg
BamMC406_1665,1865388-1865651,BamMC406_1666           8  gccatgtctggcagtgtc.gtggcagaattggcagtc.ggcctggcagttcgctcg
BCAL1817,2007612-2007864,BCAL1818                    10  gccataactggcagtgtc.gtggcagaattggcggtc.agcctggcagtccgtccg
Bcen_6334,950932-951184,Bcen_6335                     8  gccacaactggcagtgtc.gtggcagaattggcagcc.agcctggcagtccgtccg
Bcen2424_1744,1940518-1940770,Bcen2424_1745           8  gccacaactggcagtgtc.gtggcagaattggcagcc.agcctggcagtccgtccg
Bcenmc03_1760,1964931-1965183,Bcenmc03_1761           8  gccacaactggcagtgtc.gtggcagaattggcagcc.agcctggcagtccgtccg
Bcep1808_1687,1835065-1835268,Bcep1808_1688           8  gccatgtgcggcagtgtc.gtggcagaattggcagtc.ggcctggcagtccgtcgg
Bcep18194_A5042,2080134-2080382,Bcep18194_A5043       8  gccacaactggcagtgtc.gtggcagaattggcagtc.cgtctggcagtttgtcag
Bmul_1515,1668535-1668825,Bmul_1516                   8  gccatgcgtggcagtcgt.gtggcagaattggcagtc.tttgtggcagtccttgtg
BTH_I2282,2571629-2571886,BTH_I2283                   5  gccgtgaatggcagtttg.atggcagaattggcagct.acgcggccgtgggccgcg
BURPS1710b_2302,2569722-2569980,BURPS1710b_2304       7  gccaaggatggcagttga.atggcagaattggcagcc.aacgcggccgcgggccgc
```

putative cepBOX preceding a "50S ribosomal protein L31 type B"

```
Bamb_1770,1958161-1958318,Bamb_1771                   9  cgtcaaccatctgatttt.ctggcataatcgccgttt.ttcgaccttaggcaagtg
BamMC406_1743,1948596-1948753,BamMC406_1744           9  cgtcaaccatctgatttt.ctggcataatcgccgttt.ttcgaccttaggcaagtg
BCAL1904,2102402-2102557,BCAL1905                     9  cgtcaaccgtctgatttt.ctggcataatcgccgttt.ttcgaccttaggcaagtg
Bcen_6246,855348-855504,Bcen_6247                     9  cgtcaaccgtctgatttt.ctggcataatcgccgttt.ttcgaccttaggcaagtg
Bcen2424_1832,2036200-2036356,Bcen2424_1833           9  cgtcaaccgtctgatttt.ctggcataatcgccgttt.ttcgaccttaggcaagtg
Bcenmc03_1856,2063993-2064149,Bcenmc03_1857           9  cgtcaaccgtctgatttt.ctggcataatcgccgttt.ttcgaccttaggcaagtg
Bcep18194_A5133,2178479-2178635,Bcep18194_A5134       9  cgtcaaccatctgatttt.ctggcataatcgccgttt.ttcgaccttaggcaagtg
BMA10229_A0037,36326-36539,BMA10229_A0039             5  cgccaagcgcctgatttt.ctggcataatcgcgagct.ttcgaccttaggcaagtg
BMA10247_1129,1124565-1124778,BMA10247_1131           5  cgccaagcgcctgatttt.ctggcataatcgcgagct.ttcgaccttaggcaagtg
BMA1368,1427243-1427456,BMA1369                       5  cgccaagcgcctgatttt.ctggcataatcgcgagct.ttcgaccttaggcaagtg
BMASAVP1_A1858,1845140-1845353,BMASAVP1_A1860         5  cgccaagcgcctgatttt.ctggcataatcgcgagct.ttcgaccttaggcaagtg
Bmul_1440,1585939-1586094,Bmul_1441                   9  cgtcaaccgtctgatttt.ctggcataatcgccgttt.ttcgaccttaggcaagtg
Bphy_0947,1058674-1058843,Bphy_0948                   9  cgtcaaccgcctgatttt.ctggcataatcgccgttt.ttcgaccttaggcaagtg
BPSL1491,1729902-1730115,BPSL1492                     5  cgccaagcgcctgatttt.ctggcataatcgcgagct.ttcgaccttaggcaagtg
BTH_I2213,2489469-2489681,BTH_I2214                   5  cgccaagcgtctgattat.ctggcataatcgcgagct.ttcgaccttaggcaagtg
BURPS1106A_2251,2239510-2239723,BURPS1106A_2253       5  cgccaagcgcctgatttt.ctggcataatcgcgagct.ttcgaccttaggcaagtg
BURPS1710b_2379,2653355-2653568,BURPS1710b_2380       5  cgccaagcgcctgatttt.ctggcataatcgcgagct.ttcgaccttaggcaagtg
BURPS668_2213,2205970-2206183,BURPS668_2215           5  cgccaagcgcctgatttt.ctggcataatcgcgagct.ttcgaccttaggcaagtg
Bxe_A2367,2291170-2291406,Bxe_A2366                   9  cgtcaagcgactgatttt.ctggcataatcgccgttt.ttcgaccataggcaagtg
```

putative cepBOX preceding either "ATP-dependent Clp protease adaptor protein ClpS" or "cold-shock DNA-binding domain-containing protein"

```
Bamb_2567,2827087-2827634,Bamb_2568                  10  atttcgccaaccggcaaa.tgaaaaagagtgccagct.tgattgttggcgctcttt
BamMC406_2437,2707442-2707990,BamMC406_2438           8  aacaatcaagctggcact.tttttaatttgccagtc.ggcgaattgaaagagggc
BCAL2731,2999914-3000458,BCAL2732                     9  aacaatcaagctggcact.tttttcaatttgccggtt.ggcgaaacgaaagaggcc
Bcen_1908,2107778-2108321,Bcen_1909                   5  gtttcgccaatcggcgaa.ttgaaaaaagtgccagct.tgattgttggcgctcttt
Bcen2424_2519,2792148-2792691,Bcen2424_2520           5  gtttcgccaatcggcgaa.ttgaaaaaagtgccagct.tgattgttggcgctcttt
Bcenmc03_2544,2838674-2839217,Bcenmc03_2545           5  gtttcgccaatcggcgaa.ttgaaaaaagtgccagct.tgattgttggcgctcttt
Bcep1808_2597,2873544-2874089,Bcep1808_2598           8  atttcgccagccggcgaa.tgaaaaaggtgccagct.tgattgttggcgctcttt
Bcep18194_A5851,2968667-2969211,Bcep18194_A5852       5  atttcgccaactggcaaa.ttgaaaaaagtgccagct.tgattgttggcgctcttt
BMA10229_A1051,1063664-1064195,BMA10229_A1052         5  caaattgtcaaagacgaa.ttgaaaaaagtgccagct.tgattgttggcgctcttt
BMA10247_2156,2114930-2115461,BMA10247_2157           5  caaattgtcaaagacgaa.ttgaaaaaagtgccagct.tgattgttggcgctcttt
BMA2279,2363888-2364419,BMA2280                       5  caaattgtcaaagacgaa.ttgaaaaaagtgccagct.tgattgttggcgctcttt
BMASAVP1_A0574,585047-585578,BMASAVP1_A0575           5  caaattgtcaaagacgaa.ttgaaaaaagtgccagct.tgattgttggcgctcttt
Bmul_0775,851894-852436,Bmul_0776                     8  tttcgcccgcgggcaatt.tgaaaaaagtgccagct.tgattgttggcgctcttt
Bphy_0567,642968-643499,Bphy_0568                     5  tttcgtatagaccgctta.ttgaaaaaagtgccagct.tgattgttgagtctcttt
BPSL0898,1047571-1048102,BPSL0898a                    5  caaattgtcaaagacgaa.ttgaaaaaagtgccagct.tgattgttggcgctcttt
BTH_I0761,876582-877069,BTH_I0762                     5  cagattgtcaaagacaaa.ttgaaaaaagtgccagct.tgattgttggcgctcttt
BURPS1106A_0962,943502-944033,BURPS1106A_0963         5  caaattgtcaaagacgaa.ttgaaaaaagtgccagct.tgattgttggcgctcttt
BURPS1710b_1113,1166979-1169374,BURPS1710b_1116      10  cgttcgacgtcgactgca.cgtgcatgatcgcccgct.ggatcacgcgctgaaagc
BURPS668_0958,935790-936320,BURPS668_0959             5  caaattgtcaaagacgaa.ttgaaaaaagtgccagct.tgattgttggcgctcttt
```

putative cepBOX preceding either "integral membrane protein MviN" or "30S ribosomal protein S20"

```
Bamb_2599,2863442-2863745,Bamb_2600                   5  gccacgcgcttgatttttg.ttgctataattgccggtt.tctgagcctgttacatgc
BamMC406_2469,2746094-2746397,BamMC406_2470           5  gccacgcgcttgatttttg.ttgctataattgccggtt.tctgagcctgttacatgc
BCAL2764,3036601-3036904,BCAL2765                     6  gccactggcttgatttttg.ttgctataatcgccggtt.tctgggctcgtacatgca
Bcen_1938,2143161-2143375,Bcen_1939                   6  gccactcgcttgatttttg.ttgctataatcgccggtt.tctgggctcgtacatgca
Bcen2424_2550,2827416-2827720,Bcen2424_2551           6  gccactcgcttgatttttg.ttgctataatcgccggtt.tctgggctcgtacatgca
Bcenmc03_2574,2873966-2874270,Bcenmc03_2575           6  gccactcgcttgatttttg.ttgctataatcgccggtt.tctgggctcgtacatgca
Bcep1808_2643,2930407-2930710,Bcep1808_2644           6  gccacgcacttgatttttg.ttgctataatcgccggtt.tctgagcctgttacatgc
Bcep18194_A5882,3004230-3004533,Bcep18194_A5883       6  gccactcgcttgatttttg.ttgctataatcgccggtt.tctgggctcgtacatgca
BMA0377,399694-400097,BMA0378                         5  gccactcgcttgatttttg.ttgttataattgccggtt.tctgagcctgtcatgcgc
BMASAVP1_A0677,689700-690032,BMASAVP1_A0678           5  gccactcgcttgatttttg.ttgttataattgccggtt.tctgagcctgtcatgcgc
Bmul_0745,817633-817936,Bmul_0746                     6  gccacgcgcttgatttttg.ttgctataatcgccggtt.tctgagcctgttacatgc
Bphy_0531,602707-603186,Bphy_0532                     6  gccacgtatctgatttttg.ttgctataatcgccggtt.tcgaagctcgctcaagct
BPSL0871,1011377-1011780,BPSL0872                     6  gccactcgcttgatttttg.ttgttataatcgccggtt.tctgagcctgtcatgcgc
BTH_I0735,845112-845446,BTH_I0736                     5  gccactcgcttgatttttg.ttgttataattgccggtt.tctgagcctgtcatacgc
BURPS668_0919,899109-899512,BURPS668_0921             5  gccactcgcttgatttttg.ttgttataattgccggtt.tctgagcctgtcatgcgc
Bxe_A0760,4042827-4043350,Bxe_A0759                   6  gccacgtgcctgatttttg.ttgctataatcgccggtt.tcgaagctcgcttaactt
```

putative cepBOX preceding either "major facilitator transporter" (possibly in the same operon as a MarR transcriptional regulator) or a "hypothetical protein"

```
Bamb_2795,3084664-3084770,Bamb_2796                   6  atcctagattactccggc.gtgctagaattttgagat.tgtttgactcgtcaaaag
BamMC406_2662,2958767-2958873,BamMC406_2663          10  ttactccggcgtgctaga.atttaagattgtttgac.tcgtcaaaagttgcatca
BCAL0860,933153-933258,BCAL0861                       5  agcctagattactccggc.atgctagaattttgagat.tctttgactcggcaaaag
Bcen_2133,2359155-2359261,Bcen_2134                   5  agcctagattactccggc.atgctagaattttgagat.tatttgacccggcaaaag
Bcen2424_2745,3043502-3043608,Bcen2424_2746           5  agcctagattactccggc.atgctagaattttgagat.tatttgacccggcaaaag
Bcenmc03_2771,3092203-3092309,Bcenmc03_2772           5  agcctagattactccggc.atgctagaattttgagat.tatttgacccggcaaaag
BMA0172,183009-183114,BMA0173                         8  ggctagattacctgatgc.atgatagaattttcgatt.cgttgacgcatcaaaagt
BMA10247_2382,2342809-2342914,BMA10247_2384           8  ggctagattacctgatgc.atgatagaattttcgatt.cgttgacgcatcaaaagt
BMASAVP1_A2775,2760296-2760401,BMASAVP1_A2777         8  ggctagattacctgatgc.atgatagaattttcgatt.cgttgacgcatcaaaagt
Bmul_0553,603548-603654,Bmul_0554                     7  ggcctagggtactcccgc.gtgctacaattttgagat.tgtttgacccgtcaaacg
BPSL0624,707625-707730,BPSL0625                       8  ggctagattacctgatgc.atgatagaattttcgatt.cgttgacgcatcaaaagt
BURPS1106A_0667,642730-642835,BURPS1106A_0669         8  ggctagattacctgatgc.atgatagaattttcgatt.cgttgacgcatcaaaagt
BURPS668_0651,627739-627844,BURPS668_0653             8  ggctagattacctgatgc.atgatagaattttcgatt.cgttgacgcatcaaaagt
```

putative cepBOX preceding either "rhodanese domain-containing protein" (possibly in the same operon as "glutaredoxin 3") or "phosphoglycerate mutase 1 family protein"

```
Bamb_2911,3209449-3209584,Bamb_2912                    8 ccatcgcgcaagacggtt.atttataatggccggat.tgtcctttccgatttct
BamMC406_2773,3072695-3072830,BamMC406_2774            8 gaaaaggacaatcccgcc.attataaaataaccatct.tgcgcgatggcggacccg
Bcen_2243,2472654-2472909,Bcen_2244                    5 actgagttatcggacgaa.cagttatacttgtccgct.acatccccgctaccgcca
Bcenmc03_2868,3190286-3190541,Bcenmc03_2869            5 actgagttatcggacgaa.cagttatacttgtccgct.acatccccgctaccgcca
BMA10229_A1411,1442082-1442214,BMA10229_A1412          5 acatcgcgtcagacggtt.atttataatggcgggat.tgccctttccgatttctc
BMA10247_2838,2795264-2795396,BMA10247_2839            8 acatcgcgtcagacggtt.atttataatggcgggat.tgccctttccgatttctc
BMA3207,3309451-3309583,BMA3208                        8 acatcgcgtcagacggtt.atttataatggcgggat.tgccctttccgatttctc
BMASAVP1_A0180,184150-184282,BMASAVP1_A0181            8 acatcgcgtcagacggtt.atttataatggcgggat.tgccctttccgatttctc
BTH_I0416,464292-464424,BTH_I0417                      8 acatcgcgtcagacggtt.atttataatggcgggat.tgccctttccgatttctc
BURPS1106A_0497,464743-464875,BURPS1106A_0498          8 acatcgcgtcagacggtt.atttataatggcgggat.tgccctttccgatttctc
BURPS1710b_0662,676043-676175,BURPS1710b_0663          8 acatcgcgtcagacggtt.atttataatggcgggat.tgccctttccgatttctc
BURPS668_0478,450087-450219,BURPS668_0479              8 acatcgcgtcagacggtt.atttataatggcgggat.tgccctttccgatttctc
```

putative cepBOX preceding "carboxyl-terminal protease"

```
Bamb_2912,3210332-3210606,Bamb_2913                   5  actgagtattcggacgaa.cagttatacttgtccgct.acatccccgctaccgcca
BamMC406_2774,3073578-3073855,BamMC406_2775           5  actgagtaatcggacgaa.cagttatacttgtccgct.acatccccgctaccgcca
BCAL0738,806117-806372,BCAL0739                       5  actgagttatcggacgaa.cagttatacttgtccgct.acatccccgctaccgcca
Bcen_2243,2472654-2472909,Bcen_2244                   5  actgagttatcggacgaa.cagttatacttgtccgct.acatccccgctaccgcca
Bcen2424_2857,3155828-3156083,Bcen2424_2858           5  actgagttatcggacgaa.cagttatacttgtccgct.acatccccgctaccgcca
Bcenmc03_2868,3190286-3190541,Bcenmc03_2869           5  actgagttatcggacgaa.cagttatacttgtccgct.acatccccgctaccgcca
Bcep1808_2957,3273975-3274222,Bcep1808_2958           5  actgagtaatcggacgaa.cagttatacttgtccgct.acatccccgctaccgcca
Bcep18194_A6186,3333256-3333528,Bcep18194_A6187       5  actgagttatcggacgaa.cagttatacttgtccgct.acatcatccccgctaccg
BMA10229_A1410,1441112-1441331,BMA10229_A1411         7  acaaggcgtccggacgaa.cagttatacttgaccgtc.ctgtcaccgtcccgttgc
BMA10247_2837,2794294-2794513,BMA10247_2838           7  acaaggcgtccggacgaa.cagttatacttgaccgtc.ctgtcaccgtcccgttgc
BMA3208,3310334-3310553,BMA3209                       7  acaaggcgtccggacgaa.cagttatacttgaccgtc.ctgtcaccgtcccgttgc
BMASAVP1_A0181,185033-185252,BMASAVP1_A0182           7  acaaggcgtccggacgaa.cagttatacttgaccgtc.ctgtcaccgtcccgttgc
Bmul_0445,491565-491821,Bmul_0446                     5  cctgacctgtcggacgaa.cagttatacttgtccgct.acatccccgctaccgcca
BPSL0442,483710-483929,BPSL0443                       7  acaaggcgtccggacgaa.cagttatacttgaccgtc.ctgtcaccgtcccgttgc
BTH_I0415,463358-463538,BTH_I0416                     7  acaaggcgtccggacgaa.cagttatacttgaccgtc.ctgtgaccgtcccgttgc
BURPS1106A_0496,463773-463992,BURPS1106A_0497         7  acaaggcgtccggacgaa.cagttatacttgaccgtc.ctgtcaccgtcccgttgc
BURPS1710b_0661,675073-675292,BURPS1710b_0662         7  acaaggcgtccggacgaa.cagttatacttgaccgtc.ctgtcaccgtcccgttgc
BURPS668_0477,449117-449336,BURPS668_0478             7  acaaggcgtccggacgaa.cagttatacttgaccgtc.ctgtcaccgtcccgttgc
Bxe_A4182,317499-317804,Bxe_A4181                     7  cctgacttggcgggtgtg.cagttatacttgtccgtc.cccatctctatcgacgct
```

putative cepBOX preceding either "3-hydroxyacyl-CoA dehydrogenase, NAD-binding" (and possibly "acyl-CoA dehydrogenase domain-containing protein" in the same operon) or "LysR family transcriptional regulator"

Bamb_2934,3237758-3237985,Bamb_2935	4	tcgaaagcattcaatgtc.aagtcagacttgacagct.tgtaaaggcatgcgggaa
BamMC406_2796,3101023-3101250,BamMC406_2797	4	tcgaaagcattcagtgtc.aagtcagacttgacagct.tgtaaaggcgtgcgggaa
BCAL0715,778938-779096,BCAL0716	4	cggaaagcatccagtgtc.aagtcagacttgacagct.tgtaaaggcgtgcgggaa
Bcen_2265,2499931-2500108,Bcen_2266	4	ccgaaagcattcagtgtc.aagtcagacttgacagct.tgtaaaggtgtgcgggaa
Bcenmc03_2890,3217561-3217738,Bcenmc03_2891	5	ccgaaagcattcagtgtc.aagtcagatttgacagct.tgtaaaggcgtgcgggaa
Bcep18194_A6221,3379970-3380218,Bcep18194_A6222	4	tcggaagcattcagtgtc.aagtcagacttgacagct.tgtaaaagcaggagggaa
BMA10229_A1381,1409481-1409634,BMA10229_A1382	8	atatcgaagttcattgtc.aaaacaggcttgacagat.tgttaaagaatgctggaa
BMA10247_2808,2762691-2762844,BMA10247_2809	8	atatcgaagttcattgtc.aaaacaggcttgacagat.tgttaaagaatgctggaa
BMA3234,3341986-3342139,BMA3235	8	atatcgaagttcattgtc.aaaacaggcttgacagat.tgttaaagaatgctggaa
BMASAVP1_A0210,216685-216838,BMASAVP1_A0211	8	atatcgaagttcattgtc.aaaacaggcttgacagat.tgttaaagaatgctggaa
Bmul_0423,464344-464585,Bmul_0424	4	cggcaaacgttcaatgtc.aagtcagacttgacagct.tgtaaaagctcgctggaa
Bphy_7223,1882379-1882499,Bphy_7224	8	tcaattaagtttatcgcc.tggacaatcgtgtgcgat.tccgcttatgccgcacga
BPSL0418,453248-453401,BPSL0419	8	atatcgaagttcattgtc.aaaacaggcttgacaggt.tgttaaagaatgctggaa
BTH_I0392,434381-434538,BTH_I0393	10	atgtcgaaatacattgtc.aaaaccggcttgacaggt.tgttaaagaatgctggaa
BURPS1106A_0469,433307-433460,BURPS1106A_0470	8	atatcgaagttcattgtc.aaaacaggcttgacaggt.tgttaaagaatgctggaa
BURPS668_0449,418883-419036,BURPS668_0450	8	atatcgaagttcattgtc.aaaacaggcttgacaggt.tgttaaagaatgctggaa

putative cepBOX preceding "UDP-N-acetylglucosamine pyrophosphorylase" (and possibly "glucosamine--fructose-6-phosphate aminotransferase" from the same operon)

Bamb_3027,3346670-3346803,Bamb_3028	7	cggaacgccagggccgc.atgctaaaatggtcggct.tcgaactcctctgacagc
BamMC406_2890,3211152-3211285,BamMC406_2891	7	cggaacgccagggccgc.atgctaaaatggtcggct.tcgaactcctctgacagc
BCAL0612,666096-666230,BCAL0613	7	cggaacggcgggagccgc.atgctaaaatggacggct.tcgaactcctctgacaag
Bcen_2366,2610289-2610422,Bcen_2367	7	cggagcgacaagggccgc.atgctaaaatggtcggct.tcgaactctgacccacgc
Bcen2424_2980,3293523-3293656,Bcen2424_2981	7	cggagcgacaagggccgc.atgctaaaatggtcggct.tcgaactctgacccacgc
Bcenmc03_3000,3338218-3338351,Bcenmc03_3001	7	cggagcgacaagggccgc.atgctaaaatggacggct.tcgaactctgacccacgc
Bcep1808_3068,3402681-3402815,Bcep1808_3069	7	cgggacgccaggggccgc.atgctaaaatggacggct.tcgaactcctctgacaag
Bcep18194_A6329,3495217-3495351,Bcep18194_A6330	7	cggaacggcgggggccgc.atgctaaaataggcggct.tcgaactcctctgacaag
BMA3380,3490003-3490561,BMA3381	5	gcgccggacggggcccgc.atgctagaatggccagct.tcgaactctccctacgaa
Bmul_2975,3256796-3256931,Bmul_2976	7	cggagcgacgggggccgc.atgctaaaatggtcggct.tcaaactcctctgacaag
Bphy_2891,3247607-3247715,Bphy_2892	7	gatgcagacacatccagc.gtgctaaaatcgcgcact.ccaaactcgtcaaatacg
BPSL0313,334872-335474,BPSL0314	5	gcgccggacggggcccgc.atgctagaatggccagct.tcgaactctccctacgaa

putative cepBOX preceding either "LysR family transcriptional regulator" or "D-isomer specific 2-hydroxyacid dehydrogenase, NAD-binding"

Bamb_3186,3523906-3524335,Bamb_3187	10	caacaaggtggccgttga.cgataaaaatagtcaaaa.atcctcactcatcgcgaa
BamMC406_3069,3410214-3410643,BamMC406_3070	10	caacaaggtggccgttga.cgataaaaatagtcaaaa.atcctcacccgtcgcgaa
BCAL0460,508215-508641,BCAL0461	10	caacaaggggccggttga.cgataaaaatagtcaaaa.atcctcgccttccgcgga
Bcen_2518,2767434-2767862,Bcen_2519	10	caacaaggggccggttga.cgataaaaatagtcaaaa.atcctcggcctctgcgga
Bcen2424_3131,3449205-3449633,Bcen2424_3132	10	caacaaggggccggttga.cgataaaaatagtcaaaa.atcctcggcctctgcgga
Bcenmc03_3147,3493379-3493807,Bcenmc03_3148	10	caacaaggggccggttga.cgataaaaatagtcaaaa.atcctcggcttctgcgga
Bcep18194_A6482,3652648-3653073,Bcep18194_A6483	10	caacaaggcccttgtaga.cgataaaaatagtcaaaa.atcctcgccttctgcgga
BMA0137,146731-147179,BMA0138	5	gaccaattgttcttaaaa.atgaaataattgagcaat.ctcactcccgaaattgcc
BMA10229_A2269,2298468-2298916,BMA10229_A2270	5	gaccaattgttcttaaaa.atgaaataattgagcaat.ctcactcccgaaattgcc
BMA10247_2346,2306662-2307110,BMA10247_2347	5	gaccaattgttcttaaaa.atgaaataattgagcaat.ctcactcccgaaattgcc
BMASAVP1_A2811,2796196-2796644,BMASAVP1_A2813	5	gaccaattgttcttaaaa.atgaaataattgagcaat.ctcactcccgaaattgcc
BPSL0116,130127-130564,BPSL0117	5	gaccaattgttcttaaaa.atgaaataattgagcaat.ctcactcccgaaattgcc
BTH_I0123,151602-152038,BTH_I0124	8	aataattgagcaatctcc.ctccttaaattgcccaat.cgagaaacgtcggaactg
BURPS1106A_0151,144948-145385,BURPS1106A_0153	8	aataattgagcaatctca.ctcccgaaattgcccaat.cgagaaacgtcggaatcg
BURPS1710b_0341,356760-357197,BURPS1710b_0342	8	aataattgagcaatctca.ctcccgaaattgcccaat.cgagaaacgtcggaatcg
BURPS668_0143,137708-138145,BURPS668_0145	8	aataattgagcaatctca.ctcccgaaattgcccaat.cgagaaacgtcggaatcg

putative cepBOX preceding either "LysR family transcriptional regulator" or "D-isomer specific 2-hydroxyacid dehydrogenase, NAD-binding"

Bamb_3186,3523906-3524335,Bamb_3187	9	atccaattattctgatcg.aagcaacaattgggtgaa.cctcggcgggtaaacggt
BamMC406_3069,3410214-3410643,BamMC406_3070	9	atccaattattctgatcg.aagcaacaattgggtgaa.cctcgttgggtaaacggg
BCAL0460,508215-508641,BCAL0461	10	atccaattattctgcttg.gagcaacaattgggtgaa.cctcgtcgaggaagtggc
Bcen_2518,2767434-2767862,Bcen_2519	10	atccaattattctgccga.gagcaacaattgggtgaa.cctcgtcgagtaaatggc
Bcen2424_3131,3449205-3449633,Bcen2424_3132	10	atccaattattctgccga.gagcaacaattgggtgaa.cctcgtcgagtaaatggc
Bcenmc03_3147,3493379-3493807,Bcenmc03_3148	10	atccaattattctgccga.gagcaacaattgggtgaa.cctcgtcgagtaaatggc
Bcep1808_3290,3630953-3631352,Bcep1808_3291	9	accgattgcgaatggatg.atccaattattctgatct.cgacaacaattaggtgaa
Bcep18194_A6482,3652648-3653073,Bcep18194_A6483	10	tgttacaacttcaaaaag.atgaagcaaatgcgtaat.tctcctacgacagcggaa
BMA0137,146731-147179,BMA0138	5	gaccaattgttcttaaaa.atgaaataattgagcaat.ctcactcccgaaattgcc
BMA10229_A2269,2298468-2298916,BMA10229_A2270	5	gaccaattgttcttaaaa.atgaaataattgagcaat.ctcactcccgaaattgcc
BMA10247_2346,2306662-2307110,BMA10247_2347	5	gaccaattgttcttaaaa.atgaaataattgagcaat.ctcactcccgaaattgcc
BMASAVP1_A2811,2796196-2796644,BMASAVP1_A2813	5	gaccaattgttcttaaaa.atgaaataattgagcaat.ctcactcccgaaattgcc
Bmul_3128,3418078-3418500,Bmul_3129	10	atccaattattctgattg.gagcaacaattgggtgaa.tctcgtccgcgaagcggc
BPSL0116,130127-130564,BPSL0117	5	gaccaattgttcttaaaa.atgaaataattgagcaat.ctcactcccgaaattgcc
BTH_I0123,151602-152038,BTH_I0124	6	gaccaattgttctgaaaa.aagaaataattgagcaat.ctccctccttaaattgcc
BURPS1106A_0151,144948-145385,BURPS1106A_0153	6	gaccaattgttcttaaaa.aagaaataattgagcaat.ctcactcccgaaattgcc
BURPS1710b_0341,356760-357197,BURPS1710b_0342	6	gaccaattgttcttaaaa.aagaaataattgagcaat.ctcactcccgaaattgcc
BURPS668_0143,137708-138145,BURPS668_0145	6	gaccaattgttcttaaaa.aagaaataattgagcaat.ctcactcccgaaattgcc

putative cepBOX close to either "hypothetical protein" or "diguanylate cyclase"
Note that this putative cepBOX would be downstream of either of those genes. Thus, if it is used with a promoter, it would be for an unknown transcript (complementary to either of those genes?).

```
Bamb_3212,3550884-3551400,Bamb_3213                     8   atggggctgcggcccgga.ctgtcgacgctgctcgct.acagctgcatgccgacgc
BCAL0428,474209-474625,BCAL0430                        10   tacgatcgcttacagccc.gggccggagccgctcgct.atgctgggtttcatattg
Bcen_2546,2796589-2797005,Bcen_2547                    10   tacgatcgcttacagccc.gggccggagccgctcgct.atgctgggtttcatattg
Bcen2424_3160,3478312-3478728,Bcen2424_3161            10   tacgatcgcttacagccc.gggccggagccgctcgct.atgctgggtttcatattg
BMA10229_A2243,2274152-2274415,BMA10229_A2244           5   aggcaaaaggttgccaac.cttccataattgacagct.acgctttgttagtacaca
BMA10247_3546,3490033-3490296,BMA10247_3547             5   aggcaaaaggttgccaac.cttccataattgacagct.acgctttgttagtacaca
BMA3395,3506162-3506456,BMA3396                        10   ccgcggatatccttgaat.gtcaaattgtagcccggc.cacgcaatagtattgcgc
Bmul_3151,3442795-3443230,Bmul_3152                    10   aaaagactctgttgttga.ttgccggtgtggccgcgt.tgctgagcggctgcatcg
Bphy_3096,3475638-3475986,Bphy_3097                     9   tggacgtgcgcggaacgc.ctggcattgtaacgcggt.cgctttgcgacgcgttcg
BPSL0080,92368-92535,BPSL0081                           5   aggcaaaaggttgccaac.cttccataattgacagct.acgctttgttagtacaca
BTH_I3232,3684707-3684949,BTH_I3233                     9   cttgatgtttacctcggc.cggaaacgcttgccagat.aaggccatgaggtgtttt
BURPS1106A_0111,108211-108378,BURPS1106A_0112           5   aggcaaaaggttgccaac.cttccataattgacagct.acgctttgttagtacaca
BURPS1710b_0307,317654-317806,BURPS1710b_0308           5   aggcaaaaggttgccaac.cttccataattgacagct.acgctttgttagtacaca
BURPS668_0096,92300-92467,BURPS668_0097                 6   aggcaaaaggttgccaac.cttccacaattgacagct.acgctttgttagtacaca
Bxe_A4469,4891234-4891599,Bxe_A4468                     8   gcattgtaacgcggcggt.gtgtgaaggctgacaggc.ggcgcggcggagccgccg
Bxe_A4470,4889666-4889838,Bxe_A4469                    10   aaagatttagcggttttt.gggtcaacgaagcgagat.ttcatatggatgaggacg
```

putative cepBOX preceding either "tRNA modification GTPase TrmE" or "hypothetical protein"

```
Bamb_3213,3552871-3553132,Bamb_3214                    10   tgaacaggctatgtttag.cttatacggttgtctgaa.acgcgtcggcggaattgc
BamMC406_3097,3439919-3440255,BamMC406_3098            10   tgaataggctatgtttat.cttatacggttgtctgaa.acgcgccggcggaattgc
Bcep18194_A6518,3690281-3690617,Bcep18194_A6519         9   agcgttgggccgctccag.caggcatactcgtccgaa.acggtggctgtgatatgg
BMA10229_A2243,2274152-2274415,BMA10229_A2244           4   aggcaaaaggttgccaac.cttccataattgacagct.acgctttgttagtacaca
BMA10247_3546,3490033-3490296,BMA10247_3547             4   aggcaaaaggttgccaac.cttccataattgacagct.acgctttgttagtacaca
BTH_I3232,3684707-3684949,BTH_I3233                    10   tcggccggaaacgcttgc.cagataaggccatgaggt.gttttggcatacatgat
BURPS1106A_0111,108211-108378,BURPS1106A_0112           4   aggcaaaaggttgccaac.cttccataattgacagct.acgctttgttagtacaca
BURPS1710b_0307,317654-317806,BURPS1710b_0308           4   aggcaaaaggttgccaac.cttccataattgacagct.acgctttgttagtacaca
BURPS668_0096,92300-92467,BURPS668_0097                 5   aggcaaaaggttgccaac.cttccacaattgacagct.acgctttgttagtacaca
Bxe_A4469,4891234-4891599,Bxe_A4468                     9   ctcaaaaaacgcagcaca.atttagctttgcgagat.aaatccgaaatcggcgcc
```

putative cepBOX preceding either "tRNA modification GTPase TrmE" or "hypothetical protein"

```
Bamb_3213,3552871-3553132,Bamb_3214                     9   aattccgccgacgcgttt.cagacaaccgtataagct.aaacatagcctgttcaga
BamMC406_3097,3439919-3440255,BamMC406_3098             9   aattccgccggcgcgttt.cagacaaccgtataagat.aaacatagcctattcaga
BMA3395,3506162-3506456,BMA3396                         9   tggaaatcaaagaaaccg.cggatatccttgaatgtc.aaattgtagcccggccac
Bmul_3151,3442795-3443230,Bmul_3152                    10   ggcctttcggcccgaccg.ctccgaaacgtctgcggt.ttcggcgatttgttacgg
Bmul_3152,3444626-3444959,Bmul_3153                    10   ccggcgcaccctgtccgt.ttcagacagtatcgaatt.tatcccggcggggataag
Bphy_3095,3474126-3474236,Bphy_3096                     8   ccgcgcatttattttcgg.tacacaaacgcgtaagct.gcgtttccgcgcgcctc
Bphy_3096,3475638-3475986,Bphy_3097                    10   tgttcgctaagacgagct.aggccaaactcagacgcg.ctaaatgtaacgtgtaca
BURPS1106A_0111,108211-108378,BURPS1106A_0112           4   aggcaaaaggttgccaac.cttccataattgacagct.acgctttgttagtacaca
BURPS668_0094,90559-90895,BURPS668_0096                 9   tggaaatcaaagaaaccg.cggatatccttgaatgtc.gaattgtagcccggccac
Bxe_A4469,4891234-4891599,Bxe_A4468                     8   gcgcatcacggctggcgc.tggtcaaagctaaaaaat.atattaatttcgatactt
```

putative cepBOX preceding either "putative DNA-binding/iron metalloprotein/AP endonuclease" or "hypothetical protein"

```
Bamb_3252,35278-35408,Bamb_3253                         8   gcggtgaccgtctcccgc.ctgctataatttctggtt.acgtcgacctacctgcgg
BamMC406_3778,751875-752006,BamMC406_3779               7   gcggcgacaggctcccgc.ctgctataatttccggtt.tacgtcgacctacctgcg
BCAM0913,1005048-1005177,BCAM0914                       7   gcggccgcctgccctcgc.ctgctatactttccggtt.acgttgacctacctgcgg
Bcen_4483,1734514-1734643,Bcen_4484                     7   ggggccgtgagcccccgc.ctgctataatttcgggtt.acgttgacctacctgcgg
Bcen2424_3881,746478-746607,Bcen2424_3882               7   ggggccgtgagcccccgc.ctgctataatttcgggtt.acgttgacctacctgcgg
Bcenmc03_3645,547214-547343,Bcenmc03_3646               7   ggggccgtgagcccccgc.ctgctataatttcgggtt.acgttgacctacctgcgg
Bcep1808_4259,1071741-1071867,Bcep1808_4260             7   ccggcgcgcagccttcgc.ctgctataatttcgggtt.acgtttacctcccgctgc
Bcep18194_B2208,2531442-2531571,Bcep18194_B2209         7   gcggcagcaggccctcgc.ctgctatactttccggtt.acgttgaccttcctgcgg
BMA10229_1708,1752775-1752874,BMA10229_1709             6   gctggcgaagcggccgcg.ctgttatactttctgtt.acgttgatttccggccg
BMA10247_A0367,324857-324956,BMA10247_A0368             6   gctggcgaagcggccgcg.ctgttatactttctgtt.acgttgatttccggccg
BMAA0334,322445-322583,BMAA0335                         6   gctggcgaagcggccgcg.ctgttatactttctgtt.acgttgatttccggccg
BMASAVP1_1515,1486699-1486798,BMASAVP1_1516             6   gctggcgaagcggccgcg.ctgttatactttctgtt.acgttgatttccggccg
Bmul_4817,1944429-1944558,Bmul_4818                     7   gcggcggccggcccttgc.ctgctatactttccggtt.actcgatattcctgcggc
Bphy_3945,917987-918100,Bphy_3946                       7   ggcgccgcctgtcccgcg.ctgctatactttcaggtt.cctttttgatgtttcggg
BPSS1759,2413467-2413605,BPSS1760                       6   gctggcgaagcggccgcg.ctgttatactttctgtt.acgttgatttccggccg
BTH_II0616,721528-721657,BTH_II0617                     7   gcgggcaaggctgccgcg.ctgttatactttttgtt.acgttgatttccggccgc
BURPS1106A_A2388,2366106-2366205,BURPS1106A_A2389       6   gctggcgaagcggccgcg.ctgttatactttctgtt.acgttgatttccggccg
BURPS1710b_A0837,1095977-1096115,BURPS1710b_A0839       6   gctggcgaagcggccgcg.ctgttatactttctgtt.acgttgatttccggccg
BURPS668_A2530,2443313-2443412,BURPS668_A2531           7   gctggcgaagggggccgcg.ctgttatactttctatt.acgttgatttccggccg
Bxe_B2825,239766-239889,Bxe_B2824                       5   tgcggccctctgcccgca.ctgctatactttaaagct.tgcctttccactttcgg
```

end of page 10

putative cepBOX preceding either "peptidase S8 & S53, subtilisin, kexin, sedolisin" or "ArsR family transcriptional regulator" (and possibly "Activator of Hsp90 ATPase 1" & "hypothetical protein" from operon)

Bamb_3301,92677-93376,Bamb_3302	9	cgtagggcgtcggtgccg.ttcaggtagctgccagtt.tccatagctgtcagatct
BamMC406_3817,809318-810005,BamMC406_3818	9	cgtagggcgtcgatgcta.ttcaggtagctgccagtt.tccatagctgtcagatct
BCAM0957,1062299-1062945,BCAM0958	9	cgtagggcctcggtgccg.ttcgggtagctgccagtt.tccatagctgtcagttct
Bcen_4442,1676601-1677245,Bcen_4443	4	cgttcgggtagctgccat.tttccatagctgtcagtt.ctgacaatggacgcatg
Bcen2424_3924,803876-804520,Bcen2424_3925	4	cgttcgggtagctgccat.tttccatagctgtcagtt.ctgacaatggacgcatg
Bcenmc03_3603,489302-489946,Bcenmc03_3604	4	cgttcgggtagctgccat.tttccatagctgtcagtt.ctgacaatggacgcatg
BPSS1733,2380767-2381112,BPSS1734	10	gtatgcgagcaccgatct.ctcgggtaactgccagtt.tcgttagctgtcactgtt
BTH_II0646,756614-757032,BTH_II0647	10	gtatgcgagcaccgttct.ctcgggtaactgccagtt.tcgttagctgtcactgtt
BURPS1106A_A2357,2333409-2333814,BURPS1106A_A2358	10	gtatgcgagcaccgatct.ctcgggtaactgccagtt.tcgttagctgtcactgtt
BURPS1710b_A0810,1063253-1063598,BURPS1710b_A0811	10	gtatgcgagcaccgatct.ctcgggtaactgccagtt.tcgttagctgtcactgtt
BURPS668_A2495,2410615-2411020,BURPS668_A2497	10	gtatgcgagcacggatct.ctcgggtaactgccagtt.tcgttagctgtcactgtt

putative cepBOX preceding either "peptidase S8 & S53, subtilisin, kexin, sedolisin" or "ArsR family transcriptional regulator" (and possibly "Activator of Hsp90 ATPase 1" & "hypothetical protein" from operon)

Bamb_3301,92677-93376,Bamb_3302	5	cgttcaggtagctgccag.tttccatagctgtcagat.ctgacatcggcttgctcg
BamMC406_3817,809318-810005,BamMC406_3818	5	tattcaggtagctgccag.tttccatagctgtcagat.ctgacatcggcgggctcg
BCAM0957,1062299-1062945,BCAM0958	4	cgttcgggtagctgccag.tttccatagctgtcagtt.ctgacaactgggcgcatg
Bcen_4442,1676601-1677245,Bcen_4443	4	cgttcgggtagctgccat.tttccatagctgtcagtt.ctgacaatgggacgcatg
Bcen2424_3924,803876-804520,Bcen2424_3925	4	cgttcgggtagctgccat.tttccatagctgtcagtt.ctgacaatgggacgcatg
Bcenmc03_3603,489302-489946,Bcenmc03_3604	4	cgttcgggtagctgccat.tttccatagctgtcagtt.ctgacaatgggacgcatg
BPSS1733,2380767-2381112,BPSS1734	10	ctctcgggtaactgccag.tttcgttagctgtcactg.ttgacagagaaaaagtga
BTH_II0646,756614-757032,BTH_II0647	10	ctctcgggtaactgccag.tttcgttagctgtcactg.ttgacagagaaaaaatgg
BURPS1106A_A2357,2333409-2333814,BURPS1106A_A2358	10	ctctcgggtaactgccag.tttcgttagctgtcactg.ttgacagagaaaaagtga
BURPS1710b_A0810,1063253-1063598,BURPS1710b_A0811	10	ctctcgggtaactgccag.tttcgttagctgtcactg.ttgacagagaaaaagtga
BURPS668_A2495,2410615-2411020,BURPS668_A2497	10	ctctcgggtaactgccag.tttcgttagctgtcactg.ttgacagagaaaaagtga

putative cepBOX preceding either "peptidase S8 & S53, subtilisin, kexin, sedolisin" or "ArsR family transcriptional regulator" (and possibly "Activator of Hsp90 ATPase 1" & "hypothetical protein" from operon)

Bamb_3301,92677-93376,Bamb_3302	6	agccccacgagcaagccg.atgtcagatctgacagct.atggaaactggcagctac
BamMC406_3817,809318-810005,BamMC406_3818	6	aggagtgcgagcccgccg.atgtcagatctgacagct.atggaaactggcagctac
BCAM0957,1062299-1062945,BCAM0958	5	gcgcacgcatgcgcccag.ttgtcagaactgacagct.atggaaactggcagctac
Bcen_4442,1676601-1677245,Bcen_4443	5	gcgcactcatgcgtccca.ttgtcagaactgacagct.atggaaaatggcagctac
Bcen2424_3924,803876-804520,Bcen2424_3925	5	gcgcactcatgcgtccca.ttgtcagaactgacagct.atggaaaatggcagctac
Bcenmc03_3603,489302-489946,Bcenmc03_3604	5	gcgcactcatgcgtccca.ttgtcagaactgacagct.atggaaaatggcagctac
BPSS1733,2380767-2381112,BPSS1734	7	gggtgtgtcactttttct.ctgtcaacagtgacagct.aacgaaactggcagttac
BTH_II0646,756614-757032,BTH_II0647	8	gggtgtgccatttttct.ctgtcaacagtgacagct.aacgaaactggcagttac
BURPS1106A_A2357,2333409-2333814,BURPS1106A_A2358	7	gggtgtgtcactttttct.ctgtcaacagtgacagct.aacgaaactggcagttac
BURPS1710b_A0810,1063253-1063598,BURPS1710b_A0811	7	gggtgtgtcactttttct.ctgtcaacagtgacagct.aacgaaactggcagttac
BURPS668_A2495,2410615-2411020,BURPS668_A2497	7	gggtgtgtcactttttct.ctgtcaacagtgacagct.aacgaaactggcagttac

putative cepBOX preceding "porin"

Bamb_3374,170665-171108,Bamb_3375	6	caacgaccgttcctcttt.ttgaaacagttgacattt.gttgcggcgcgattgttg
BamMC406_3876,872784-873225,BamMC406_3877	6	caacgaccgttcctcttt.ttgaaacagttgacattt.gttgcggcgcgattgttg
BCAM1015,1129433-1129847,BCAM1016	4	cagacactgttcctgttt.atgaaatagttgacaatt.gctgcgctgcgattgttt
Bcen_4384,1611578-1611993,Bcen_4385	4	cagagactgttcctgttt.atgaaatagttgacaatt.gctgcgctgcgattgttt
Bcen2424_3981,869128-869543,Bcen2424_3982	4	cagagactgttcctgttt.atgaaatagttgacaatt.gctgcgctgcgattgttt
Bcenmc03_3546,424254-424670,Bcenmc03_3547	4	cagagactgttcctgttt.atgaaatagttgacaatt.gctgcgctgcgattgttt
BMAA1698,1854822-1855162,BMAA1699	10	ggcgtctcccgacgccgc.aagccgcagttgccaagc.cagcctgacgcgtatgcg
Bmul_4599,1700650-1701090,Bmul_4600	5	cgcgaactgttcctgttt.ttgaaatagttgacattt.gctgcgctgcgattgttg
BPSS1679,2307188-2309026,BPSS1680	10	ggacggtgcatggcgtga.cggcaagcgtcgaccggc.atgcatcgcggcgacgtt
BURPS1106A_A2276,2258290-2258747,BURPS1106A_A2277	9	cgttggccgattgttgcg.ttgcgattgttgcgacaa.taggtgtcccggg cgtgg
BURPS1710b_A0744,988145-988463,BURPS1710b_A0745	9	cgttggccgattgttgcg.ttgcgattgttgcgacaa.taggtgtcccgggcgtgg
Bxe_B0769,2510702-2511113,Bxe_B0768	9	ctagcttttttggccgtt.ttgcaggagttgcataaa.aatcgcgatgcgttttgc

putative cepBOX preceding "porin"

Bamb_3374,170665-171108,Bamb_3375	10	caaaaagaggaacggtcg.ttgatgtgcttgatatat.atagacttttcacgctg
BamMC406_3876,872784-873225,BamMC406_3877	10	caaaaagaggaacggtcg.ttgatgtgcttgatatat.atagacttttcacgctg
BCAM1015,1129433-1129847,BCAM1016	10	cataaacaggaacagtgt.ctgatgtgcttgatatat.atggacttttcctgctg
Bcen_4384,1611578-1611993,Bcen_4385	10	cataaacaggaacagtct.ctgatgtgcttgatatat.atggacttttcctgctg
Bcen2424_3981,869128-869543,Bcen2424_3982	10	cataaacaggaacagtct.ctgatgtgcttgatatat.atggacttttcctgctg
Bcenmc03_3546,424254-424670,Bcenmc03_3547	10	cataaacaggaacagtct.ctgatgtgcttgatatat.atggacttttcctgctg
Bcep1808_4483,1328021-1328418,Bcep1808_4484	10	caaaaacaggaacagttg.ttgatgtgcttgatatat.atggatttttcgctctg
Bcep18194_B2103,2410357-2410747,Bcep18194_B2104	9	cagccacggccgaaaccg.atgactggctttccagtt.cggcggcaaggcgtccgg
Bmul_4599,1700650-1701090,Bmul_4600	5	cgcgaactgttcctgttt.ttgaaatagttgacattt.gctgcgctgcgattgttg
Bmul_4600,1702252-1702540,Bmul_4601	10	cagtcgcggccgaaaccg.gtgactggctttcagtt.cggcggcagcgcgtcgag

end of page 11

putative cepBOX preceding either "hypothetical protein" or "hypothetical protein" (putative lipoprotein)

```
Bamb_3439,236053-236594,Bamb_3440                    7   aggcgggcggcgccgtgc.ttgtaaaaacggcagct.tggtggcgcaacgtgcgg
BamMC406_3946,948128-948669,BamMC406_3947            8   gagcgggcggcgccgtgc.ttgtaaaaaccggcaatt.ctgtggcgtaacgtggcg
Bcen_4321,1543212-1543435,Bcen_4322                  4   aacgatggccggcgacgg.ctgtaattcttgccagat.tcggccatcgtttaacca
Bcenmc03_3480,350486-350799,Bcenmc03_3481            5   aacgatggccgacgacgg.ctgtaattcttaccagat.ttggccatcgtttaacca
BMA10229_A0490,513628-514296,BMA10229_A0491         10   caaacaatgtgcggcgcg.tcgacggtctcgcccgct.tttttggggtcgtgcgtg
BMA10247_0666,646815-647483,BMA10247_0667           10   caaacaatgtgcggcgcg.tcgacggtctcgcccgct.tttttggggtcgtgcgtg
BMASAVP1_A1383,1355958-1356694,BMASAVP1_A1385       10   caaacaatgtgcggcgcg.tcgacggtctcgcccgct.tttttggggtcgtgcgtg
Bphy_5210,2336414-2336574,Bphy_5211                  8   gcgaacacagtgttgcaa.cttcaatagctgattgct.ggacgacgcagatccata
BPSL2036,2432319-2432686,BPSL2037                   10   caaacaatgtgcggcgcg.tcgacggtctcgcccgct.tttttggggtcgtgcgtg
BURPS1106A_1621,1577322-1577529,BURPS1106A_1622     10   caaacaatgtgcggcgcg.tcgacggtctcgcccgct.tttttggggtcgtgcgtg
BURPS1710b_1781,1942311-1943164,BURPS1710b_1782     10   caaacaatgtgcggcgcg.tcgacggtctcgcccgct.tttttggggtcgtgcgtg
BURPS668_1599,1567461-1567788,BURPS668_1600         10   caaacaatgtgcgacgcg.tcgacggtctcgcccgct.tttttggggtcgtgcgcg
```

putative cepBOX preceding "putative CheA signal transduction histidine kinases" (and possibly "methyl-accepting chemotaxis sensory transducer" from the same operon)

```
Bamb_3461,258786-259234,Bamb_3462                    9   gagattaacggccgatcc.aggaaaaactttaggatt.ttcctgccgcaattaacc
BamMC406_3968,971480-971974,BamMC406_3969           10   gagattaacggccgatcc.gggaaaaactttaggatt.ttctcgccgcaattgacc
Bcenmc03_3455,323212-323732,Bcenmc03_3456            7   tgatcggcgccgcgtttg.cgcaaagactcgcgaagt.actccgcccgaacaagcg
Bcep18194_B1979,2263835-2264290,Bcep18194_B1980      9   gaggttaacggcagattt.cggcgatactttagggct.ctttcggcacaattattc
Bxe_B3012,18355-18632,Bxe_B3011                      4   acggttaacggcgttttta.ttcaaaaacttgagagat.aaatttaatgcgttaaat
```

putative cepBOX preceding "Propeptide, peptidase M4 and M36"

```
Bamb_3835,708326-708936,Bamb_3836                    5   aggcatgctaggaggtcg.ttttataattacaagat.attttcagatagattaat
BamMC406_4300,1360028-1360433,BamMC406_4301          5   aggcatgctaggaggtcg.cttttataattacaagat.attttcagagagattaat
BCAS0407,477391-478547,BCAS0409                      5   agggatgctagaaggtga.ttttataattacaagat.attttctgttggattaat
Bcen_1232,1357942-1359175,Bcen_1233                  5   ggggatgctaggaggtcg.ttttataattacaagat.attttctgtgatattgat
Bcen2424_6599,824815-826048,Bcen2424_6600            5   ggggatgctaggaggtcg.ttttataattacaagat.attttctgtgatattgat
Bcenmc03_6203,203697-204483,Bcenmc03_6204            5   ggggatgctaggaggtcg.ttttataattacaagat.attttctttgttattaat
Bcep18194_C7164,744542-745156,Bcep18194_C7165        5   agggatgctagaaggtca.ttttataattacaagat.attttctgctagtttaat
BMA10229_2046,2102199-2102695,BMA10229_2047          6   aggcatgctaggaggaca.atttaatatttacaagat.atttaccaaaaaattaat
BMA10247_A0724,674347-674903,BMA10247_A0725          6   aggcatgctaggaggaca.atttaatatttacaagat.atttaccaaaaaattaat
BMAA1558,1683488-1683757,BMAA1559                    6   aggcatgctaggaggaca.atttaatatttacaagat.atttaccaaaaaattaat
BPSS1554,2108336-2109378,BPSS1555                    6   aggcatgctaggaggaca.atttaatatttacaagat.atttaccaaaaaattaat
BTH_II0816,959517-959708,BTH_II0817                  6   aggcatgctaggaggtca.atttaatatttacaagat.atttaccaaaaaattaat
BURPS1106A_A2102,2061077-2061635,BURPS1106A_A2104    6   aggcatgctaggaggaca.atttaatatttacaagat.atttacaaaaaaattaat
BURPS1710b_A0598,788517-789135,BURPS1710b_A0599      6   aggcatgctaggaggaca.atttaatatttacaagat.atttaccaaaaaattaat
BURPS668_A2190,2122545-2122993,BURPS668_A2191        6   aggcatgctaggaggaca.atttaatatttacaagat.atttaccaaaaaattaat
```

putative cepBOX preceding "peptidase M23B"

```
Bamb_4030,949165-949431,Bamb_4031                    6   cggtaatacggtcgaatt.cggaaaaacttgacggct.ttgggcgtacgatgcggc
BamMC406_4488,1590646-1590912,BamMC406_4489          4   cggtaatacggtcgaatt.cggaaaaacttgacagct.ttgggcgcacgatgcggc
BCAM1780,1996870-1997105,BCAM1780A                   9   cggtaatacggtccgatc.cggaaaaacttgatggtg.ttttcgcagaaaaacgga
Bcen_3767,907106-907604,Bcen_3768                    9   cggtaatacggtccgacc.cggaaaaacttgatggtc.ttttcgctgaagaacgga
Bcen2424_4600,1573520-1574018,Bcen2424_4601          9   cggtaatacggtccgacc.cggaaaaacttgatggtc.ttttcgctgaagaacgga
Bcenmc03_5704,2860929-2861164,Bcenmc03_5705          9   cggtaatacggtccgatc.cggaaaaacttgatggtc.gtttcgctgaaaaacgga
Bcep1808_5126,2035376-2035605,Bcep1808_5127          6   cggtaatacggtcggaat.cggaaaaacttgacggtt.tcccgcacgccaggcgga
Bcep18194_B1243,1406023-1406554,Bcep18194_B1244      9   cggtaatacggtccgatt.cagaaaaacttgatggtc.ttttgcaggaaagcgga
Bmul_4046,1026950-1027175,Bmul_4047                  9   cgatttcccgagagggcg.cggtcatagttacctaaa.tcgcacggggcgccggtc
BPSS0945,1248021-1248194,BPSS0946                    9   gcactcctgtgacgagag.ctgatacgctagcgagcc.gcgatgtcgggtgcgcgc
BTH_II1451,1715352-1715513,BTH_II1452               10   aaacgcgcgttcgaccct.ctaaaaatatcgaacggc.gcccatttcgggtgaacg
```

putative cepBOX preceding "cold-shock DNA-binding domain-containing protein"

```
Bamb_4063,983998-984324,Bamb_4064                    7   tgacaaacggatggctca.atgaaacagttacaacat.aacgattgacggcggcac
BamMC406_4521,1626412-1626737,BamMC406_4522          7   cgccaaacggataacccca.atgaaacagttacaacat.aacgattgacggcggcac
BCAM1809,2029035-2029415,BCAM1810                    8   gtcatcgtaggctacgct.gcgaaacagttacaagat.aacggttgacgcgagccc
Bcen_5524,42648-42954,Bcen_5525                      7   cggcgcgtcgagctggat.gtcacacagttacaagat.agtcgttgacgcggccgg
Bcen2424_5888,20469-20775,Bcen2424_5889              7   cggcgcgtcgagctggat.gtcacacagttacaagat.agtcgttgacgcggccgg
Bcenmc03_6397,432634-432938,Bcenmc03_6398            7   cggcgagtcgagctggat.gtcacacagttacaagat.agtcgttgacgcgacagg
Bcep1808_4970,1854961-1855465,Bcep1808_4971          8   gcgaaaacggacggcccg.gcgacacagttacaagat.aagcgttgactcaagcat
Bcep18194_B1151,1294065-1294574,Bcep18194_B1152      7   cgcaaaacggaccgtcag.gggaaacagttacaagtt.aacgattgacgtcaccac
Bphy_4994,2091772-2092112,Bphy_4995                  5   gtcggctgcgggagcctg.ctgaaacagttacaagat.aacgattgacgctgccga
BPSS1364,1866892-1867542,BPSS1364a                   8   ccgtcgatgatgtcgccg.ctgatcgaggtgcgcgat.gccgttgcaaccggcagg
BTH_II1001,1188476-1189125,BTH_II1002                8   cgttcggtcaagacattt.ctgaaaaacttcgaaat.ccgcgttcgacgcgcgat
BURPS1710b_A0388,547605-548255,BURPS1710b_A0389      8   ccgtcgatgatgtcgccg.ctgatcgaggtgcgcgat.gccgttgcaaccggcagg
Bxe_B2780,285272-285566,Bxe_B2779                   10   agcgattcgcagcgtccg.gcaaaacagttacaagtt.aagtgttgacgctgactc
Bxe_B2781,284780-285067,Bxe_B2780                   10   gtcgcgcgaacggtctga.ttcaagctattgcgagcc.gaatcctgacgcagtcgg
```

putative cepBOX close to "RpiR family transcriptional regulator" or "ABC transporter related"
Note that this putative cepBOX would be downstream of either of those genes. Thus, if it is used with a promoter, it would be for an unknown transcript (complementary to either of those genes?).

```
Bamb_4138,1057883-1057956,Bamb_4139                  5   cgtacgggtgctccacga.catcaatagttgaaagct.catgcccgaatgacgatc
BamMC406_4602,1708731-1708850,BamMC406_4603          8   aagtcgcaagccccgcga.cgtcgatagctgaaagct.catgcccgaatgacgatc
BCAM1955,2172415-2172788,BCAM1956                   10   gatgtatctcggatgctt.cgttttaaattcagtggt.taccctaggttttggtac
Bcen_3612,739675-740046,Bcen_3613                    9   ggttaccctaggttttgg.tacaaaaaagcgaatgat.atgatcgcggcctcgctg
Bcen2424_4754,1739621-1739992,Bcen2424_4755          9   ggttaccctaggttttgg.tacaaaaaagcgaatgat.atgatcgcggcctcgctg
Bcenmc03_5528,2672044-2672415,Bcenmc03_5529          9   ggttaccctaggttttgg.tacaaaaaagcgaatgat.atgatcgcgacctcgctg
Bcep1808_5277,2205489-2205847,Bcep1808_5278         10   tccgccgatcgtgtaacg.aatcaaacattctccgat.ctcacgcgcgggtttcgc
Bcep18194_B0995,1117578-1117940,Bcep18194_B0996     10   gataaccctaggttttag.cgaaaaaaagcgagtgct.atgatcgcggcctcgccg
```

putative cepBOX preceding "hypothetical protein"

```
Bamb_4573,1527820-1527910,Bamb_4574                  10  tacgcgatgacggaagct.atgcaagaatctttcgtc.ttgtaatcgcataacaac
BamMC406_5097,2268307-2268398,BamMC406_5098          10  tacgcgatgacggaagct.atgcaagaatctttcgtc.ttgtaatcgcataacaac
BCAM2417,2721338-2722299,BCAM2418                     9  caaacaatccttactgaa.atgaaagaatgcaggtt.gcgcaaacgtttgctatt
Bcenmc03_5048,2120136-2121097,Bcenmc03_5049          10  gacacgtcgcgccgtcct.cggcatgactgacaaatt.gccgcaccccggatctga
Bcenmc03_5049,2122001-2122529,Bcenmc03_5050           9  tacgcgatgacggaagct.gtgcaagaatctttcgat.ccgtaatcgcataacaat
Bcep1808_3477,182308-182399,Bcep1808_3478            10  tacgcgctgacgaaagct.atgcaagaatctttcgtc.ttgtaatcgtataacaat
BMA10229_0907,946025-946122,BMA10229_0908             6  tacgtgattcatcgagct.atgcaaaaatcgtcggct.cctgacatcacgatacca
BMA10247_A1876,1803789-1803886,BMA10247_A1877         6  tacgtgattcatcgagct.atgcaaaaatcgtcggct.cctgacatcacgatacca
BPSS1428,1943663-1943760,BPSS1429                     6  tacgtgattcatcgagct.atgcaaaaatcgtcggct.cctgacatcacgatacca
BTH_II0962,1147506-1147677,BTH_II0963                 9  tacgtgattcctcgagct.atgcaagaatcgtccgct.cctgacatcacgatacca
BURPS1710b_A0452,623942-624081,BURPS1710b_A0453       5  tacgtgattcatcgagct.atgcaaaaatcgtcggct.cctgacatcacgatacca
BURPS668_A2032,1962837-1962934,BURPS668_A2033         6  tacgtgattcatcgagct.atgcaaaaatcgtcggct.cctgacatcacgatacca
Bxe_B1013,2240793-2240952,Bxe_B1012                   6  tcgcgtcggcgcgcagcg.ccgccggacttgtgcagt.ccggcatcggaatttgaa
```

putative cepBOX preceding "tryptophan halogenase, PrnA" (and possibly PrnB, C and D from the same operon)

```
Bamb_4725,1718136-1718840,Bamb_4726                  10  gtcaatcaagtcgttcat.ttcgcattttatacaat.aaattcgttgacgcacac
BamMC406_5263,2476374-2477051,BamMC406_5264          10  gtcaattaaatcgttcat.ttcgcattttatacaat.aaattcgttggcgcacac
Bcep18194_C6705,220713-220993,Bcep18194_C6706         5  ggaaagcaaataaaaatc.ctgtcaaatttatcagtt.taatttaagtaatcgccg
BURPS1710b_A1114,1394092-1394966,BURPS1710b_A1115    10  ttctttcaagaaaaata.aatctttgttataaaat.atttcaccgatattttcc
```

putative cepBOX preceding "tryptophan halogenase, PrnA" (and possibly PrnB, C and D from the same operon)

```
Bamb_4725,1718136-1718840,Bamb_4726                   9  aaagtaaatttctcgttt.ttaaaaaattaaatcgtt.tgttatcggatactccgc
BamMC406_5263,2476374-2477051,BamMC406_5264           9  aaagtaaatttctcattt.ttaaaaaattaaatcgtt.tgttatcggaatctccgc
Bcenmc03_6982,1094614-1095393,Bcenmc03_6983           9  aaaatgaatttcttgtt.tgataaaagtaaatcgtt.tggcgtgggatatttcat
Bcep18194_C6705,220713-220993,Bcep18194_C6706         4  ggaaagcaaataaaaatc.ctgtcaaatttatcagtt.taatttaagtaatcgccg
```

putative cepBOX preceding "tryptophan halogenase, PrnA" (and possibly PrnB, C and D from the same operon)

```
Bamb_4725,1718136-1718840,Bamb_4726                  20  aacagcctcagacgaaagtctggg.actgcggccaatgatgtccggatt.ttgcgcggaggggcatctcttgc
BamMC406_5263,2476374-2477051,BamMC406_5264          20  aacagcctcggacgaaattccggg.actgcggccaatgatgtccggatt.ttgcgcggaggggcgtcccttgc
Bcenmc03_6982,1094614-1095393,Bcenmc03_6983          22  atgtatccccgcggacatgaaggc.catgcgatgaagacgagcaagttc.acgacagcacgggtcgcatttgca
Bcep18194_C6705,220713-220993,Bcep18194_C6706        11  taaatccgtcggaaagcaaataaa.aatcctgtcaaatttatcagttta.atttaagtaatcgccgacctattc
BURPS1710b_A1114,1394092-1394966,BURPS1710b_A1115    19  aacggtctcggatcaaactccggg.aatgcaggcaaggctgtccggata.ttgcgtagagggacgttcttcatt
```

putative cepBOX preceding "tryptophan halogenase, PrnA" (and possibly PrnB, C and D from the same operon)

```
Bamb_4725,1718136-1718840,Bamb_4726                  22  gcaaaatccggacatcattggccg.cagtcccagacttcgtctgaggc.tgttccgctatcaatgaaaataga
BamMC406_5263,2476374-2477051,BamMC406_5264          21  gcaaaatccggacatcattggccg.cagtcccggaatttcgtccgaggc.tgttccgctttcaattcaaataga
Bcenmc03_6982,1094614-1095393,Bcenmc03_6983          21  tcgacccaaaaataatccgactcg.tcagcagagaaattcgtcatgatg.tatccccgcggacatgaaggccat
Bcep18194_C6705,220713-220993,Bcep18194_C6706        11  gaataggtcggcgattacttaaat.taaactgataaatttgacaggatt.tttatttgctttccgacggattta
BURPS1710b_A1114,1394092-1394966,BURPS1710b_A1115    20  tcggaaacggcgacgaccctattt.ctttccggcaaattcatccgtcca.ttccgcactttatacaatgaatt
```

putative cepBOX preceding "tryptophan halogenase, PrnA" (and possibly PrnB, C and D from the same operon)

```
Bamb_4725,1718136-1718840,Bamb_4726                  19  gcaagagatgccccctccgcgcaa.aatccggacatcattggccgcagt.cccagactttcgtctgaggctgtt
BamMC406_5263,2476374-2477051,BamMC406_5264          19  gcaagggacgccccctccgcgcaa.aatccggacatcattggccgcagt.cccggaatttcgtccgaggctgtt
Bcep18194_C6705,220713-220993,Bcep18194_C6706        11  taaatccgtcggaaagcaaataaa.aatcctgtcaaatttatcagttta.atttaagtaatcgccgacctattc
BURPS1710b_A1114,1394092-1394966,BURPS1710b_A1115    18  aatgaagaacgtccctctacgcaa.tatccggacagccttgcctgcatt.cccggagtttgatccgagaccgtt
```

putative cepBOX preceding "TonB-dependent hemoglobin/transferrin/lactoferrin family receptor" (and possibly "hemin-degrading family protein" and "hemin importer ATP-binding subunit" from the same operon)

```
Bamb_4771,1762735-1762967,Bamb_4772                  21  ataacgagcccgaaggctggctgg.gtgacgggttcacctggctggccc.gttcgcgcgcgacaggcggcaaac
BamMC406_5314,2535384-2535617,BamMC406_5315          21  ataacgagcccgaaggctggctgg.gtgacgggttcacctggctggccc.gttcgcgcgcgacaggcgccgaac
BCAM2625,2974118-2974341,BCAM2626                    22  ctttcacatcgccgtttgccgcct.gtcgcgcgcaaacgggccagccag.gtgaacgcgtcacccagccagcct
Bcen_5431,2738024-2738250,Bcen_5432                  22  ctttcaaatcgccgtttgccgctt.gccgcgcgcaaacgggccagccag.gtgaacgcgtcacccagccagcct
Bcen2424_5430,2528756-2528982,Bcen2424_5431          22  ctttcaaatcgccgtttgccgctt.gccgcgcgcaaacgggccagccag.gtgaacgcgtcacccagccagcct
Bcenmc03_4838,1864118-1864344,Bcenmc03_4839          22  ctttcaaatcgccgtttgccgcat.gccgcgcgcaaacgggccagccag.gtgaacgcgtcacccagccagcct
Bcep18194_B0221,239405-239642,Bcep18194_B0222        10  cttgtattgataataattctcatt.tacactggtaaagttatcagttgc.ctttcaattcgccgctcgccgcct
BMA10229_1114,1162339-1162813,BMA10229_1115          20  tgcccgcgcgggcgattcgcgcgg.gcggcggtgcaacatggcgggcgg.cgatattcgccgcgacggcgcccc
BMA10247_A2088,2020210-2020571,BMA10247_A2089        22  acggtctgtcgcggccgacacctt.ctcgcagagacgctcgcgaggcca.ggggccgcacgcgaggctgagcgg
BMAA1825,1991111-1991732,BMAA1826                    20  tgcccgcgcgggcgattcgcgcgg.gcggcggtgcaacatggcgggcgg.cgatattcgccgcgacggcgcccc
BMASAVP1_0823,833033-833507,BMASAVP1_0824            20  tgcccgcgcgggcgattcgcgcgg.gcggcggtgcaacatggcgggcgg.cgatattcgccgcgacggcgcccc
Bmul_3338,225194-225433,Bmul_3339                    22  aggctggctgggcgacgcgttcac.ctggctggcccatgcgcgcgatgc.ggcgcacggcaaattgaaaggaag
BPSS0244,333185-333658,BPSS0245                      22  acggtctgtcgcggccgacacctt.ctcgcagagacgctcgcgaggcca.gggcccgcacgcaaggctgagcgg
BTH_II2138,2626035-2626526,BTH_II2139                22  gccctgggccggccgccgaacgcc.ccatccgaattgcctaccggccgc.cggcgcgcagcgccgagattcgca
BURPS1106A_A0347,332732-333204,BURPS1106A_A0348      22  acggtctgtcgcggccgacacctt.ctcgcagagacgctcgcgaggcca.gggcccgcacgcgaggctgagcgg
BURPS1710b_A1781,2171515-2171974,BURPS1710b_A1782    20  tgcccgcgcgggcgattcgcgcgg.gcggcggtgcaacatggcgggcgg.cgatattcgccgcgacggcgcccc
BURPS668_A0440,405989-406350,BURPS668_A0441          22  acggtctgtcgcggccgacacctt.ctcacagagacgctcgcgaggcca.gggcccgcacgcgaggctgagcgg
```

end of page 13

putative cepBOX preceding "hypothetical protein"

```
Bamb_4970,1988260-1988386,Bamb_4971                  10  tgtacaaacatacagtat.ttccgcaactggccgtt.cggccagctatccgcatt
Bcen_5158,2443932-2444089,Bcen_5159                   5  cggcgcaacggcctgcta.ctgttatagtcggtcgtt.cggttgaccgcatttatc
Bcep1808_3788,535946-536105,Bcep1808_3789             5  cagcgcgacgggctgcta.ctgttatagtcggtcgtt.cggttgaccgcatttatc
BTH_II0004,3559-3725,BTH_II0005                       7  cggcaaagcagcttgcta.ctgttatagtcggccgtc.gggtccagcgtattttc
Bxe_B3022,9240-9400,Bxe_B3021                        10  tcggaaaaacgaaaatat.ccgataaaactgacagcc.gactataacaggccgcct
```

putative cepBOX preceding either "XRE family transcriptional regulator" or "2-amino-3-ketobutyrate coenzyme A ligase" (and possibly "L-threonine 3-dehydrogenas" from the same operon)

```
Bamb_4971,1988999-1989159,Bamb_4972                   5  cggcgcaacggcctgcta.ctgttatagtcggtcgtt.cggttgaccgcatttatc
BamMC406_3114,10225-10385,BamMC406_3115               5  cggcgcaacggcctgcta.ctgttatagtcggtcgtt.cggttgaccgcatttatc
BCAM0009,11121-11278,BCAM0010                         5  cggcgcaacggcctgcta.ctgttatagtcggtcgtt.cggttgaccgcacttatc
Bcen_5158,2443932-2444089,Bcen_5159                   5  cggcgcaacggcctgcta.ctgttatagtcggtcgtt.cggttgaccgcatttatc
Bcen2424_5700,2822895-2823052,Bcen2424_5701           5  cggcgcaacggcctgcta.ctgttatagtcggtcgtt.cggttgaccgcatttatc
Bcenmc03_4536,1521532-1521689,Bcenmc03_4537           5  cggcgcaacggcctgcta.ctgttatagtcggtcgtt.cggttgaccgcatttatc
Bcep1808_3788,535946-536105,Bcep1808_3789             5  cagcgcgacgggctgcta.ctgttatagtcggtcgtt.cggttgaccgcatttatc
Bcep18194_B3179,3585716-3585876,Bcep18194_B3180       5  cggcgcaacggcctgcta.ctgttatagtcggtcgtt.cggttgaccgcatttatc
BMA10229_1430,1500010-1500176,BMA10229_1431           7  cggcaaggcagcttgcta.ctgttatagtcggccgtc.gggttcagcgtattttc
BMA10247_A0005,4695-4861,BMA10247_A0006               7  cggcaaggcagcttgcta.ctgttatagtcggccgtc.gggttcagcgtattttc
BMAA0004,4695-4861,BMAA0005                           7  cggcaaggcagcttgcta.ctgttatagtcggccgtc.gggttcagcgtattttc
BMASAVP1_1150,1168886-1169052,BMASAVP1_1151           7  cggcaaggcagcttgcta.ctgttatagtcggccgtc.gggttcagcgtattttc
Bmul_5321,2471573-2471737,Bmul_5322                   5  ccgcgcaacagcctgcta.ctgttatagtcggtcgtt.cggttgaccgcatttatc
Bphy_4158,1149857-1149999,Bphy_4159                  10  accatgcggcgcggcagc.ctgttatagtcggctcag.gtttaatcggaaatttc
BPSS0004,3555-3721,BPSS0005                           7  cggcaaggcagcttgcta.ctgttatagtcggccgtc.gggttcagcgtattttc
BTH_II0004,3559-3725,BTH_II0005                       7  cggcaaagcagcttgcta.ctgttatagtcggccgtc.gggtccagcgtattttc
BURPS1710b_A1509,1842634-1842800,BURPS1710b_A1510     7  cggcaaggcagcttgcta.ctgttatagtcggccgtc.gggttcagcgtattttc
BURPS668_A0004,3557-3723,BURPS668_A0005               7  cggcaaggcagcttgcta.ctgttatagtcggccgtc.gggttcagcgtattttc
Bxe_B3022,9240-9400,Bxe_B3021                         8  gtgcgaccacgaggcggc.ctgttatagtcggctgtc.agttttatcggatatttt
```

putative cepBOX preceding "sodium:dicarboxylate symporter"

```
Bamb_5059,2079485-2079816,Bamb_5060                   4  cttgccccgccgtctttc.ctttcagaattgaaagat.ggtccggcgtttgcgata
BamMC406_3204,106395-106725,BamMC406_3205             4  ctggtcccgccatccttc.ctttcagaattgaaagat.ggtccggcgtttgcgata
Bcen_5075,2359656-2360006,Bcen_5076                   8  gcaaatcaactcgcgggg.ctttcacatttgaaagcc.ggtgcaccatttgcgtta
Bcen2424_5784,2906976-2907326,Bcen2424_5785           8  gcaaatcaactcgcgggg.ctttcacatttgaaagcc.ggtgcaccatttgcgtta
Bcenmc03_4393,1368186-1368536,Bcenmc03_4394           7  gcaaatcaactcgcgggg.ctttcagatttgaaagcc.ggtgcaccatttgcgtta
Bcep18194_B3086,3488557-3489161,Bcep18194_B3087       7  gcagatcaacccgctcga.ctttcagatttgaaagcc.gggctgtcatttgcgtta
BMA10229_A0368,394678-394915,BMA10229_A0369          10  ccatgccccgggcgtgcg.ctttcgttttgaaagcg.gcccggtcaattgaggta
BMA10247_0792,766179-766416,BMA10247_0793            10  ccatgccccgggcgtgcg.ctttcgttttgaaagcg.gcccggtcaattgaggta
BMASAVP1_A1711,1702998-1703235,BMASAVP1_A1712        10  ccatgccccgggcgtgcg.ctttcgttttgaaagcg.gcccggtcaattgaggta
BPSL1850,2203763-2204940,BPSL1851                    10  ccatgccccgggcgtgcg.ctttcgttttgaaagcg.gcccggtcaattgaggta
BTH_I2492,2849515-2850580,BTH_I2493                   8  ccctgtcccgagcgaacg.ctttcattttgaaagcg.ccccggtcaattgaggta
BURPS668_1832,1797429-1797768,BURPS668_1833          10  ccatgccccgggcgtgcg.ctttcgttttgaaagcg.gcccggtcaattgaggta
```

end of page 14

putative cepBOX preceding either "Rhs element Vgr protein" (and possibly putative "transmembrane protein" from the same operon) or "major facilitator transporter"

```
Bamb_5209,2246938-2247682,Bamb_5210                   21  gttaaaggcgtgaattcgctaaat.cgcgcggcggagattagcaagttt.ttctggatcgtaaaatgtattttt
BamMC406_3371,288914-289659,BamMC406_3372             21  gttaagcgcgggaatccgctgaat.cgcgcggcggagattagcaagttt.ttctgactcggaaaatgtattttt
Bcen_1411,1557865-1558212,Bcen_1412                   21  agcgtttgccgtcaatcatccgaa.agtacattaaaagttaatagaatc.aatggattggtatttatttattg
Bcen2424_6417,625623-626133,Bcen2424_6418             21  agcgtttgccgtcaatcatccgaa.agtacattaaaagttaatagaatc.aatggattggtatttatttattg
Bcep18194_A5512,2605666-2605965,Bcep18194_A5513       21  aatctggtcaggagcattacacaa.agcgctctaaatcctttcaaaatt.tcgtcacctgcctgacaaatttga
BPSS0077A,88593-88920,BPSS0078                        11  ggcggacggctcgacaacgtcgag.gtgcctgtcaagattggcaggtca.atgtcgtctttgggtgcgctcagc
BPSS1213,1638742-1640243,BPSS1214                     11  ggcggacggctcgacaacgtcgag.gtgcctgtcaagattggcaggtca.atgtcgtctttgggtgcgctcagc
BTH_I3224,3670997-3671317,BTH_I3225                   11  cttgagcgtaccaaaaggcgatat.tggcctgtcaaccttgacaggtcg.cctccaggttgtcgactcgtccgt
BTH_II0088,97359-97679,BTH_II0089                     11  cttgagcgtaccaaaaggcgatat.tggcctgtcaaccttgacaggtcg.cctccaggttgtcgactcgtccgt
BURPS1106A_A1615,1585666-1585985,BURPS1106A_A1616     11  ggcggacggctcgacaacgtcgag.gtgcctgtcaagattggcaggtca.atgtcgtctttgggtacgctcaaa
BURPS1710b_A1581,1926299-1927566,BURPS1710b_A1582     21  ttcggagggcagcaggcggtggcc.taagcggctttagccatcaggtgc.tgctcgcgtatcggattgaattac
```

putative cepBOX preceding either "Rhs element Vgr protein" (and possibly putative "transmembrane protein" from the same operon) or "major facilitator transporter"

```
Bamb_5209,2246938-2247682,Bamb_5210                   21  gtgaattcgctaaatcgcgcggcg.gagattagcaagttttctggatc.gtaaaatgtattttatttgaga
BamMC406_3371,288914-289659,BamMC406_3372             21  gggaatccgctgaatcgcgcggcg.gagattagcaagttttctgactc.ggaaaatgtattttatttggga
Bcen2424_6417,625623-626133,Bcen2424_6418             20  catgctatcagctaggctggccgc.gaaccgtaaagaattgtccgggca.atggcctgggcgctgtgcgttgag
Bcep18194_A5512,2605666-2605965,Bcep18194_A5513       17  gtgctgcccgcctggtttttggc.gagcatatcaaatttgtcaggcag.gtgacgaaatttgaaaggattta
BPSS0077A,88593-88920,BPSS0078                        11  ggcggacggctcgacaacgtcgag.gtgcctgtcaagattggcaggtca.atgtcgtctttgggtgcgctcagc
BPSS1213,1638742-1640243,BPSS1214                     11  ggcggacggctcgacaacgtcgag.gtgcctgtcaagattggcaggtca.atgtcgtctttgggtgcgctcagc
BURPS1710b_A1581,1926299-1927566,BURPS1710b_A1582     11  ggcggacggctcgacaacgtcgag.gtgcctgtcaagattggcaggtca.atgtcgtctttgggtacgctcaaa
```

putative cepBOX preceding either "Rhs element Vgr protein" (and possibly putative "transmembrane protein" from the same operon) or "major facilitator transporter"

```
Bamb_5209,2246938-2247682,Bamb_5210                   20  attgttcggggagccgcaagagcg.ccgtcagcttcttttgtccgatta.aagcaggtatggtttttttaaaaac
BamMC406_3371,288914-289659,BamMC406_3372             19  gatcgttcggtcggctgcggggtg.cgatcagctgatttttgtccgatta.atgcgggcgtgtattttaaaaaac
Bcen_1411,1557865-1558212,Bcen_1412                   21  ctcaacgcacagcgcccaggccat.tgcccggacaattctttacggttc.gcggccagcctagctgatagcatg
Bcep18194_A5512,2605666-2605965,Bcep18194_A5513       21  ggcgggcagcactcccaggacgga.ccatcgggcgtgatttcccggtga.acaacgaagtaaaacaaaatcctg
BURPS1106A_A0099,88027-88346,BURPS1106A_A0100         11  ggcggacggctcgacaacgtcgag.gtgcctgtcaagattggcaggtca.atgtcgtctttgggtacgctcaaa
BURPS668_A0126,107904-108222,BURPS668_A0127           22  tcgacgtgtctggcgcacgcctcg.tggcgcgttaattttagcaagatc.aagacaaaaggcggccaaattaac
```

putative cepBOX preceding either "Rhs element Vgr protein" (and possibly putative "transmembrane protein" from the same operon) or "major facilitator transporter"

```
Bamb_5209,2246938-2247682,Bamb_5210                    9  ttaaagcaggtatggttt.tttaaaaactcgatttgt.cagacataaaggcgaacg
BamMC406_3371,288914-289659,BamMC406_3372             10  ttaatgcgggcgtgtatt.ttaaaaaactcgatttgt.cagacataaaaggcgggc
Bcenmc03_4520,1505888-1506591,Bcenmc03_4521           10  ttatctctatgtcacatt.ttggtaaactcctgcgta.accgtcgctatatagaac
Bcep18194_A5512,2605666-2605965,Bcep18194_A5513        9  caaaatttcgtcacctgc.ctgacaaatttgatatgc.tcgccaaaaaaccaggcg
BPSS0077A,88593-88920,BPSS0078                         6  cccaaagacgacattgac.ctgccaatcttgacaggc.acctcgacgttgtcgagc
BPSS1213,1638742-1640243,BPSS1214                     10  agtgcatcgagttgagct.ttcagttcctcgtaagtt.gccacaatgactctccaa
BTH_II0088,97359-97679,BTH_II0089                      5  ccaaaaggcgatattggc.ctgtcaaccttgacaggt.cgcctccaggttgtcgac
BURPS1106A_A0099,88027-88346,BURPS1106A_A0100          6  cccaaagacgacattgac.ctgccaatcttgacaggc.acctcgacgttgtcgagc
BURPS1106A_A1615,1585666-1585985,BURPS1106A_A1616      6  cccaaagacgacattgac.ctgccaatcttgacaggc.acctcgacgttgtcgagc
BURPS1710b_A0207,312907-313226,BURPS1710b_A0208        6  cccaaagacgacattgac.ctgccaatcttgacaggc.acctcgacgttgtcgagc
BURPS1710b_A1581,1926299-1927566,BURPS1710b_A1582     10  acggcatgcctggttgtt.ttggaaaggcggtcgat.cctggcgtactgaaatga
BURPS668_A0126,107904-108222,BURPS668_A0127            6  cccaaagacgacattgac.ctgccaatcttgacaggc.ccctcgacgttgtcgagc
```

putative cepBOX preceding either "Rhs element Vgr protein" (and possibly putative "transmembrane protein" from the same operon) or "major facilitator transporter"

```
Bamb_5209,2246938-2247682,Bamb_5210                   10  gtttttaaaaactcgat.ttgtcagacataaaggcg.aacgactgcctgttgcag
BamMC406_3371,288914-289659,BamMC406_3372              8  tattttaaaaaactcgat.ttgtcagacataaaaggc.gggcgactgcgtgttgca
Bcen2424_6417,625623-626133,Bcen2424_6418             10  gatcccgatggatcggtc.atgtgcgacacgcatgct.atcagctaggctggccgc
BTH_I3224,3670997-3671317,BTH_I3225                    5  ccaaaaggcgatattggc.ctgtcaaccttgacaggt.cgcctccaggttgtcgac
BTH_II0088,97359-97679,BTH_II0089                      5  ccaaaaggcgatattggc.ctgtcaaccttgacaggt.cgcctccaggttgtcgac
BURPS1106A_A0099,88027-88346,BURPS1106A_A0100         10  ccgccttttgtcttgacc.ttgctaaaattaacgcgc.cacgagacgtgcgccaga
BURPS1106A_A1615,1585666-1585985,BURPS1106A_A1616     10  ccgccttttgtcttgacc.ttgctaaaattaacgcgc.cacgagacgtgcgccaga
BURPS1710b_A0207,312907-313226,BURPS1710b_A0208       10  ccgccttttgtcttgacc.ttgctaaaattaacgcgc.cacgagacgtgcgccaga
BURPS1710b_A1581,1926299-1927566,BURPS1710b_A1582     10  ccgccttttgtcttgacc.ttgctaaaattaacgcgc.cacgagacgtgcgccaga
BURPS668_A0126,107904-108222,BURPS668_A0127           10  ccgccttttgtcttgatc.ttgctaaaattaacgcgc.cacgaggcgtgcgccaga
```

putative cepBOX preceding either "Rhs element Vgr protein" (and possibly putative "transmembrane protein" from the same operon) or "major facilitator transporter"

```
Bamb_5209,2246938-2247682,Bamb_5210                    9  atggcgcgttcgccggtt.gttccatacccgatcggc.ggccgcgcacctatcgtt
BamMC406_3371,288914-289659,BamMC406_3372              9  atggcgcgttcgccggtt.gttccatacccgatcggc.ggccccgcacctatcgtt
Bcen_1411,1557865-1558212,Bcen_1412                   10  tagttagcgcgatggaag.gtgcaacgaatgctcgat.gtgttgccggctgatccg
Bcen2424_6417,625623-626133,Bcen2424_6418             10  gcctagctgatagcatgc.gtgtcgcacatgaccgat.ccatcgggatcgtcgcgc
Bcenmc03_4520,1505888-1506591,Bcenmc03_4521           10  aaccgtcgctatatagaa.cagcaatatccggcgggt.cataacgaatcaaactga
Bcep18194_A5512,2605666-2605965,Bcep18194_A5513       10  ctttgaaagattgtgaag.ttttaaatctggtcagga.gcattacacaaagcgctc
BPSS0077A,88593-88920,BPSS0078                         7  cccaaagacgacattgac.ctgccaatcttgacaggc.acctcgacgttgtcgagc
BPSS1213,1638742-1640243,BPSS1214                      7  cccaaagacgacattgac.ctgccaatcttgacaggc.acctcgacgttgtcgagc
BTH_I3224,3670997-3671317,BTH_I3225                    5  ccaaaaggcgatattggc.ctgtcaaccttgacaggt.cgcctccaggttgtcgac
BTH_II0088,97359-97679,BTH_II0089                      5  ccaaaaggcgatattggc.ctgtcaaccttgacaggt.cgcctccaggttgtcgac
BURPS1106A_A0099,88027-88346,BURPS1106A_A0100          7  cccaaagacgacattgac.ctgccaatcttgacaggc.acctcgacgttgtcgagc
BURPS1106A_A1615,1585666-1585985,BURPS1106A_A1616      7  cccaaagacgacattgac.ctgccaatcttgacaggc.acctcgacgttgtcgagc
BURPS1710b_A0207,312907-313226,BURPS1710b_A0208        7  cccaaagacgacattgac.ctgccaatcttgacaggc.acctcgacgttgtcgagc
BURPS1710b_A1581,1926299-1927566,BURPS1710b_A1582      9  cgccaggatcgaccgcct.tttccaaaacaaccaggc.atgccgtcttaagcttgt
BURPS668_A0126,107904-108222,BURPS668_A0127            7  cccaaagacgacattgac.ctgccaatcttgacaggc.ccctcgacgttgtcgagc
```

end of page 15

putative cepBOX preceding either "Rhs element Vgr protein" (and possibly putative "transmembrane protein" from the same operon) or "major facilitator transporter"

```
Bamb_5209,2246938-2247682,Bamb_5210                    8  aaaatacattttacgatc.cagaaaaacttgctaatc.tccgccgcgcgatttagc
BamMC406_3371,288914-289659,BamMC406_3372              8  aaaatacattttccgagt.cagaaaaacttgctaatc.tccgccgcgcgattcagc
Bcen_1411,1557865-1558212,Bcen_1412                    9  gtcaatcatccgaaagta.cattaaaagttaatagaa.tcaatggattggtattta
Bcen2424_6417,625623-626133,Bcen2424_6418              9  gtcaatcatccgaaagta.cattaaaagttaatagaa.tcaatggattggtattta
Bcenmc03_4520,1505888-1506591,Bcenmc03_4521           10  gtgttcttcgtcggataa.catgaaaaattgctaatc.ggcattccggatcgttat
Bcep18194_A5512,2605666-2605965,Bcep18194_A5513       10  agcatgactctcaggatt.ttgttttacttcgttgtt.caccgggaaatcacgccc
BPSS0077A,88593-88920,BPSS0078                         6  cccaaagacgacattgac.ctgccaatcttgacaggc.acctcgacgttgtcgagc
BPSS1213,1638742-1640243,BPSS1214                      6  cccaaagacgacattgac.ctgccaatcttgacaggc.acctcgacgttgtcgagc
BTH_II0088,97359-97679,BTH_II0089                      5  ccaaaaggcgatattggc.ctgtcaaccttgacaggt.cgcctccaggttgtcgac
BURPS1106A_A0099,88027-88346,BURPS1106A_A0100          6  cccaaagacgacattgac.ctgccaatcttgacaggc.acctcgacgttgtcgagc
BURPS1106A_A1615,1585666-1585985,BURPS1106A_A1616      6  cccaaagacgacattgac.ctgccaatcttgacaggc.acctcgacgttgtcgagc
BURPS1710b_A0207,312907-313226,BURPS1710b_A0208        6  cccaaagacgacattgac.ctgccaatcttgacaggc.acctcgacgttgtcgagc
BURPS1710b_A1581,1926299-1927566,BURPS1710b_A1582      6  cccaaagacgacattgac.ctgccaatcttgacaggc.acctcgacgttgtcgagc
BURPS668_A0126,107904-108222,BURPS668_A0127           10  cttccaagcggaaccatc.ctggcctactggctcgtt.ccatgcgcgcgagagcga
```

putative cepBOX preceding either "Rhs element Vgr protein" (and possibly putative "transmembrane protein" from the same operon) or "major facilitator transporter"

```
Bamb_5209,2246938-2247682,Bamb_5210                   22  aaatggcggccgcctttccggttg.caggcgtcgaattcaatcagccga.cgcccgaacgataggtgcgcggcc
BamMC406_3371,288914-289659,BamMC406_3372             19  aagtggcggccgcctttccggttg.caggcgtcgaattcaatcagccga.cgcccgaacgataggtgcggggcc
Bcen_1411,1557865-1558212,Bcen_1412                   22  aactttaatgtactttcggatga.ttgacggcaaacgctgtcatatct.caacgcacagcgcccaggccattg
Bcen2424_6417,625623-626133,Bcen2424_6418             12  gatggatcggtcatgtgcgacacg.catgctatcagctaggctggccgc.gaaccgtaaagaattgtccgggca
BPSS0077A,88593-88920,BPSS0078                        18  gctgagcgcacccaaagacgacat.tgacctgccaatcttgacaggcac.ctcgacgttgtcgagccgtccgcc
BPSS1213,1638742-1640243,BPSS1214                     22  actaacgaccggaatgaatgttta.tttcctgtgaattaaacccgttga.tcatcgatagcttggtaaggagat
BTH_I3224,3670997-3671317,BTH_I3225                   22  gctctcgcgcccatggagcgagcc.agtccgccaaaaaatttccgcttt.gaaggtccatttcggggcctggga
BTH_II0088,97359-97679,BTH_II0089                     22  gctctcgcgcccatggagcgagcc.agtccgccaaaaaatttccgcttt.gaaggtccatttcggggcctggga
BURPS1106A_A0099,88027-88346,BURPS1106A_A0100         22  cattcgtggaacgagccagtaggc.caagctggttccgcttggaaggtc.catttcggggcttgggttgttgcg
BURPS1106A_A1615,1585666-1585985,BURPS1106A_A1616     22  cattcgtggaacgagccagtaggc.caagctggttccgcttggaaggtc.catttcggggcttgggttgttgcg
BURPS1710b_A0207,312907-313226,BURPS1710b_A0208       22  cattcgtggaacgagccagtaggc.caagctggttccgcttggaaggtc.catttcggggcttgggttgttgcg
BURPS1710b_A1581,1926299-1927566,BURPS1710b_A1582     21  gcgcgccctcggccgcaccaggtt.tcgccagtcatcggattccgatac.tttggaggcagtggaccgcgccgc
BURPS668_A0126,107904-108222,BURPS668_A0127           11  ggcggacggctcgacaacgtcgag.gggcctgtcaagattggcaggtca.atgtcgtctttgggtacgctcagc
```

putative cepBOX preceding either "Rhs element Vgr protein" (and possibly putative "transmembrane protein" from the same operon) or "major facilitator transporter"

```
Bamb_5209,2246938-2247682,Bamb_5210                   20  attggtccgcgtgcacgcgccgga.gcgtcggtgcatgaagccaggtcc.gttgtcttgtgtgcgatgcgacgc
BamMC406_3371,288914-289659,BamMC406_3372             20  attggtccgcgtgcacgcgccgca.gcgtcggtgcatgaagccaggtcc.gttgtcttgtgtgcgatgcgacgc
Bcen2424_6417,625623-626133,Bcen2424_6418             22  aaatccgtggcgcgccgtttgact.gcagtcggtatgtgtgtcagcccg.gcgcgcgacgatcccgatggatcg
Bcenmc03_4520,1505888-1506591,Bcenmc03_4521           21  atccacgccagcaagcccccccg.caccctgttcttgctgcggcccg.atctgcgggcttcgtcgagattgt
Bcep18194_A5512,2605666-2605965,Bcep18194_A5513       16  accgggaaatcacgcccgatggtc.cgtcctgggagtgctgcccgcctg.gtttttggcgagcatatcaaatt
BPSS0077A,88593-88920,BPSS0078                        11  ggcggacggctcgacaacgtcgag.gtgcctgtcaagattggcaggtca.atgtcgtctttgggtgcgctcagc
BPSS1213,1638742-1640243,BPSS1214                     11  ggcggacggctcgacaacgtcgag.gtgcctgtcaagattggcaggtca.atgtcgtctttgggtgcgctcagc
BTH_I3224,3670997-3671317,BTH_I3225                   19  tccccacggcgcgtagatttatc.aacgctgaggcaaatggcggccaa.attaacgaagattcaccatcgcgc
BTH_II0088,97359-97679,BTH_II0089                     19  tccccacggcgcgtagatttatc.aacgctgaggcaaatggcggccaa.attaacgaagattcaccatcgcgc
BURPS1106A_A0099,88027-88346,BURPS1106A_A0100         11  ggcggacggctcgacaacgtcgag.gtgcctgtcaagattggcaggtca.atgtcgtctttgggtacgctcaaa
BURPS1106A_A1615,1585666-1585985,BURPS1106A_A1616     11  ggcggacggctcgacaacgtcgag.gtgcctgtcaagattggcaggtca.atgtcgtctttgggtacgctcaaa
BURPS1710b_A0207,312907-313226,BURPS1710b_A0208       11  ggcggacggctcgacaacgtcgag.gtgcctgtcaagattggcaggtca.atgtcgtctttgggtacgctcaaa
BURPS1710b_A1581,1926299-1927566,BURPS1710b_A1582     11  ggcggacggctcgacaacgtcgag.gtgcctgtcaagattggcaggtca.atgtcgtctttgggtacgctcaaa
BURPS668_A0126,107904-108222,BURPS668_A0127           11  ggcggacggctcgacaacgtcgag.gggcctgtcaagattggcaggtca.atgtcgtctttgggtacgctcagc
```

putative cepBOX preceding either "FAD-dependent pyridine nucleotide-disulphide oxidoreductase" (and possibly LysR family transcriptional regulator from operon) or "BadM/Rrf2 family transcriptional regulator"

```
Bamb_5305,2345722-2345863,Bamb_5306                  9   ggcggtcaactcatgaaa.cacgagaagttgcatgat.aggtcgatggtatcattc
BamMC406_3486,413917-414059,BamMC406_3487            9   gcgcgtcaactcatgaaa.cgcgagaagttgcatgat.aggtcgatggtatcattc
BCAM0598,665635-665775,BCAM0599                      9   ggcggccaactcatgaaa.cacgagaagttgcatgat.agggcgatggtatcattc
Bcen2424_3571,415146-415246,Bcen2424_3572           10   ggcggccaactcatgaaa.cacgagcagttgcatgat.agggcggtgatatcattc
Bcenmc03_3948,878034-878200,Bcenmc03_3949            9   ggcggtcaactcatgaaa.cacgagaagttgcatgat.agggcgatggtatcattc
BMA10229_0566,610609-610737,BMA10229_0567           10   gtttgtcaaatcaaagca.cttgcattgttacgtgtg.cgacgcagtggtagattg
BMA10247_A1000,944492-944620,BMA10247_A1001         10   gtttgtcaaatcaaagca.cttgcattgttacgtgtg.cgacgcagtggtagattg
BMAA1310,1415387-1415515,BMAA1311                   10   gtttgtcaaatcaaagca.cttgcattgttacgtgtg.cgacgcagtggtagattg
BMASAVP1_0292,314546-314617,BMASAVP1_0293            8   gctgtctccttaacgtaa.ttttaaaagttacgaaag.aacgcgacgtttgtcaaa
BPSS0918,1212398-1212526,BPSS0919                    8   gctgtctccttaacgtac.ttttaaaagttacgaaag.aacgcgacgtttgtcaaa
BTH_II1477,1746394-1746464,BTH_II1478                5   cgttgcctccttatgtac.ttttcaaagttacgagat.agagcgaggtttgtcaaa
BURPS1106A_A1261,1194085-1194213,BURPS1106A_A1262    8   gctgtctccttaacgtac.ttttaaaagttacgaaag.aacgcgacgtttgtcaaa
BURPS1710b_A2515,3033018-3033727,BURPS1710b_A2517    8   gctgtctccttaacgtac.ttttaaaagttacgaaag.aacgcgacgtttgtcaaa
BURPS668_A1334,1256881-1257008,BURPS668_A1336        7   gctgtctccttaacgtac.ttttaaaagttacgagag.aacgcgacgtttgtcaaa
```

putative cepBOX preceding either "FAD-dependent pyridine nucleotide-disulphide oxidoreductase" (and possibly LysR family transcriptional regulator from operon) or "BadM/Rrf2 family transcriptional regulator"

```
Bamb_5305,2345722-2345863,Bamb_5306                  9   cggctcccactctcgaaa.ctcctgaagttgcatgaa.tgataccatcgacctatc
BamMC406_3486,413917-414059,BamMC406_3487            9   gggctcccactctcgaaa.ctccttaagttgcatgaa.tgataccatcgacctatc
BCAM0598,665635-665775,BCAM0599                      9   gctccccttctctcgaaa.cttttgaagttgcatgaa.tgataccatcgccctatc
Bcen2424_3571,415146-415246,Bcen2424_3572           10   ggcggccaactcatgaaa.cacgagcagttgcatgat.agggcggtgatatcattc
Bcenmc03_3948,878034-878200,Bcenmc03_3949            9   agctcccctctctcgaaa.cttctgaagttgcatgaa.tgataccatcgccctatc
Bcep18194_B2547,2897837-2897931,Bcep18194_B2548      9   atcatgcaacttctcgtg.tttcatgagttgagtgga.tcgccaccgggacacatc
BMA10229_0566,610609-610737,BMA10229_0567            8   gctgtctccttaacgtaa.ttttaaaagttacgaaag.aacgcgacgtttgtcaaa
BMA10247_A1000,944492-944620,BMA10247_A1001          8   gctgtctccttaacgtaa.ttttaaaagttacgaaag.aacgcgacgtttgtcaaa
BMAA1310,1415387-1415515,BMAA1311                    8   gctgtctccttaacgtaa.ttttaaaagttacgaaag.aacgcgacgtttgtcaaa
BMASAVP1_0291,313065-313480,BMASAVP1_0292            8   ggttataccaggccgccc.attccagcgttgccggtt.cgacggattcctgcgcca
BPSS0918,1212398-1212526,BPSS0919                   10   gtttgtcaaatcaaagca.cttgcattgttacgtgtg.cgacgcagtggtagattg
BTH_II1477,1746394-1746464,BTH_II1478                5   cgttgcctccttatgtac.ttttcaaagttacgagat.agagcgaggtttgtcaaa
BURPS1106A_A1261,1194085-1194213,BURPS1106A_A1262   10   gtttgtcaaatcaaagca.cttgcattgttacgtgtg.cgacgcagtggtagattg
BURPS1710b_A2515,3033018-3033727,BURPS1710b_A2517   10   gatcgcccactgtgatgg.cgccgaaacgtgcgagca.gcaaggcctgcgcttcgc
```

putative cepBOX preceding either "hypothetical protein" or "hypothetical protein"

```
Bamb_5423,2472045-2472345,Bamb_5424                 10   gattgcgcgatggccggc.gtgacgcaatcgtcatgt.tcgggtcatgcaagtgag
BCAM0716,794908-795643,BCAM0717                      7   cgtgatcgtgatcgcgag.atgaaaaatgcgtcagat.tgcggaggcaagaacgcg
Bcen2424_3682,535698-535903,Bcen2424_3683            7   cgtgatcgtgatcgcgag.atgaaaaatgcgtcagat.tgcgggggcaagaaggc
BMA10229_0404,407782-408190,BMA10229_0405           10   atgccgcaccgcgcgcag.atgacggatttgtcatcg.cacgctcatggttgcgta
BMASAVP1_0007,7190-7598,BMASAVP1_0008               10   atgccgcaccgcgcgcag.atgacggatttgtcatcg.cacgctcatggttgcgta
Bmul_6175,15795-15999,Bmul_6176                      9   tggacggactcggtcgat.atgacgcaatcgtcatgt.tcgcgtcacggatgcgat
BTH_II1378,1626742-1628183,BTH_II1379               10   atcgcatgctccgcctcg.acgacatctttgtcatgt.tcggctcacgttcgcgca
BURPS1710b_A0027,53598-54891,BURPS1710b_A0028       20   atgccgcaccgcgcgcag.atgacggatttgtcatcg.cacgctcatggttgcgta
Bxe_B0711,2577519-2578179,Bxe_B0710                  5   cctgttcgtaactcggta.atgacagtctagaaaggt.aggcccttgagaataagt
```

putative cepBOX preceding "protein of unknown function DUF883, ElaB"

```
Bamb_5535,2606602-2606938,Bamb_5536                  5   agtggggccggacagcgg.ttgaaaaaatttcgagat.cgagcgaagcgacataaa
BamMC406_3710,674070-674407,BamMC406_3711            5   gatggggccggacagcgg.ttgaaaaaatttcgagat.cgagcgaaacgacataag
BCAM0844,931041-931337,BCAM0845                      8   cgcggggccgcgcagcgg.ttgaaaaaatttcgcgtg.tcggggcggtacagcgtg
Bcen_4553,1809340-1809636,Bcen_4554                  6   cacggggcggcgcagcgg.ttgaaaaaatttcgcgtt.tcgcggcgatacagcgtg
Bcen2424_3809,671485-671781,Bcen2424_3810            6   cacggggcggcgcagcgg.ttgaaaaaatttcgcgtt.tcgcggcgatacagcgtg
Bcenmc03_3714,622075-622371,Bcenmc03_3715            6   cacggggcggcgcagcgg.ttgaaaaaatttcgcgtt.tcgcggcgatacagcgtg
Bcep18194_B2286,2612340-2612624,Bcep18194_B2287      9   tacggggcggcacggcga.ttgaaaaaaattcgcatt.ccgggcgaagcggcatat
Bmul_4928,2053628-2054209,Bmul_4929                 10   cagcgcagcaggcgcatg.gtgcaattgcatcaaggt.aaaccatgcaacggcact
Bphy_2790,3143205-3143553,Bphy_2791                 10   tcggcagcgcgtgcgcct.cagtcaaatttacaacgg.atgaagcgaatcacgcga
BTH_II0531,631523-632098,BTH_II0532                 10   aacgccgacgcaacgaaa.cgtaaaaaattccgccgt.cgattcgagcacgcggcc
```

putative cepBOX preceding either "mannose-1-P guanylyltransferase/mannose-6-P isomerase" or "sugar transferase" (and possibly "UDP-glucose 6-dehydrogenase", "polysaccharide export", BceD & BceF from operon)

```
Bamb_5545,2617575-2617953,Bamb_5546                  6   agaatttccgattatttt.tcgaaatacttgtgcgct.tgaggatgaatttttctta
BamMC406_3722,690205-690643,BamMC406_3723            6   agaatttccgattatttt.tcgaaatacttgtgcgct.tgaggatgaatttttctta
BCAM0854,942080-943947,BCAM0855                      6   agaatttccgattatttt.tcgaaatacttgtgcgct.tgaggggaatttttcttg
Bcen_4543,1798187-1798554,Bcen_4544                  6   agaatttccgattatttt.tcgaaatacttgtgcgct.tgaggatgaatttttcttg
Bcen2424_3819,682567-682934,Bcen2424_3820            6   agaatttccgattatttt.tcgaaatacttgtgcgct.tgaggatgaatttttcttg
Bcenmc03_3704,610824-611254,Bcenmc03_3705            6   agaatttccgattatttt.tcgaaatacttgtgcgct.tgaggatgaatttttcttg
Bcep1808_4200,1008038-1008464,Bcep1808_4201          6   agaatttccgattatttt.tcgaaatacttgtgcgct.tgagagtggatttttcttg
Bcep18194_B2276,2600965-2601392,Bcep18194_B2277      6   agaatttccgattatttt.tcgaaatacttgtgcgct.tgaggaggaatttttcttg
Bmul_4919,2044810-2045179,Bmul_4920                  6   agaattgccgattatttt.tcgaaatacttgtgcgct.tgcggcaggaattttctt
Bphy_1056,1186223-1186809,Bphy_1057                  9   attaattggcgactaatt.tatcaatgcttgaataat.taaaaataaatggaataa
BPSS1834,2495587-2496041,BPSS1835                    5   aaaatttccgattatttt.tagaaatacttgtgcgct.tgcgggaggaattttctt
BTH_II0542,644659-645086,BTH_II0543                  5   aaaatttccgattatttt.tagaaatacttgtgcgct.tgcgggaggaattttctt
BURPS1710b_A0918,1179619-1180046,BURPS1710b_A0919    5   aaaatttccgattatttt.tagaaatacttgtgcgct.tgcgggaggaattttctt
```

putative cepBOX preceding either "MarR family transcriptional regulator" (and possibly "fusaric acid resistance protein region", "RND family efflux transporter MFP subunit" from the same operon) or "GntR family transcriptional regulator" (and possibly "threonine dehydratase", "ectoine utilization protein EutC", "peptidase M24", "Succinylglutamate desuccinylase/aspartoacylase"... from the same operon)

```
Bamb_6016,569073-569481,Bamb_6017                    6   ttcggcaatgacactccg.gtgacaaaaccgatagtt.tgctatcgaatgtttttg
BamMC406_5772,299547-299954,BamMC406_5773            5   tgcggcgatgacatcccg.gtgacaaaactgatagtt.tgctatcgaatgtttttg
Bcen_5811,360782-361102,Bcen_5812                    7   atgcgacgtgtcatccgg.gtgacaaaaacgatagtt.tgctatcgaatgtttttg
Bcen2424_6175,338600-338920,Bcen2424_6176            7   atgcgacgtgtcatccgg.gtgacaaaaacgatagtt.tgctatcgaatgtttttg
Bcenmc03_6655,724699-725021,Bcenmc03_6656            7   atgcgacgtgtcatccgg.gtgacaaaaacgatagtt.tgctatcgaatgtttttg
Bcep1808_5465,15284-15648,Bcep1808_5466              9   aagctgcaagacatccgc.atgacaaaaacggtagtt.tgctatcgaattactttg
Bcep18194_C7720,1373198-1373505,Bcep18194_C7721      7   gagcgacgtgtcatcagg.gtgacaaaaacgatagtt.tgctatcgaattaatttg
```

end of page 17

putative cepBOX preceding "sodium/hydrogen exchanger"

```
Bamb_6042,603753-603969,Bamb_6043                     10  gccgcccgcctgtaggtc.cttccagtatcgccacgc.ttagccgcgctataggat
BamMC406_5812,348216-348432,BamMC406_5813             10  gccgcccgcctgtaggtc.cttccagtatcgccacgc.ttagccgcgctataggat
BMA1002,1054986-1055989,BMA1004                       10  aacatgcgcgcgtgatcc.cggtcgaatccgaccggt.taccgtgtgaggggagca
BMA10229_0450,496286-496492,BMA10229_0451             10  gatatacttggccgaaat.atggcgaattcgccggtt.ttcgcggtgccccgaatc
BMA10247_A1125,1058729-1058935,BMA10247_A1126         10  gatatacttggccgaaat.atggcgaattcgccggtt.ttcgcggtgccccgaatc
BMASAVP1_0173,205804-206010,BMASAVP1_0174             10  gatatacttggccgaaat.atggcgaattcgccggtt.ttcgcggtgccccgaatc
BPSS1178,1587189-1587667,BPSS1179                     10  gcctgcccgcgccggtgg.ttgaaagagcggcgagcc.ggcgcgcatgcgcggcgg
BTH_II1228,1451229-1451867,BTH_II1229                  9  gagtcattcgactatcag.atttgatagttgctccgg.tcgagggtttgcattgta
BURPS1106A_A1572,1534474-1534654,BURPS1106A_A1574      5  ataaaagaagccgaccga.ctatcagatttgatagtt.gttccggccgaggctttg
BURPS1710b_A0143,191564-191820,BURPS1710b_A0144        5  ataaaagaagccgaccga.ctatcagatttgatagtt.gttccggccgaggctttg
BURPS668_A1654,1599431-1599952,BURPS668_A1656          5  ataaaagaagccgaccga.ctatcagatttgatagtt.gttccggccgaggctttg
```

putative cepBOX preceding "beta-lactamase"

```
Bamb_6102,661151-661335,Bamb_6103                      9  ggccgcgccgcgcggaga.ctgctgcagccggccggt.cgaagcaggactatccgc
BCAS0155,173795-174405,BCAS0156                        9  ggccactcacccgttttc.cgggaaaacctgccggtt.ttaacagtatcgaatccg
Bcen_5712,248104-248446,Bcen_5713                      9  cgcccgtgattccgtccc.atgcgatgctcgatcggt.ccgcctccatcgaccggt
Bcen_5713,249617-250240,Bcen_5714                      5  cggccgccggattcgatg.ctgttaaaaccgacaggt.tttcgtggaaaacggggg
Bcen2424_6076,225922-226264,Bcen2424_6077              9  cgcccgtgattccgtccc.atgcgatgctcgatcggt.ccgcctccatcgaccggt
Bcen2424_6077,227435-228058,Bcen2424_6078              5  cggccgccggattcgatg.ctgttaaaaccgacaggt.tttcgtggaaaacggggg
Bcenmc03_6561,618701-619325,Bcenmc03_6562              5  cggccgccggattcgatg.ctgttaaaaccgacaggt.ttttcgtggaaaacgggg
Bcep18194_C7532,1153220-1153831,Bcep18194_C7533       10  cgctgcttcgcccgccag.ccgaaagaaccgccaggg.tcccgcgcgcatcgcctg
Bmul_6008,745824-746065,Bmul_6009                      8  tgcggctcgcggccgcgc.gcgccgaagctgttcgtt.ccactgtccgacaggaac
```

putative cepBOX preceding "Pyrrolo-quinoline quinone" (and possibly "cytochrome c, class I", "High potential iron-sulfur protein", "sigma factor 70 SigI"... from the same operon)

```
Bamb_6162,729017-729788,Bamb_6163                      8  acagtcgcgggcggagtg.ttgcgaaacatgacagtg.ggttgatggggtgcggg
BamMC406_5895,434204-434975,BamMC406_5896              8  acagccgcgggcggagtg.ttgcgaaacatgacagtg.ggttgatggtggcgcggg
BCAM2367,2663105-2663517,BCAM2368                      5  accgtcgcgcgtgaagtg.ttgccaaacatgacaggt.ggcgcagtcggccggaca
Bcen_3242,317217-317647,Bcen_3243                      5  accggcgcgcgcgaagtg.ttgccaaacatgacaggt.ggtgcagtccgcggaca
Bcen2424_5125,2162013-2162443,Bcen2424_5126            5  accggcgcgcgcgaagtg.ttgccaaacatgacaggt.ggtgcagtccgcggaca
Bcenmc03_5155,2244048-2244475,Bcenmc03_5156            5  accggcgcgcgcgaagtg.ttgccaaacatgacaggt.ggcgcagtccgcggaca
Bmul_5971,702880-703372,Bmul_5972                      8  agcgtgctgtgcgcactg.ttgcgaaacatgacagtg.cgggtggtgcgcacggtg
Bphy_7191,1843348-1843744,Bphy_7192                   10  actgtccactgctccacg.atgcaacacgcgatatgt.gcctacgccacgcgcaag
Bxe_B0306,3033984-3034341,Bxe_B0305                    8  cattcgagcgcccatgtg.ttgctaaaaatgacagtt.gcccggccgaccaggcaa
```

putative cepBOX preceding "Pyrrolo-quinoline quinone" (and possibly "cytochrome c, class I", "High potential iron-sulfur protein", "sigma factor 70 SigI"... from the same operon)

```
Bamb_6162,729017-729788,Bamb_6163                      8  gccgcatgcttgttgccg.ctggcgaactcaaatgat.cgcgcgcggtgacacagt
BamMC406_5895,434204-434975,BamMC406_5896              8  tcggtatgcttcttgccg.ctgtcgacctcaaacgat.cgcgtgcgatgacacagc
BCAM2367,2663105-2663517,BCAM2368                      5  accgtcgcgcgtgaagtg.ttgccaaacatgacaggt.ggcgcagtcggccggaca
Bcen_3242,317217-317647,Bcen_3243                      5  accggcgcgcgcgaagtg.ttgccaaacatgacaggt.ggtgcagtccgcggaca
Bcen2424_5125,2162013-2162443,Bcen2424_5126            5  accggcgcgcgcgaagtg.ttgccaaacatgacaggt.ggtgcagtccgcggaca
Bcenmc03_5155,2244048-2244475,Bcenmc03_5156            5  accggcgcgcgcgaagtg.ttgccaaacatgacaggt.ggcgcagtccgcggaca
Bmul_5971,702880-703372,Bmul_5972                      8  agtgcgcacagcacgctg.ttcccgaacgcaacagat.gcgcgccgcggccgacca
Bphy_7191,1843348-1843744,Bphy_7192                   10  gcgcgtcgcacgtgtttc.ttgtcgttatcgaatgtg.cgagaagcgtgccatcga
Bxe_B0306,3033984-3034341,Bxe_B0305                    5  caagggccgctctttctc.gtgattgcatcgcacgat.cgtcggccatcgtgtccc
```

end of page 18

putative cepBOX preceding either "short-chain dehydrogenase/reductase SDR" (and possibly "ThiJ/PfpI domain-containing protein" from the same operon) or "lipase, class 3"

```
Bamb_6261,852957-853347,Bamb_6262                  11  catcgcgcgcgcggtacggcggaa.caacctgtcgagattggcaggtac.cgtcggcctgaaacggcctcacgg
BamMC406_5956,506038-506428,BamMC406_5957          11  catggcgcacgcggtacggcggaa.caacctgtcgaggttggcaggtac.cgacggtctgtcacggtcggcgt
BPSL1636,1901940-1902195,BPSL1637                  22  gttgcgcgccccgaaacgaggcca.cgcgcgggccatgcgggcggttgc.ggaaaatatcgccaacctcccggc
BPSL1637,1903267-1904964,BPSL1642                  22  cgcccgcggcatgagcgtgcgcga.gattcagacgtttctggccgaaag.ctaaggcactgagatctcgcccga
BURPS1106A_2073,2068331-2068544,BURPS1106A_2074    22  gttgcgcgccccgaaacgaggcca.cgcgcgggccatgcgggcggttgc.ggaaaatatcgccaacctcccggc
BURPS668_2014,2011618-2011738,BURPS668_2015        22  gttgcgcgccccgaaacgaggcca.cgcacgggccatgcgggcggttgc.ggaaaatatcgccaacctcccggc
```

putative cepBOX preceding either "GCN5-related N-acetyltransferase" (and possibly "D-2-hydroxyacid dehydrogenase" from operon) or "haloacid dehalogenase" (and possibly "chemotaxis transducer" from operon)

```
Bamb_6350,958822-959300,Bamb_6351                   4  atccgcgcccgtgggtcg.ctggcagagtcgataggt.cacttctacccatccgga
BamMC406_6054,617950-618428,BamMC406_6055           4  atccgcgcccgtgggtcg.ctggcagagtcgataggt.catttctacccatccgga
BCAS0385,451767-452180,BCAS0386                     6  atccgtgccgccgccgcg.ctggcagagtgaacaggt.catttccacccatccgga
Bcen_6447,1076611-1077077,Bcen_6448                 7  atccgcgccgcacccgcg.ctggcagagtcgacctgt.catttccacccatccgga
Bcen2424_6682,913757-914223,Bcen2424_6683           7  atccgcgccgcacccgcg.ctggcagagtcgacctgt.catttccacccatccgga
Bcenmc03_6279,283001-283452,Bcenmc03_6280           7  atccgcgccgcacccgcg.ctggcagagtcgacctgt.catttccacccatccgga
Bxe_B1493,1737302-1737593,Bxe_B1492                 9  tgcaaagcacttgctccg.atcccaaacacgatagtt.gccgttttcgccaatgag
```

putative cepBOX preceding "muconate & chloromuconate cycloisomerases" (and possibly "Muconolactone delta-isomerase", "catechol 1,2-dioxygenase", "Rieske (2Fe-2S) domain-containing protein"... from same operon)

```
Bamb_6587,1278475-1279300,Bamb_6588                 9  aatacttttgggcgcggt.ttttgatacctggtgggt.attgaaattcgctttggg
BamMC406_6302,894323-895389,BamMC406_6303           8  aatacttttgggcgtggt.ttttgatacctgctgggt.attgaaattgggtttgag
BCAS0498,568745-569291,BCAS0499                     9  aatacttttgggcaggat.ttttgatacctggtgggt.attgaaatccggccgtgg
Bcep18194_C7043,610943-611575,Bcep18194_C7044       5  aatacttttaggcggctt.tttcaatacctgacaggt.attgaaattcggggatat
```

putative cepBOX preceding "muconate & chloromuconate cycloisomerases" (and possibly "Muconolactone delta-isomerase", "catechol 1,2-dioxygenase", "Rieske (2Fe-2S) domain-containing protein"... from same operon)

```
Bamb_6587,1278475-1279300,Bamb_6588                10  ccggctgaaacgggtggc.ctgcctgagtcgatcagg.gttttcctgagggcacaa
BamMC406_6302,894323-895389,BamMC406_6303          10  ccggctgaaacgggtagc.ctgcctgagtcgatcagg.gttttcctgagggcgaaa
BCAS0498,568745-569291,BCAS0499                     7  ttcgcgcccacggccgga.tttcaatacccaccaggt.atcaaaaatcctgcccaa
Bcep18194_C7043,610943-611575,Bcep18194_C7044       5  cccacgaatatccccgaa.tttcaatacctgtcaggt.attgaaaaagccgcctaa
```

putative cepBOX preceding "muconate & chloromuconate cycloisomerases" (and possibly "Muconolactone delta-isomerase", "catechol 1,2-dioxygenase", "Rieske (2Fe-2S) domain-containing protein"... from same operon)

```
Bamb_6587,1278475-1279300,Bamb_6588                 7  tccgcgtcccaaagcgaa.tttcaatacccaccaggt.atcaaaaaccgcgcccaa
BamMC406_6302,894323-895389,BamMC406_6303           8  ttcgcgtctcaaacccaa.tttcaatacccagcaggt.atcaaaaaccacgcccaa
BCAS0498,568745-569291,BCAS0499                     7  ttcgcgcccacggccgga.tttcaatacccaccaggt.atcaaaaatcctgcccaa
Bcep18194_C7043,610943-611575,Bcep18194_C7044       5  cccacgaatatccccgaa.tttcaatacctgtcaggt.attgaaaaagccgcctaa
```

2.3. Potential cepBOXes predicted for genes found in the cepI mutant screen

putative cepBOX preceding pyruvate dehydrogenase (and possibly dihydrolipoamide acetyltransferase, dihydrolipoamide dehydrogenase (Bamb_2172) from the same operon)

```
Bamb_2174,2390063-2390335,Bamb_2175                    7  acgggcccgcgcaactga.attttcgaattatgagat.cttttctcgtaatgcgaa
Bcep1808_2217,2458841-2459112,Bcep1808_2218            6  acggccgcgcgcaacaaa.attttcgaattgtgagat.cttttctcgtaatgcgga
Bcep18194_A5443,2515995-2516267,Bcep18194_A5444        6  acgggcctgcgcaacaaa.attttcgaattgtgagat.cttttctcgcaatgcgga
BMA10247_1502,1494966-1495261,BMA10247_1504            7  cggacggcgcgcaacaaa.attttcgaattatgagat.catttctcgcaatgcgga
Bmul_1132,1247275-1247546,Bmul_1133                    7  cgggccgcacgcaacaaa.attttcgaattatgagat.cttttctcgtaatgcgga
BPSL2301,2773266-2773507,BPSL2302                      7  cgggccgcgcgcaacaaa.attttcgaattatgagat.catttctcgtaatgtgga
BTH_I1863,2106292-2106533,BTH_I1864                    7  cacgcaacgcgcaacaaa.attttcgaattatgagat.catttctcgcgatatgaa
BURPS1106A_2667,2630794-2631092,BURPS1106A_2669        7  cgggcggcgcgcaacaaa.attttcgaattatgagat.catttctcgtaatgcgga
BURPS668_2612,2584188-2584486,BURPS668_2613            7  cgggcggcgcgcaacaaa.attttcgaattatgagat.catttctcgtaatgcgga
Bxe_A1541,3211555-3211820,Bxe_A1540                    7  cgacgtccgcgcaacaaa.attttcgaattacgagat.tcattctcataatgcgga
```

putative cepBOX preceding hypothetical protein (and possibly carbonate dehydratase, sulfate transporter (Bamb_2297) from the same operon)

```
Bamb_2293,2530809-2530996,Bamb_2294                    8  cgccggcgcgcgccgtcc.gtgatacaactgaacggt.cagagtgttctggagaat
BamMC406_2172,2423925-2424115,BamMC406_2173            8  cgccggcgcgcgccgtcc.gtgatacaactgaacggt.cagagagctctggagaat
Bcen2424_2256,2511773-2512155,Bcen2424_2257            6  aaatggcgcaatcgcatg.ctttctaaattgaaaggt.tggatttcgactgatat
Bcep1808_2341,2600032-2600415,Bcep1808_2342            9  ttgcgcattttcgcgcaa.ttttcacaatggccattt.acgtatcgatgcgtaaat
BMA10247_0403,393848-394291,BMA10247_0404              6  tcggcgcggaaccaattt.ctttctaaattgaaaggt.tgttgtgagtgatattag
Bmul_1020,1116651-1117046,Bmul_1021                    6  aagcgacgcatcgacatg.ctttctgaattgaaaggt.tggtttcgactgatatta
Bphy_2015,2267419-2267599,Bphy_2016                    9  tattttgacatgcgtcgc.atgaaaagattgtcggcc.ggttcgcgcgccggcacg
BURPS1710b_1426,1501522-1501925,BURPS1710b_1427        6  tcggcgcggaaccaattt.ctttctaaattgaaaggt.tgttgtgagtgatattag
```

putative cepBOX preceding carbonate dehydratase (and possibly sulfate transporter (Bamb_2297) from the same operon)

```
Bamb_2294,2531510-2531902,Bamb_2296                    9  agggcataccctaatat.cagtcgaaattgaacctt.tcagttcagaaaggatcc
BCAL2351,2605106-2605488,BCAL2352                      6  aaatggcgcaagcgcatt.ctttctaaattgaaaggt.tgaatttcgactgatatt
Bcep1808_2340,2599339-2599518,Bcep1808_2341            8  cgcgggcacgcgcggtcc.gtgatacaactgaacggt.tagcgagttctggagaaa
Bcep1808_2341,2600032-2600415,Bcep1808_2342           10  gttccgtgcgttgaattg.gtgcaattgatgcacctt.gccgaatcgagttgaaac
BMA10247_0403,393848-394291,BMA10247_0404              9  gggggaactcgtaatcgg.aagttcaatttgaaatat.tctgttgcaatttatttt
BMA1838,1927971-1928375,BMA1839                        9  gggggaactcgtaatcgg.aagttcaatttgaaatat.tctgttgcaatttatttt
BMASAVP1_A1120,1104051-1104494,BMASAVP1_A1121          9  gggggaactcgtaatcgg.aagttcaatttgaaatat.tctgttgcaatttatttt
Bmul_1020,1116651-1117046,Bmul_1021                    6  aagcgacgcatcgacatg.ctttctgaattgaaaggt.tggtttcgactgatatta
BURPS668_1281,1252318-1252760,BURPS668_1282            9  gggggaactcgtaatcgg.aagttcaatttgaaatat.tctgttgcaatttatttt
Bxe_A3222,1374900-1375083,Bxe_A3221                    9  aatcttttgccgcagcgc.gtgtcaaaataatgcgtc.tcgatgataaaaaccgct
```

putative cepBOX preceding carbonate dehydratase (and possibly sulfate transporter (Bamb_2297) from the same operon)

```
Bamb_2294,2531510-2531902,Bamb_2296                    8  agggcataccctaatat.cagtcgaaattgaacctt.tcagttcagaaaggatcc
BamMC406_2173,2424629-2425021,BamMC406_2174            8  agggcataccctaatat.cagtcgaaattgaacctt.tcagttcagaaaggatcc
BCAL2351,2605106-2605488,BCAL2352                      6  aaatggcgcaagcgcatt.ctttctaaattgaaaggt.tgaatttcgactgatatt
Bcen_1645,1826512-1826616,Bcen_1646                    6  aaatggcgcaatcgcatg.ctttctaaattgaaaggt.tggatttcgactgatat
Bcep1808_2341,2600032-2600415,Bcep1808_2342            6  gttccgtgcgttgaattg.gtgcaattgatgcacctt.gccgaatcgagttgaaac
BMA10247_0403,393848-394291,BMA10247_0404              6  gggggaactcgtaatcgg.aagttcaatttgaaatat.tctgttgcaatttatttt
BMA1838,1927971-1928375,BMA1839                        6  gggggaactcgtaatcgg.aagttcaatttgaaatat.tctgttgcaatttatttt
BMASAVP1_A1120,1104051-1104494,BMASAVP1_A1121          6  gggggaactcgtaatcgg.aagttcaatttgaaatat.tctgttgcaatttatttt
Bmul_1020,1116651-1117046,Bmul_1021                    6  aagcgacgcatcgacatg.ctttctgaattgaaaggt.tggtttcgactgatatta
BPSL1203,1388571-1389013,BPSL1204                      6  gggggaactcgtaatcgg.aagttcaatttgaaatat.tctgttgcaatttatttt
BTH_I1052,1195433-1195867,BTH_I1053                    8  gggggaacttgtaatcgg.aagttcaatttgaaatat.tctgttgcaaattatttt
BURPS1106A_1288,1262772-1263713,BURPS1106A_1290        6  gggggaactcgtaatcgg.aagttcaatttgaaatat.tctgttgcaatttatttt
BURPS1710b_1426,1501522-1501925,BURPS1710b_1427        6  gggggaactcgtaatcgg.aagttcaatttgaaatat.tctgttgcaatttatttt
BURPS668_1281,1252318-1252760,BURPS668_1282           10  gggggaactcgtaatcgg.aagttcaatttgaaatat.tctgttgcaatttatttt
```

putative cepBOX preceding carbonate dehydratase (and possibly sulfate transporter (Bamb_2297) from the same operon)

```
Bamb_2294,2531510-2531902,Bamb_2296                    8  atgtggagcaataggatc.ctttctgaactgaaaggt.tcaatttcgactgatatt
BamMC406_2173,2424629-2425021,BamMC406_2174            8  aagtggagcaataggatc.ctttctgaactgaaaggt.tcaatttcgactgatatt
BCAL2351,2605106-2605488,BCAL2352                      6  aaatggcgcaagcgcatt.ctttctaaattgaaaggt.tgaatttcgactgatatt
Bcen_1645,1826512-1826616,Bcen_1646                    6  aaatggcgcaatcgcatg.ctttctaaattgaaaggt.tggattttcgactgatat
Bcen2424_2256,2511773-2512155,Bcen2424_2257            6  aaatggcgcaatcgcatg.ctttctaaattgaaaggt.tggattttcgactgatat
Bcenmc03_2280,2552354-2552736,Bcenmc03_2281            6  aaatggcgcgatcgcatg.ctttctaaattgaaaggt.tggattttcgactgatat
Bcep1808_2341,2600032-2600415,Bcep1808_2342            6  aacgtgagcggtcagatg.ctttctaaattgaaaggt.ttgattcgaccgatatta
Bcep18194_A5584,2682671-2683052,Bcep18194_A5585        6  aaatgacgcaatggcatt.ctttctaaattgaaaggt.ttggtttcgactgatatt
BMA10229_A0745,766683-767126,BMA10229_A0746            6  tcggcgcggaaccaattt.ctttctaaattgaaaggt.tgttgtgagtgatattag
BMA10247_0403,393848-394291,BMA10247_0404              6  tcggcgcggaaccaattt.ctttctaaattgaaaggt.tgttgtgagtgatattag
BMA1838,1927971-1928375,BMA1839                        6  tcggcgcggaaccaattt.ctttctaaattgaaaggt.tgttgtgagtgatattag
BMASAVP1_A1120,1104051-1104494,BMASAVP1_A1121          6  tcggcgcggaaccaattt.ctttctaaattgaaaggt.tgttgtgagtgatattag
Bmul_1020,1116651-1117046,Bmul_1021                    6  aagcgacgcatcgacatg.ctttctgaattgaaaggt.tggtttcgactgatatta
BPSL1203,1388571-1389013,BPSL1204                      6  tcggcgcggaaccaattt.ctttctaaattgaaaggt.tgttgtgagtgatattag
BTH_I1052,1195433-1195867,BTH_I1053                    6  tcgtcgcagaatcaatgg.ctttctaaactgaaaggt.tgttgtgagtgatattag
BURPS1106A_1288,1262772-1263713,BURPS1106A_1290        6  tcggcgcggaaccaattt.ctttctaaattgaaaggt.tgttgtgagtgatattag
BURPS1710b_1426,1501522-1501925,BURPS1710b_1427        6  tcggcgcggaaccaattt.ctttctaaattgaaaggt.tgttgtgagtgatattag
BURPS668_1281,1252318-1252760,BURPS668_1282            6  tcggcgcggaaccaattt.ctttctaaattgaaaggt.tgttgtgagtgatattag
```

putative cepBOX preceding hypothetical protein (and possibly haemagluttinin, ompA, hypothetical, hypothetical (4578), hypothetical, hypothetical from the same operon)

```
Bamb_4573,1527820-1527910,Bamb_4574                  10  tacgcgatgacggaagct.atgcaagaatctttcgtc.ttgtaatcgcataacaac
BamMC406_5097,2268307-2268398,BamMC406_5098          10  tacgcgatgacggaagct.atgcaagaatctttcgtc.ttgtaatcgcataacaac
BCAM2416,2720340-2720434,BCAM2417                     8  tacgcgatgacggaagct.gtgcaagaatctttcgat.ccgtaatcgcataacaat
Bcen_3145,200339-200867,Bcen_3146                     8  tacgcgatgacggaagct.gtgcaagaatctttcgat.ccgtaatcgcataacaat
Bcen2424_5221,2278797-2279325,Bcen2424_5222           8  tacgcgatgacggaagct.gtgcaagaatctttcgat.ccgtaatcgcataacaat
Bcenmc03_5049,2122001-2122529,Bcenmc03_5050           8  tacgcgatgacggaagct.gtgcaagaatctttcgat.ccgtaatcgcataacaat
Bcep1808_3477,182308-182399,Bcep1808_3478            10  tacgcgctgacgaaagct.atgcaagaatctttcgtc.ttgtaatcgtataacaat
BMA10229_0907,946025-946122,BMA10229_0908             8  tacgtgattcatcgagct.atgcaaaaatcgtcggct.cctgacatcacgatacca
BMA10247_A1876,1803789-1803886,BMA10247_A1877         8  tacgtgattcatcgagct.atgcaaaaatcgtcggct.cctgacatcacgatacca
BTH_II0962,1147506-1147677,BTH_II0963                 7  tacgtgattcctcgagct.atgcaagaatcgtccgct.cctgacatcacgatacca
BURPS1106A_A1936,1896264-1896575,BURPS1106A_A1938     8  tacgtgattcatcgagct.atgcaaaaatcgtcggct.cctgacatcacgatacca
BURPS1710b_A0452,623942-624081,BURPS1710b_A0453       8  tacgtgattcatcgagct.atgcaaaaatcgtcggct.cctgacatcacgatacca
BURPS668_A2032,1962837-1962934,BURPS668_A2033         8  tacgtgattcatcgagct.atgcaaaaatcgtcggct.cctgacatcacgatacca
```

putative cepBOX preceding hypothetical protein (and possibly haemagluttinin, ompA, hypothetical, hypothetical (4578), hypothetical, hypothetical from the same operon)

```
Bamb_4573,1527820-1527910,Bamb_4574                  10  tacgcgatgacggaagct.atgcaagaatctttcgtc.ttgtaatcgcataacaac
BamMC406_5097,2268307-2268398,BamMC406_5098          10  tacgcgatgacggaagct.atgcaagaatctttcgtc.ttgtaatcgcataacaac
BCAM2417,2721338-2722299,BCAM2418                     9  caaacaatccttactgaa.atgaaagaatgcaggtt.gcgcaaacgtttgctatt
Bcenmc03_5049,2122001-2122529,Bcenmc03_5050           9  tacgcgatgacggaagct.gtgcaagaatctttcgat.ccgtaatcgcataacaat
Bcep1808_3477,182308-182399,Bcep1808_3478            10  tacgcgctgacgaaagct.atgcaagaatctttcgtc.ttgtaatcgtataacaat
BMA10229_0907,946025-946122,BMA10229_0908             6  tacgtgattcatcgagct.atgcaaaaatcgtcggct.cctgacatcacgatacca
BMA10247_A1876,1803789-1803886,BMA10247_A1877         6  tacgtgattcatcgagct.atgcaaaaatcgtcggct.cctgacatcacgatacca
BPSS1428,1943663-1943760,BPSS1429                     6  tacgtgattcatcgagct.atgcaaaaatcgtcggct.cctgacatcacgatacca
Bxe_B1013,2240793-2240952,Bxe_B1012                   9  tcgcgtcggcgcgcagcg.ccgccggacttgtgcagt.ccggcatcggaatttgaa
```

putative cepBOX preceding haemagluttinin (and possibly ompA, hypothetical, hypothetical (4578), hypothetical, hypothetical from the same operon)

```
Bamb_4574,1528814-1529889,Bamb_4575                  10  cgtcacaacattacaaac.cagaaagaattacctcca.tcgcaaacgtttgcggtt
BamMC406_5098,2269302-2270331,BamMC406_5099          10  catcacaacattacaaac.cagaaagaattacctcaa.tcgcaaacgtttgcggtt
BCAM2417,2721338-2722299,BCAM2418                     9  tgccaggtaaattcgatt.ataaaataatctccaaat.acaaaaaccaattcaatt
Bcen_3145,200339-200867,Bcen_3146                     9  tacgcgatgacggaagct.gtgcaagaatctttcgat.ccgtaatcgcataacaat
Bcen2424_5221,2278797-2279325,Bcen2424_5222           9  tacgcgatgacggaagct.gtgcaagaatctttcgat.ccgtaatcgcataacaat
Bcenmc03_5049,2122001-2122529,Bcenmc03_5050           9  tacgcgatgacggaagct.gtgcaagaatctttcgat.ccgtaatcgcataacaat
Bmul_3478,401143-401982,Bmul_3479                    10  aaaccatccagataaacg.caccttaaatcaccaaat.tcaacgcactcgattatc
Bphy_3472,397635-397773,Bphy_3473                     8  gccacaaaatgacgggtt.atgacagaatagccggaa.taactagccaaagattcc
BURPS1106A_A1936,1896264-1896575,BURPS1106A_A1938     6  tacgtgattcatcgagct.atgcaaaaatcgtcggct.cctgacatcacgatacca
```

putative cepBOX preceding haemagluttinin (and possibly ompA, hypothetical, hypothetical (4578), hypothetical, hypothetical from the same operon)

```
Bamb_4574,1528814-1529889,Bamb_4575                  10  ccagaaagaattacctcc.atcgcaaacgtttgcggt.ttcattcttgttacacat
BamMC406_5098,2269302-2270331,BamMC406_5099          10  ccagaaagaattacctca.atcgcaaacgtttgcggt.ttcattcttgttacacat
BCAM2417,2721338-2722299,BCAM2418                     9  aaacgtttgctatttcat.ttgtcttactttgagtc.gattcgatctgttgccca
Bcen_3145,200339-200867,Bcen_3146                    10  ggtgacgtgacgaaaaag.gtcacagagtcaacaagg.tgagcccatgtgattgga
Bcenmc03_5048,2120136-2121097,Bcenmc03_5049           9  agcgcacggagtaatcct.ctaacaaaagagaccggt.gtgtggcgtgcacatttg
BMA10247_A1876,1803789-1803886,BMA10247_A1877         6  tacgtgattcatcgagct.atgcaaaaatcgtcggct.cctgacatcacgatacca
BPSS1428,1943663-1943760,BPSS1429                     6  tacgtgattcatcgagct.atgcaaaaatcgtcggct.cctgacatcacgatacca
Bxe_B1013,2240793-2240952,Bxe_B1012                   9  tcgcgtcggcgcgcagcg.ccgccggacttgtgcagt.ccggcatcggaatttgaa
```

putative cepBOX preceding haemagluttinin (and possibly ompA, hypothetical, hypothetical (4578), hypothetical, hypothetical from the same operon)

```
Bamb_4574,1528814-1529889,Bamb_4575                  10  gtgctacggacccatcgc.gcgcggtaattgaaacgt.cgtttcgaatttgttcta
BCAM2417,2721338-2722299,BCAM2418                    10  atgacttacgatggttgt.gtgcgatgggcaacagat.cgaatcgactcaaaagta
Bcen_3144,198474-199435,Bcen_3145                     9  cccttgataccgatgcca.gcgcaatcgtcaatagga.cgaatccgataaattgac
Bcen2424_5222,2280229-2281190,Bcen2424_5223           9  cccttgataccgatgcca.gcgcaatcgtcaatagga.cgaatccgataaattgac
Bcenmc03_5048,2120136-2121097,Bcenmc03_5049           9  cccttgataccgatgcca.gcgcaatcgtcaatagga.cgaatccgataaattgac
BPSS1429,1944661-1945121,BPSS1430                     7  tcgccgcccatcgagcgc.ttgccaaaatttccatgt.ttcgactattatcgaaac
BTH_II0961,1146303-1146647,BTH_II0962                10  acaaggcgcaacgtgtcg.gcgcgcgattcgacacgt.tgaatacaggcaaggact
```

putative cepBOX preceding haemagluttinin (and possibly ompA, hypothetical, hypothetical (4578), hypothetical, hypothetical from the same operon)

```
Bamb_4574,1528814-1529889,Bamb_4575                   9  tggatcgtgtttgatgc.attcgaaagtaatgtatt.tgaaagtacgtggaatgc
BCAM2417,2721338-2722299,BCAM2418                     9  acagtgataaatgaaccg.ttttgaaagttaaagaat.ggttaatgcatcgagttt
Bcen_3144,198474-199435,Bcen_3145                    10  acagtgataaatgaaggg.attggaaagttaaagaat.ggttaatgagccgtgttt
Bcen2424_5222,2280229-2281190,Bcen2424_5223          10  acagtgataaatgaaggg.attggaaagttaaagaat.ggttaatgagccgtgttt
Bcenmc03_5048,2120136-2121097,Bcenmc03_5049          10  acagtgataaatgaaggg.attggaaagttaaagaat.ggttaatgagccgtgttt
BURPS1106A_A1936,1896264-1896575,BURPS1106A_A1938     6  tacgtgattcatcgagct.atgcaaaaatcgtcggct.cctgacatcacgatacca
```

putative cepBOX preceding haemagluttinin (and possibly ompA, hypothetical, hypothetical (4578), hypothetical, hypothetical from the same operon)

```
Bamb_4574,1528814-1529889,Bamb_4575                  10  tggttaatggatcgtgtt.ttgatgcattcgaaagta.atgtatttgaaagtacgt
BamMC406_5098,2269302-2270331,BamMC406_5099           9  tagttaatggatcgtgtt.ttgatacattggaaagta.atgtatttgaaagtgcgt
BCAM2417,2721338-2722299,BCAM2418                    10  ttgacaaacagtgataaa.tgaaccgttttgaaagtt.aaagaatggttaatgcat
Bcen_3145,200339-200867,Bcen_3146                    10  tggcccaaaggccgcccg.gtacagttctcgaaagct.tccccgcgagaatgtgga
Bcenmc03_5049,2122001-2122529,Bcenmc03_5050          10  tggcccaaaggccgcccg.gtacagttctcgaaagct.tccccgcgagaatgtgga
Bcen_3144,198474-199435,Bcen_3145                    10  atgcgtgccgattccccc.ttgataccgatgccagcg.caatcgtcaataggacga
Bcenmc03_5048,2120136-2121097,Bcenmc03_5049          10  atgcgtgccgattccccc.ttgataccgatgccagcg.caatcgtcaataggacga
Bmul_3478,401143-401982,Bmul_3479                     9  atgtcgttgcgttgattt.tttgataatcgagtgcg.ttgaatttggtgatttaa
BMAA0560,570157-570627,BMAA0561                      10  tcgataatagtcgaaacg.tggaaatttggcaagcg.ctcgatgggcggcgaggc
BPSS1429,1944661-1945121,BPSS1430                    10  tcgataatagtcgaaaca.tggaaatttggcaagcg.ctcgatgggcggcgaggc
BMA10247_A1876,1803789-1803886,BMA10247_A1877         6  tacgtgattcatcgagct.atgcaaaaatcgtcggct.cctgacatcacgatacca
```

putative cepBOX preceding haemagluttinin (and possibly ompA, hypothetical, hypothetical (4578), hypothetical, hypothetical from the same operon)

```
Bamb_4574,1528814-1529889,Bamb_4575                   6  acagtgaaaaatggggcg.tttgaaaagttaaaagat.ggttaatggatcgtgttt
BamMC406_5098,2269302-2270331,BamMC406_5099           6  acagtgaaaaatgcggcg.tttgaaaagttaaaagat.agttaatggatcgtgttt
BCAM2417,2721338-2722299,BCAM2418                     9  gttttgatgtactgggtg.tgaattaatttgaaagat.gtaagaatgtgtcgtgta
Bcen_3144,198474-199435,Bcen_3145                    10  acagtgataaatgaaggg.attggaaagttaaagaat.ggttaatgagccgtgttt
Bcenmc03_5048,2120136-2121097,Bcenmc03_5049          10  acagtgataaatgaaggg.attggaaagttaaagaat.ggttaatgagccgtgttt
BPSS1429,1944661-1945121,BPSS1430                    10  ttcgataatagtcgaaac.atggaaattttggcaagc.gctcgatgggcggcgagg
```

```
putative cepBOX preceding "tryptophan halogenase, PrnA" (Bamb_4726) (and possibly PrnB, C and D from the same operon)

Bamb_4725,1718136-1718840,Bamb_4726                    10  gtcaatcaagtcgttcat.ttcgcattttatacaat.aaattcgttgacgcacac
BamMC406_5263,2476374-2477051,BamMC406_5264            10  gtcaattaaatcgttcat.ttcgcattttatacaat.aaattcgttggcgcacac
Bcep18194_C6705,220713-220993,Bcep18194_C6706           5  ggaaagcaaataaaaatc.ctgtcaaatttatcagtt.taatttaagtaatcgccg
BURPS1710b_A1114,1394092-1394966,BURPS1710b_A1115      10  ttctttcaagaaaaata.aatctttgttataaaat.atttcaccgatattttcc


putative cepBOX preceding "tryptophan halogenase, PrnA" (Bamb_4726) (and possibly PrnB, C and D from the same operon)

Bamb_4725,1718136-1718840,Bamb_4726                     9  aaagtaaatttctcgtttt.ttaaaaaattaaatcgtt.tgttatcggatactccgc
BamMC406_5263,2476374-2477051,BamMC406_5264             9  aaagtaaatttctcatttt.ttaaaaaattaaatcgtt.tgttatcggaatctccgc
Bcenmc03_6982,1094614-1095393,Bcenmc03_6983             9  aaaatgaatttctttgtt.tgataaaagtaaatcgtt.tggcgtgggatatttcat
Bcep18194_C6705,220713-220993,Bcep18194_C6706           4  ggaaagcaaataaaaatc.ctgtcaaatttatcagtt.taatttaagtaatcgccg


putative cepBOX preceding "tryptophan halogenase, PrnA" (Bamb_4726) (and possibly PrnB, C and D from the same operon)

Bamb_4725,1718136-1718840,Bamb_4726                    20  aacagcctcagacgaaagtctggg.actgcggccaatgatgtccggatt.ttgcgcggaggggcatctcttgc
BamMC406_5263,2476374-2477051,BamMC406_5264            20  aacagcctcggacgaaattccggg.actgcggccaatgatgtccggatt.ttgcgcggaggggcgtcccttgc
Bcenmc03_6982,1094614-1095393,Bcenmc03_6983            22  atgtatccccgcggacatgaaggc.catgcgatgaagacgagcaagttc.acgacagcacgggtcgcatttgca
Bcep18194_C6705,220713-220993,Bcep18194_C6706          11  taaatccgtcggaaagcaaataaa.aatcctgtcaaatttatcagttta.atttaagtaatcgccgacctattc
BURPS1710b_A1114,1394092-1394966,BURPS1710b_A1115      19  aacggtctcggatcaaactccggg.aatgcaggcaaggctgtccggata.ttgcgtagagggacgttcttcatt


putative cepBOX preceding "tryptophan halogenase, PrnA" (Bamb_4726) (and possibly PrnB, C and D from the same operon)

Bamb_4725,1718136-1718840,Bamb_4726                    22  gcaaaatccggacatcattggccg.cagtcccagacttcgtctgaggc.tgttccgctatcaatgaaaataga
BamMC406_5263,2476374-2477051,BamMC406_5264            21  gcaaaatccggacatcattggccg.cagtcccggaatttcgtccgaggc.tgttccgctttcaattcaaataga
Bcenmc03_6982,1094614-1095393,Bcenmc03_6983            21  tcgacccaaaaataatccgactcg.tcagcagagaaattcgtcatgatg.tatcccgcggacatgaaggccat
Bcep18194_C6705,220713-220993,Bcep18194_C6706          11  gaataggtcggcgattacttaaat.taaactgataaatttgacaggatt.tttatttgctttccgacggattta
BURPS1710b_A1114,1394092-1394966,BURPS1710b_A1115      20  tcggaaacggcgacgaccctattt.ctttccggcaaattcatccgtcca.ttccgcactttttatacaatgaatt


putative cepBOX preceding "tryptophan halogenase, PrnA" (Bamb_4726) (and possibly PrnB, C and D from the same operon)

Bamb_4725,1718136-1718840,Bamb_4726                    19  gcaagagatgcccctccgcgcaa.aatccggacatcattggccgcagt.cccagactttcgtctgaggctgtt
BamMC406_5263,2476374-2477051,BamMC406_5264            19  gcaagggacgcccctccgcgcaa.aatccggacatcattggccgcagt.cccggaatttcgtccgaggctgtt
Bcep18194_C6705,220713-220993,Bcep18194_C6706          11  taaatccgtcggaaagcaaataaa.aatcctgtcaaatttatcagttta.atttaagtaatcgccgacctattc
BURPS1710b_A1114,1394092-1394966,BURPS1710b_A1115      18  aatgaagaacgtccctctacgcaa.tatccggacagccttgcctgcatt.cccggagtttgatccgagaccgtt

end of page 22
```

putative cepBOX preceding hypothetical protein (Bamb_5109)

```
Bamb_5108,2137088-2138007,Bamb_5109                    6  ggccgggaatggaaaggg.ttcagaaaatcgaaagct.gaaacagtccgatttaat
BamMC406_3259,166869-167541,BamMC406_3260              6  cggccggaatggaagttg.ttcagaaaatcgaaagct.gaaacagttcgatttaat
Bcep1808_3909,678239-678532,Bcep1808_3910             10  cgcgccgcgcacgggaag.gttgtacagtcgagggct.gccgacgtgcgagcgcag
```

putative cepBOX preceding hypothetical protein (Bamb_5109)

```
Bamb_5108,2137088-2138007,Bamb_5109                    7  atgttgaaacgcgccggc.ctgacatacctgacaggc.gatcaatttcgagattgg
BamMC406_3259,166869-167541,BamMC406_3260              7  atatcgcaccgcactggc.ctgacatacctgacaggc.gatcaatttcgagattgg
Bcep1808_3909,678239-678532,Bcep1808_3910              8  tgcgcgcgcgtgcgcggc.ctgatacacctgacaggc.gaacatttccggattgg
Bcep18194_B2926,3307320-3307445,Bcep18194_B2927        6  gacgcccgatattgaagc.ctgatatagctgacaggc.gatgacttctcacaatga
```

putative cepBOX preceding hypothetical-DUF883 (5535) (and possibly hypothetical, hypothetical from same operon)

```
Bamb_5535,2606602-2606938,Bamb_5536                    5  agtggggccggacagcgg.ttgaaaaaatttcgagat.cgagcgaagcgacataaa
BamMC406_3710,674070-674407,BamMC406_3711              5  gatggggccggacagcgg.ttgaaaaaatttcgagat.cgagcgaaacgacataag
BCAM0844,931041-931337,BCAM0845                        8  cgcggggccgcgcagcgg.ttgaaaaaatttcgcgtg.tcgggcggtacagcgtg
Bcen_4553,1809340-1809636,Bcen_4554                    6  cacggggcggcgcagcgg.ttgaaaaaatttcgcgtt.tcgcggcgatacagcgtg
Bcen2424_3809,671485-671781,Bcen2424_3810              6  cacggggcggcgcagcgg.ttgaaaaaatttcgcgtt.tcgcggcgatacagcgtg
Bcenmc03_3714,622075-622371,Bcenmc03_3715              6  cacggggcggcgcagcgg.ttgaaaaaatttcgcgtt.tcgcggcgatacagcgtg
Bcep18194_B2286,2612340-2612624,Bcep18194_B2287        9  tacggggcggcacggcga.ttgaaaaaaattcgcatt.ccgggcgaagcggcatat
Bmul_4928,2053628-2054209,Bmul_4929                   10  cagcgcagcaggcgcatg.gtgcaattgcatcaaggt.aaaccatgcaacggcact
Bphy_2790,3143205-3143553,Bphy_2791                   10  tcggcagcgcgtgcgcct.cagtcaaatttacaacgg.atgaagcgaatcacgcga
BTH_II0531,631523-632098,BTH_II0532                   10  aacgccgacgcaacgaaa.cgtaaaaaattccgccgt.cgattcgagcacgcggcc
```

putative cepBOX preceding hypothetical-DUF883 (5535) (and possibly hypothetical, hypothetical from same operon)

```
Bamb_5535,2606602-2606938,Bamb_5536                    5  agtggggccggacagcgg.ttgaaaaaatttcgagat.cgagcgaagcgacataaa
BamMC406_3710,674070-674407,BamMC406_3711              5  gatggggccggacagcgg.ttgaaaaaatttcgagat.cgagcgaaacgacataag
BCAM0844,931041-931337,BCAM0845                        8  cgcggggccgcgcagcgg.ttgaaaaaatttcgcgtg.tcgggcggtacagcgtg
Bcen_4553,1809340-1809636,Bcen_4554                    6  cacggggcggcgcagcgg.ttgaaaaaatttcgcgtt.tcgcggcgatacagcgtg
Bcen2424_3809,671485-671781,Bcen2424_3810              6  cacggggcggcgcagcgg.ttgaaaaaatttcgcgtt.tcgcggcgatacagcgtg
Bcenmc03_3714,622075-622371,Bcenmc03_3715              6  cacggggcggcgcagcgg.ttgaaaaaatttcgcgtt.tcgcggcgatacagcgtg
Bcep18194_B2286,2612340-2612624,Bcep18194_B2287        9  tacggggcggcacggcga.ttgaaaaaaattcgcatt.ccgggcgaagcggcatat
BPSS1844,2506266-2506641,BPSS1845                     10  cacctcctatgacgacgt.cttccatgatcgcgcggc.gcgaccacgtcgcgccgc
BTH_II0531,631523-632098,BTH_II0532                   10  aacgccgacgcaacgaaa.cgtaaaaaattccgccgt.cgattcgagcacgcggcc
BTH_II0532,633968-634335,BTH_II0533                   10  tgcgctgccggctcggcc.taaaaaacctggccagat.tacagattcacatctatt
```

end of page 23

3. Supplementary methods

3.1. CepBOX consensus used for each search (following the IUPAC code, lower case indicates low conservation)

```
   from Chambers et al. 2006:  1. htghhAdavTtghvagnT          2. bTgyhAdaGttghyagkT
   from Wei et al 2011:        1. cRnnCTGtYAaantTgnCAGntnc
```

3.2. RNAmotif descriptor example for the consensus 1 from Chambers et al. 2006 (SCORING RULES: minimum (best) score is 3, worst is 30, anything above 13 is rejected)

```
descr
ss (len=18)
score
{
  SCORE = 30;
  total_bonus = 0;
if(ss[1,1,1]!="g"){
 total_bonus--;
}
if(ss[1,2,1]!="c"){
 total_bonus--;
}
if(ss[1,2,1]=="t"){
 total_bonus--;
}
if(ss[1,3,1]!="a"){
 total_bonus--;
}
if(ss[1,3,1]=="g"){
 total_bonus--;
}
if(ss[1,4,1]!="g"){
 total_bonus--;
}
if(ss[1,5,1]!="g"){
 total_bonus--;
}
if(ss[1,6,1]=="a"){
 total_bonus--;
}
if(ss[1,6,1]=="a"){
 total_bonus--;
}
if(ss[1,7,1]!="c"){
 total_bonus--;
}
if(ss[1,8,1]=="a" || ss[1,8,1]=="t"){
 total_bonus--;
}
if(ss[1,8,1]=="a"){
 total_bonus--;
}
if(ss[1,9,1]!="t"){
 total_bonus--;
}
if(ss[1,10,1]=="t"){
 total_bonus--;
}
if(ss[1,10,1]=="t"){
 total_bonus--;
}
if(ss[1,11,1]!="g"){
 total_bonus--;
}
if(ss[1,11,1]=="t"){
 total_bonus--;
}
if(ss[1,12,1]=="a" || ss[1,12,1]=="g"){
 total_bonus--;
}
if(ss[1,12,1]=="g"){
 total_bonus--;
}
if(ss[1,13,1]!="g"){
 total_bonus--;
}
if(ss[1,14,1]!="t"){
 total_bonus--;
}
if(ss[1,15,1]!="g"){
 total_bonus--;
}
if(ss[1,15,1]=="a"){
 total_bonus--;
}
if(ss[1,16,1]=="a" || ss[1,16,1]=="g"){
 total_bonus--;
}
if(ss[1,16,1]=="g"){
 total_bonus--;
}
if(ss[1,18,1]=="t"){
 total_bonus--;
}
if(ss[1,18,1]=="t"){
 total_bonus--;
}
if(total_bonus > -20){
 REJECT;}
SCORE = SCORE + total_bonus;}
```

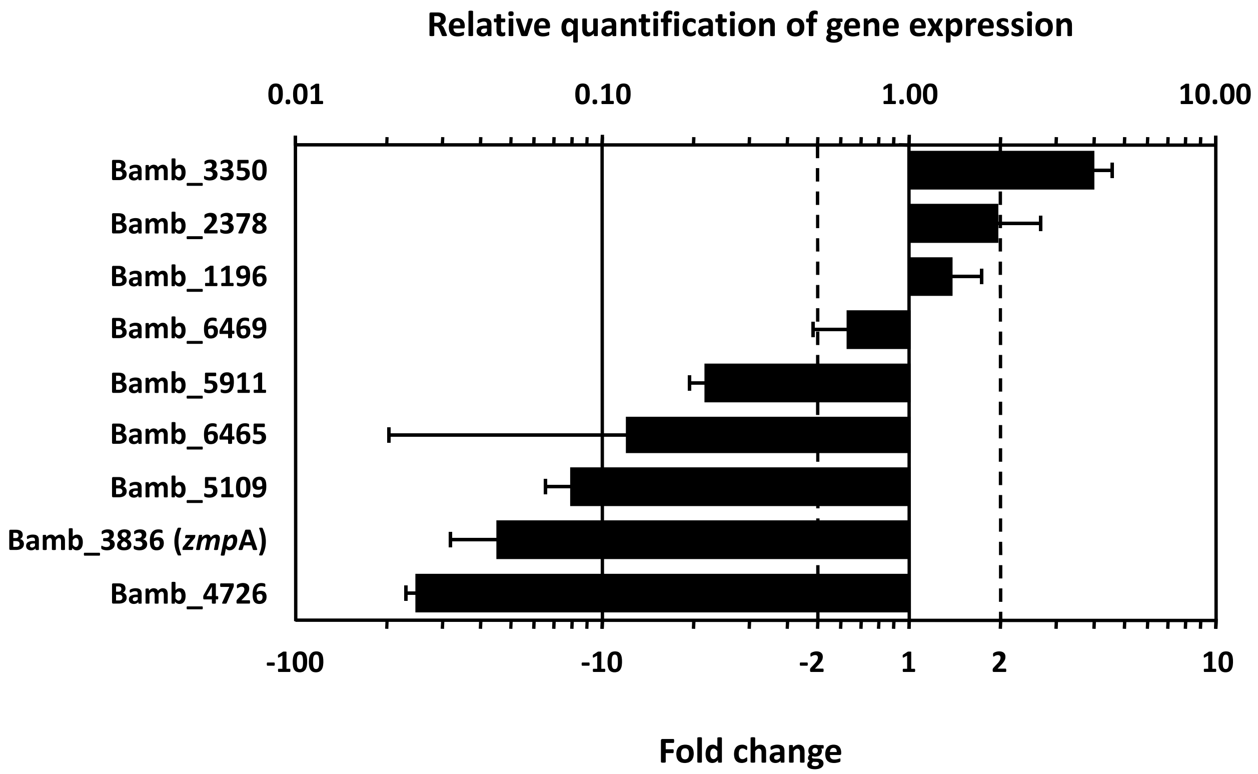
Relative quantification of gene expression
0.01
0.10
1.00
10.00
Bamb_3350
Bamb_2378
Bamb_1196
Bamb_6469
Bamb_5911
Bamb_6465
Bamb_5109
Bamb_3836 (zmpA)
Bamb_4726
-100
-10
-2
1
2
10
Fold change

Figure S2

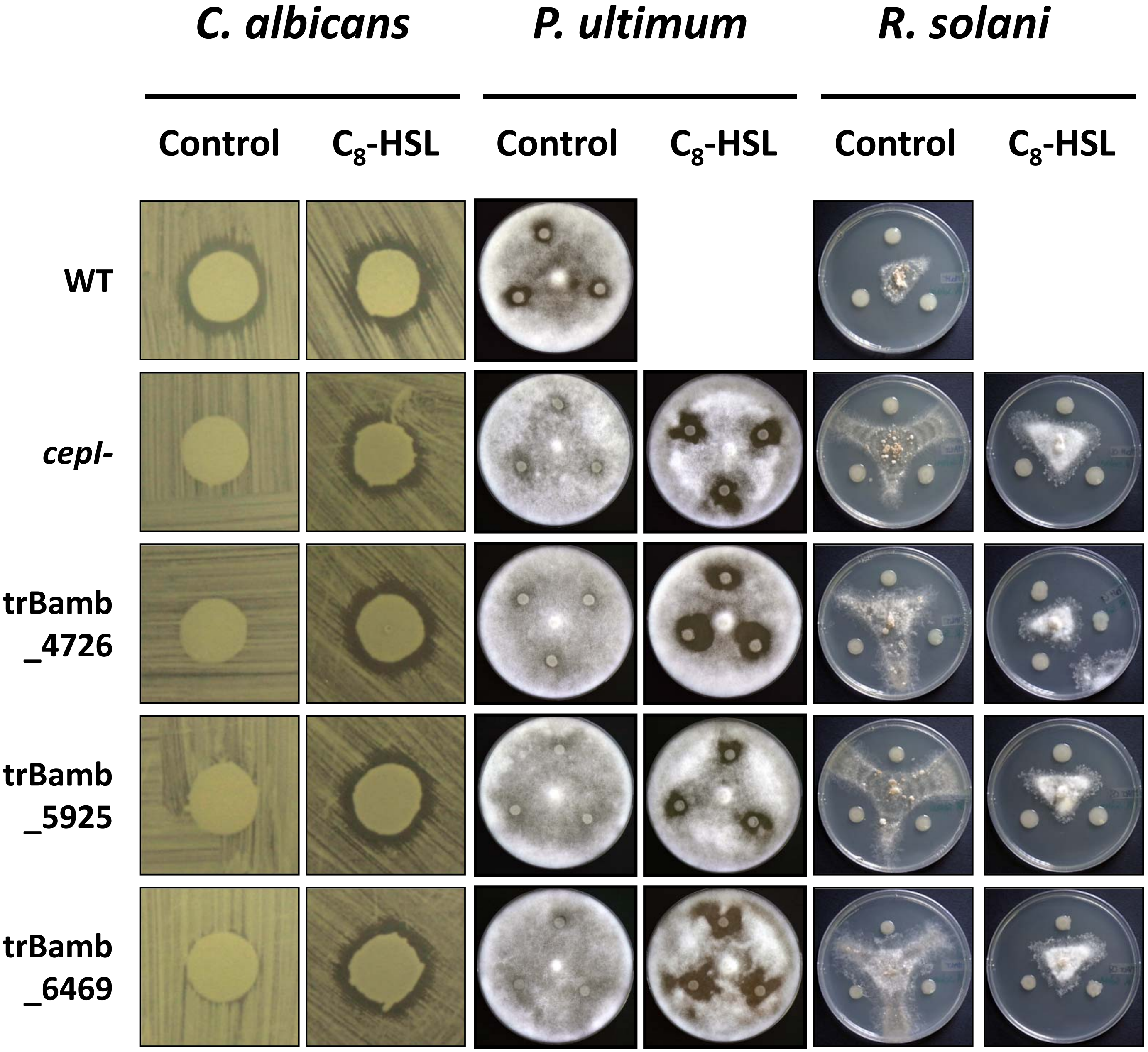
C. albicans
P. ultimum
R. solani
Control
C8-HSL
Control
C8-HSL
Control
C8-HSL
WT
cepI-
trBamb
_4726
trBamb
_5925
trBamb
_6469

Figure S3
